# Features of effective staff training programmes within school-based interventions targeting student activity behaviour: a systematic review and meta-analysis

**DOI:** 10.1186/s12966-022-01361-6

**Published:** 2022-09-24

**Authors:** Mairead Ryan, Olivia Alliott, Erika Ikeda, Jian’an Luan, Riikka Hofmann, Esther van Sluijs

**Affiliations:** 1 MRC Epidemiology Unit, University of Cambridge, Cambridge, UK; 2 Faculty of Education, University of Cambridge, Cambridge, UK

**Keywords:** School, Physical activity, Systematic review, Teacher, Implementation, Fidelity, Professional development

## Abstract

**Background:**

Evaluations of school-based activity behaviour interventions suggest limited effectiveness on students’ device-measured outcomes. Teacher-led implementation is common but the training provided is poorly understood and may affect implementation and student outcomes. We systematically reviewed staff training delivered within interventions and explored if specific features are associated with intervention fidelity and student activity behaviour outcomes.

**Methods:**

We searched seven databases (January 2015–May 2020) for randomised controlled trials of teacher-led school-based activity behaviour interventions reporting on teacher fidelity and/or students’ device-measured activity behaviour. Pilot, feasibility and small-scale trials were excluded. Study authors were contacted if staff training was not described using all items from the Template for Intervention Description and Replication reporting guideline. Training programmes were coded using the Behaviour Change Technique (BCT) Taxonomy v1. The Effective Public Health Practice Project tool was used for quality assessment. Promise ratios were used to explore associations between BCTs and fidelity outcomes (e.g. % of intended sessions delivered). Differences between fidelity outcomes and other training features were explored using chi-square and Wilcoxon rank-sum tests. Random-effects meta-regressions were performed to explore associations between training features and changes in students’ activity behaviour.

**Results:**

We identified 68 articles reporting on 53 eligible training programmes and found evidence that 37 unique teacher-targeted BCTs have been used (mean per programme = 5.1 BCTs; standard deviation = 3.2). The only frequently identified BCTs were ‘Instruction on how to perform the behaviour’ (identified in 98.1% of programmes) and ‘Social support (unspecified)’ (50.9%). We found moderate/high fidelity studies were significantly more likely to include shorter (≤6 months) and theory-informed programmes than low fidelity studies, and 19 BCTs were independently associated with moderate/high fidelity outcomes. Programmes that used more BCTs (estimated increase per additional BCT, *d*: 0.18; 95% CI: 0.05, 0.31) and BCTs ‘Action planning’ (1.40; 0.70, 2.10) and ‘Feedback on the behaviour’ (1.19; 0.36, 2.02) were independently associated with positive physical activity outcomes (*N* = 15). No training features associated with sedentary behaviour were identified (*N* = 11).

**Conclusions:**

Few evidence-based BCTs have been used to promote sustained behaviour change amongst teachers in school-based activity behaviour interventions. Our findings provide insights into why interventions may be failing to effect student outcomes.

**Trial registration:**

PROSPERO registration number: CRD42020180624

**Supplementary Information:**

The online version contains supplementary material available at 10.1186/s12966-022-01361-6.

## Background

Many school-based interventions have been delivered worldwide to promote physical activity and reduce sedentary behaviour (e.g. [1, 2]). Review-level evidence shows these interventions have largely failed to change students’ device-measured activity behaviour [3–5]. Research to date has largely focused on assessing students’ activity behaviour outcomes. Equal efforts have not been applied to determine how interventions have been implemented. Consequently, reasons for outcomes remain largely unknown and existing guidance for schools on how to promote physical activity or reduce sedentary behaviour is vague and underpinned by weak evidence (e.g. [6]).

Medical Research Council (MRC) guidance highlights the need to focus on the most important areas of uncertainty to interpret observed outcomes arising from interventions delivered within complex systems (e.g. educational systems) [7, 8]. In school-based interventions, successful implementation is often dependent on teachers, who are selected to deliver new instructional programmes (e.g. a new sports programme or ‘active’ lesson) (e.g. [9–11]). To facilitate this process, teachers are frequently enrolled onto training programmes, the broad aim of which is to change their teaching practice(s). However little is known about the training they receive [12], and how this effects their professional practice and student outcomes.

The most recent review to examine staff training within school-based activity behaviour interventions was conducted in 2015 [12]. Lander and colleagues evaluated features of training associated with significant changes in self-reported fundamental movement skills and/or physical activity within a physical education lesson. They found that training which is one day or more in length, delivered using multiple formats, and comprised of both subject and pedagogical content was associated with positive student outcomes. However, due to the prevalence of poor reporting across studies, the authors could not determine more specific training features that were causally related to desired outcomes. Hence, little is known on how to design training programmes to optimise intervention implementation (e.g. fidelity) and outcomes (e.g. activity behaviour).

To support the development of evidence-based teacher professional development, effective features of training programmes must be identified. This requires training features to be adequately described. ‘Behaviour change techniques’ (BCTs) offer a means of breaking down variable training programmes into observable, replicable, and irreducible features [13]. Specifying training programmes in terms of BCTs alongside features such as duration enables nuanced but rigorous evidence synthesis, and comparison with the wider professional development literature (e.g. [14–16]).

Many school-based intervention studies have been published since Lander and colleagues conducted the search for their review in 2015 [12]. The quality of reporting and underlying evidence may have improved since this time, given the greater availability of reporting guidelines (e.g. [17]) and use of device-based activity monitors (e.g. [18]). We therefore aimed to build on their review, and, in line with Cochrane guidance [19] reconsidered all elements of the review questions and scope. We aimed to determine, more specifically, which teacher-targeted BCTs have been used within school-based activity behaviour interventions that included staff training, and how their use and other training features are associated with intervention fidelity and students’ device-measured outcomes. Operational definitions are outlined in Table 1.

**Table 1:** Terms and definitions adopted for the current review

Term	Definition
Behaviour change technique	“An observable, replicable, and irreducible component of an intervention designed to alter or redirect causal processes that regulate behaviour; that is, a technique that is proposed to be an ‘active ingredient’” [13].
Fidelity	“The extent to which the intervention is delivered as intended” [8].
Staff training	Any set of activities aimed at changing teaching practice(s).
Activity behaviour	Any activity behaviour across the intensity spectrum, including physical activity and sedentary behaviour [20].
Physical activity	“Any body movement generated by the contraction of skeletal muscles that raises energy expenditure above resting metabolic rate. It is characterised by its modality, frequency, intensity, duration, and context of practice” [21].
Sedentary behaviour	“Any waking behaviours characterised by an energy expenditure ≤1.5 metabolic equivalent of tasks, while in a sitting, reclining, or lying posture” [22].
An intervention	Single or multiple components (e.g. contents and/or design features) of a programme that aim to effect one or more changes in a defined group of participants (e.g. school staff, students, parents).
A study	“A defined group of participants and one or more interventions and outcomes”. A study may have more than one output, peer-reviewed or otherwise, to report information about the protocol, analysis plan, process evaluation or observed outcomes [23].

## Review questions (RQs)

1. What BCTs have been used in staff training programmes to change student activity behaviour?
2. Is there an association between staff training features, including BCTs, and intervention fidelity?
3. Is there an association between staff training features, including BCTs, and changes in students’ device-measured activity behaviours?

## Methods

This review is reported in accordance with the 2020 Preferred Reporting Items for Systematic reviews and Meta-Analyses [24]. The review protocol was prospectively registered on PROSPERO (CRD42020180624).

## Literature search

The search strategy and terms were based on the inclusion and exclusion criteria (Table 2), and developed in collaboration with an experienced librarian. The sensitivity and specificity of combinations of free-text terms and database subject headings were tested using MEDLINE (via Ovid). Search terms and operators were subsequently translated and iteratively tested on additional databases identified as relevant (Education Resources Information Center, Applied Social Sciences Index and Abstracts, Embase (via Ovid), Scopus, Web of Science, SPORTDiscus). Searches were run on 15 May 2020 and limited to articles published since 1 January 2015 to avoid inclusion of studies assessed in the Lander review [12] and to focus resources on the highest quality data available to address the review’s aims. No language or geographic limitations were applied. Additional file 1 outlines search terms used and numbers of records identified.

**Table 2:** Study inclusion and exclusion criteria for systematic review

	Inclusion	Exclusion
**Population:**	School staff participating in an intervention aimed at changing any student activity behaviour across the intensity spectrum	Interventions targeting pre-school and/or pre-service teachers Interventions targeting mostly special student populations
**Intervention(s), exposure:**	Any staff training (at least one behaviour change technique must have been identified)	Staff training aimed at extramural school staff behaviour (e.g. training for teacher-led after-school interventions)
**Comparator(s)/control:**	Any control condition described	
**Outcomes:**	Staff fidelity (any quantitative measure), and/or any device-measured student activity behaviour assessed at both baseline and follow-up	Studies that do not report on outcomes after training was first introduced
**Study design**^a^**:**	Any experimental design ^a^Any randomised controlled design (determined by descriptions of the study design rather than its label)	^a^Feasibility, pilot, or small-scale studies (defined as ≤100 students at baseline) (determined based on the title, abstract and methods sections of study publications reporting on outcomes)

^a^Denotes criteria was applied during second round of full-text screening

## Screening

Search results were imported into EndNote X7 for deduplication (Clarivate, Philadelphia, PA). Remaining records were imported into Covidence (Veritas Health Innovation, Melbourne, Australia) for screening. Title and abstract screening was conducted by one reviewer. A random sample (10%) of excluded records were checked to minimise screening errors (Cohen’s Kappa = 0.48). All full texts were independently screened for eligibility by two reviewers (Cohen’s Kappa = 0.60). If eligibility could not be determined based on an article, we searched for other publications reporting on that same study to obtain further information. Eligibility disagreements were resolved by discussion. After the original criteria were applied, the number of eligible articles (*n* = 166) was deemed too large for the review team’s resources. A second round of full-text screening was conducted with updated inclusion/exclusion criteria; studies had to report on randomised controlled trials, and pilot, feasibility, and small-scale trials (≤100 students at baseline) were excluded (Cohen’s Kappa = 0.98) (Table 2). Following screening, we conducted forward and backward citation tracking using Google Scholar, and searched through articles and their supplementary materials for peer-reviewed publications and other outputs relevant to studies eligible for inclusion.

## Data extraction

All data extraction was performed by one reviewer using a pre-piloted form. Articles not published in English (*n* = 2) were translated using DeepL Translator (available at www.deepl.com/translator). Details on staff training were extracted based on items in the Template for Intervention Description and Replication (TIDieR) checklist [17], a reporting guideline outlining the minimum set of items considered essential for intervention description and replication (e.g. use of theory, duration, mode of delivery). Where multiple training programmes were delivered within a study (e.g. in the form of content, dose, material etc. beyond local adaptation or personalisation), and outcome data were reported for each arm, data was sought and extracted for each arm. Information reported across study publications and outputs was pooled for data extraction. Where discrepancies were identified between study publications/outputs and data were mutually exclusive (e.g. training duration), data reported in the most recent outcome paper were selected. Where data differed but were mutually inclusive (e.g. BCTs), data were treated as cumulative and extracted as such.

Most studies (50/51; 98.0%) failed to report all TIDieR items about the staff training. Lead authors of included articles were contacted. They were requested to check and complete a partially filled TIDieR-based form, and to add any relevant study publications not listed. Authors were given three weeks to respond with a reminder email. Most authors responded (41/50; 82.0%) and 85.1% (39/41) provided additional information.

## Data coding, outcome classification and selection

### BCT coding

All training content extracted from peer-reviewed publications was compiled for coding, including any information about interventions delivered to staff in control groups. Other study outputs (e.g. websites) were not coded as access was variable between studies. Content was independently coded in duplicate by two reviewers for the presence and absence of BCTs using the BCT Taxonomy Version 1 (BCTTv1) [13]. Coders completed certified training in advance (available at www.bct-taxonomy.com). Only content that aimed to change staff behaviour within school hours and that specifically related to student activity behaviour was coded. Disagreements were resolved through discussion and by referring back to the BCTTv1 guidance (Cohen’s kappa = 0.70).

### Assessing and classifying fidelity outcome(s)

To account for differences in fidelity measurement and reporting across studies, we established a structured process (see Additional file 2) to assess, calculate, and classify fidelity outcomes as high (80–100%), medium (50–79%), or low (0–49%) fidelity [25]. All fidelity data was classified by one reviewer. A second reviewer checked all fidelity classifications (low, moderate, high); conflicts were resolved by discussion.

### Selecting activity behaviour outcomes

A single reviewer extracted one physical activity and one sedentary behaviour outcome per study. Where more than one of either outcome was reported, we applied a hierarchy (see Additional file 3) to focus on outcomes closest to the review’s exposure of interest. Activity behaviours measured during periods in which teachers were present for the greatest proportion of that time were prioritised as follows: i) teacher period, ii) school hours, iii) weekdays, and iv) whole of week. Where multiple physical activity outcomes within one of these periods were reported, outcomes were prioritised as follows: i) time spent in moderate-to-vigorous physical activity, ii) total physical activity, iii) vigorous physical activity, iv) moderate physical activity, and v) light physical activity, based on evidence of their respective associations with health outcomes [26, 27]. Where multiple sedentary behaviour outcomes within one of these periods were reported, we prioritised time spent in any sedentary behaviour above other outcomes (e.g. number of breaks in sedentary time). Where multiple follow-up measures were reported, outcomes measured closest to the end of the student-targeted intervention were extracted.

### Quality assessment

Quality assessment ratings of fidelity and activity behaviour outcomes were conducted independently by two reviewers using the Effective Public Health Practice Project (EPHPP) tool and dictionary [28, 29]. The EPHPP tool rates six individual domains; selection bias, study design, confounder bias, blinding, data collection methods, and withdrawals and drop out. Domain-specific ratings were used to calculate the global rating (‘strong’, ‘moderate’ or ‘weak’) according to the EPHPP dictionary. We piloted the EPHPP using a subsample of studies (*n* = 11 studies) to ensure consistency in interpretation of signalling questions between reviewers before starting the full set. Conflicts regarding global ratings were resolved through discussion (inter-rater agreement = 76.2 and 80.6% for fidelity and activity behaviour outcomes, respectively).

### Data synthesis

All statistical analyses were conducted using Stata (version 16.1). To assess the relative effectiveness of BCTs on fidelity, promise ratios were calculated as the frequency of a BCT appearing in a promising intervention (defined as high/moderate fidelity) divided by its frequency of appearance in a non-promising intervention (low fidelity) [30]. BCTs had to be identified in at least two interventions reporting eligible fidelity data to be assessed. Where BCTs were only identified in promising interventions, the promise ratio was calculated as the frequency of a BCT appearing in a promising intervention divided by one [30]. BCTs were considered promising if their calculated promise ratio was ≥2. Chi-square and Wilcoxon rank-sum tests were performed to assess differences in other training features (total training time, use of theory, session number, training period, number of BCTs) between moderate/high and low fidelity studies. The level of statistical significance and confidence were set at 5 and 95%, respectively. Results are reported in accordance with the Synthesis Without Meta-analysis guidelines [31].

### Meta-analysis

Intervention effects on physical activity and sedentary behaviour outcomes were analysed separately. Standardised mean differences (SMDs) were used to estimate effect sizes, and calculated based on the number, mean, and standard deviations (SDs) of treatment and control groups at baseline and follow-up. Additional file 4 outlines all formulae used to calculate SMDs and their standard errors (SEs) to perform random-effects meta-analyses. Where means and SDs were reported at a subgroup level (e.g. by sex), formulae outlined in the Cochrane handbook [32] were used to estimate outcomes at the unit of interest. Missing SDs were calculated using SEs, 95% confidence intervals (CIs), and t-distributions using formulae [32]. Where both SDs and means were missing, these were calculated using medians and interquartile ranges (IQRs) using Wan’s formulae [33, 34]. Studies that did not report on the mean and SD values of the same sample size at baseline and follow-up were excluded from analyses. Cohen thresholds were used to interpret SMDs as trivial (< 0.2), small (≥0.2 to < 0.5), moderate (≥0.5 to < 0.8), and large (≥0.8) [35]. Random-effects meta-regressions were performed to explore variations in effect estimates for outcomes as a function of BCTs, total number of BCTs, total training time, number of training sessions, and training period. In line with previous reviews [36], only BCTs unique to treatment groups and those identified in at least four interventions were included in analyses. Statistical heterogeneity was assessed using forest plots, the tau-squared (τ^2^) value and its 95% prediction interval [37]. Publication bias was assessed by visual inspection of funnel plots and Egger’s test.

## Results

### Overview of studies included

**Fig. 1:**
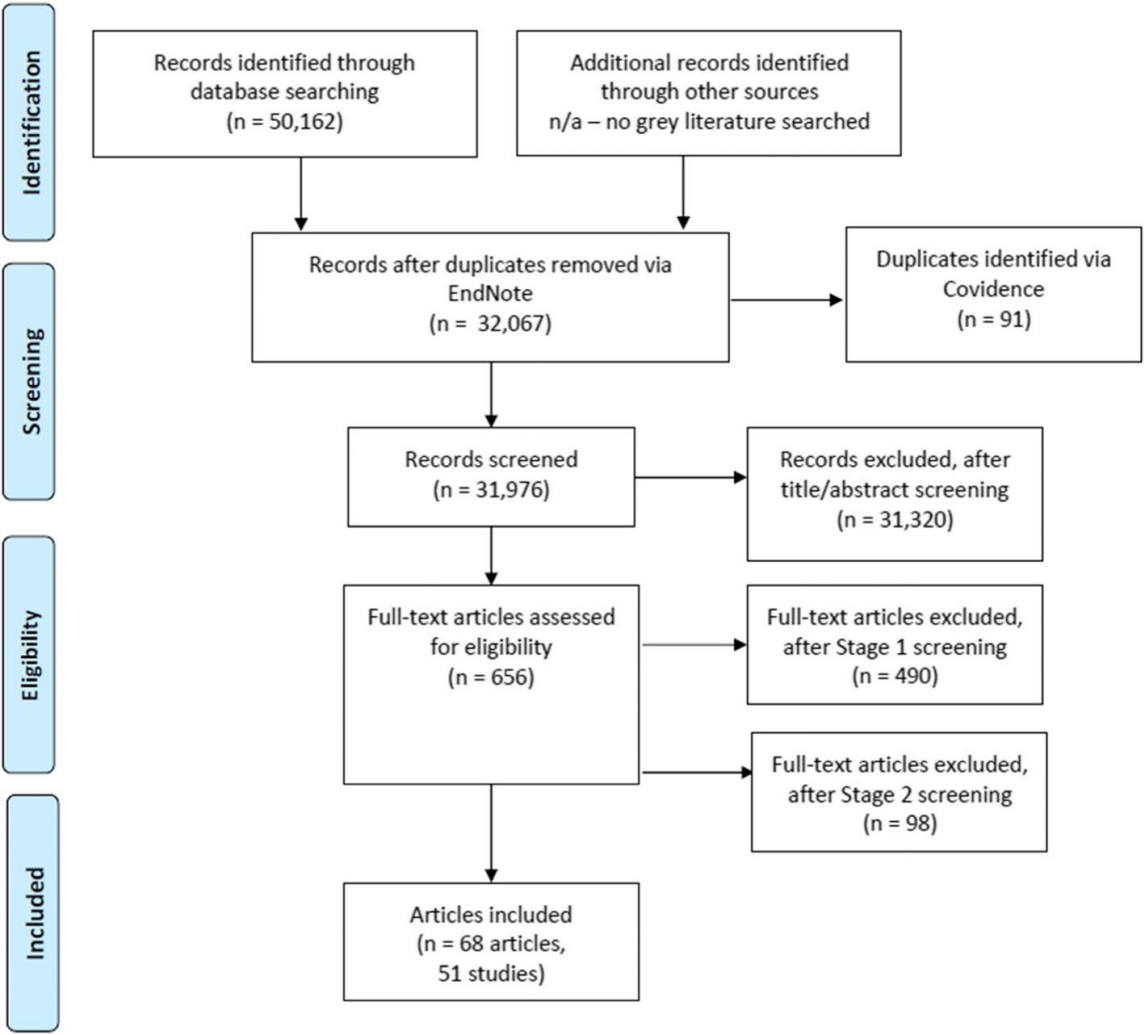
PRISMA flow diagram for study inclusion. n/a = not applicable

Figure 1 outlines the screening process, resulting in the inclusion of 51 individual studies. Further information about articles excluded during full-text screening is available in Additional file 5.

Studies originated from 19 countries, although 51% were from three countries (Australia: 19.6% [38–54], the United States: 15.7% [55–63] and the United Kingdom: 15.7% [64–75]). Most were conducted in primary school settings (*n* = 32, 62.8%). At baseline, the median number of schools and students per study was 14 (IQR: 9–24) and 779 (IQR: 361–1397), respectively. Fifty-three eligible training programmes were identified across 51 studies. Based on the percentage of studies with data reported, most programmes were delivered face-to-face (88.2%), in a group setting (60.5%), by research team members (65.3%) and underpinned by some theory or rationale (74.4%). The median training time was 7 hours (IQR: 2–14 hours). The median session count was 2 (IQR: 1–3). Full study details, including any theory or rationale used to inform training, are outlined in Additional file 6.

### Use of BCTs in training programmes (RQ1)

Thirty-seven out of 93 possible unique BCTs were identified across 53 training programmes (see Table 3). The mean number of BCTs identified per treatment group was 5.1 (SD = 3.2; range = 1–15). Two BCTs were identified in at least 50% of treatment groups; ‘Instruction on how to perform the behaviour’ (98.1%) and ‘Social support (unspecified)’ (50.9%). We also identified BCTs in two control staff training programmes [61, 76]; ‘Instruction on how to perform the behaviour’ was coded in each of these.

**Table 3:** Behaviour change techniques (BCTs) identified in treatment and control groups of training programmes (*n* = 53)

BCT	BCT label	Lead author of publication included in review and name of trial^**a**^																			
		Aadland ASK	Adab WAVES	Aittasalo KIDS OUT!	Anderson AFLY5	Belton Y-PATH	Bundy Sydney Playground Project	Chan A + FMS	Christiansen SPACE	Cohen SCORES	Drummy No trial name	Duncan the Healthy Homework Study	Dyrstad the Active School Study	Escriva-Boulley No trial name	Filho Fortaleça sua Saúde	Gray Choice, Control and Change	Ha No trial name	Ha SELF-FIT	Harrington Girls Active	Have No trial name	Hillman A + PACC
**4.1**	Instruction on how to perform the behaviour	☑	☑	☑	☑	☑	☑	☑	☑	☑	☑	☑	☑	☑	☑	☑	☑	☑	☑	☑	☑
**3.1**	Social support (unspecified)	☑											☑		☑	☑		☑	☑	☑	☑
**12.5**	Adding objects to the environment	☑			☑		☑			☑					☑			☑			
**1.1**	Goal setting (behaviour)				☑	☑		☑			☑		☑		☑		☑	☑			☑
**2.2**	Feedback on behaviour							☑		☑			☑	☑							☑
**6.1**	Demonstration of the behaviour																☑	☑			
**1.4**	Action planning													☑		☑			☑		
**8.1**	Behavioural practice/rehersal												☑	☑			☑	☑			
**1.2**	Problem solving													☑		☑	☑	☑	☑		
**2.3**	Self-monitoring of behaviour							☑										☑			
**3.2**	Social support (practical)												☑		☑		☑			☑	
**5.1**	Information about health consequences											☑			☑						
**12.1**	Restructuring the environment														☑						
**1.3**	Goal setting (outcome)		☑																		
**7.1**	Prompts/cues																				
**2.6**	Biofeedback																				
**10.2**	Material reward (behaviour)																		☑		☑
**10.3**	Non-specific reward																				
**13.1**	Identification of self as role model					☑															☑
**13.2**	Framing/reframing		☑				☑														
**1.5**	Review behaviour goal(s)													☑							
**1.6**	Discrepancy between current behavior and goal																	☑			
**1.7**	Review outcome goal(s)																		☑		
**2.1**	Monitoring of behaviour by others without feedback																			☑	
**6.3**	Information about others’ approval						☑														
**1.8**	Behavioural contract																				
**2.7**	Feedback on outcome(s) of behaviour																				
**4.4**	Behavioural experiments																				
**5.3**	Information about social and environmental consequences														☑						
**5.6**	Information about emotional consequences																				
**6.2**	Social comparison																				
**8.3**	Habit formation																				
**8.7**	Graded tasks																				
**9.1**	Credible source																				
**9.2**	Pros and cons						☑														
**10.4**	Social reward																				
**15.1**	Verbal persuasion about capability																				
	**Total number of BCTs identified per experimental arm**	3	3	1	3	3	5	4	1	3	2	2	6	6	8	4	6	9	6	4	6
	**Total number of BCTs identified per control arm**	0	0	0	0	0	0	0	0	0	0	0	0	1	0	0	0	0	0	0	0

BCT	BCT label	Lead author of publication included in review and name of trial^**a**^																			
		Hodges KIA	Hollis PA4E1	Janssen PLAYgrounds	Kelly COPE TEEN	Kennedy Resistance Training for Teens	Kien Bewegte Klasse	Kocken EF!	Köykkä Let’s Move It	Lonsdale AMPED	Lubans ATLAS	Martin Active Classrooms	McKay AC! BC!	Miller PLUNGE	Morris No trial name	Nader GROW HKC	Norris Virtual Traveller	O’Leary Project Spraoi	O’Neill Michigan Model for Health	Okely Girls in Sport	Riley EASY Minds
**4.1**	Instruction on how to perform the behaviour	☑	☑	☑	☑	☑	☑		☑	☑	☑	☑	☑	☑	☑	☑	☑	☑	☑	☑	☑
**3.1**	Social support (unspecified)		☑	☑				☑	☑	☑			☑	☑	☑		☑	☑	☑	☑	☑
**12.5**	Adding objects to the environment	☑	☑	☑		☑			☑	☑	☑		☑					☑			☑
**1.1**	Goal setting (behaviour)	☑				☑						☑					☑	☑			☑
**2.2**	Feedback on behaviour		☑	☑	☑	☑				☑	☑			☑							☑
**6.1**	Demonstration of the behaviour	☑			☑	☑	☑			☑	☑			☑	☑		☑	☑			☑
**1.4**	Action planning			☑		☑			☑	☑		☑	☑				☑	☑		☑	☑
**8.1**	Behavioural practice/rehersal		☑	☑	☑	☑			☑	☑				☑							☑
**1.2**	Problem solving								☑			☑								☑	☑
**2.3**	Self-monitoring of behaviour					☑			☑	☑		☑								☑	
**3.2**	Social support (practical)		☑															☑			
**5.1**	Information about health consequences								☑		☑						☑				
**12.1**	Restructuring the environment			☑					☑			☑									
**1.3**	Goal setting (outcome)		☑							☑			☑							☑	
**7.1**	Prompts/cues		☑						☑			☑									
**2.6**	Biofeedback				☑					☑								☑			
**10.2**	Material reward (behaviour)		☑																		
**10.3**	Non-specific reward									☑	☑										☑
**13.1**	Identification of self as role model			☑																	
**13.2**	Framing/reframing			☑																	
**1.5**	Review behaviour goal(s)																	☑			
**1.6**	Discrepancy between current behavior and goal									☑											
**1.7**	Review outcome goal(s)																			☑	
**2.1**	Monitoring of behaviour by others without feedback																				
**6.3**	Information about others’ approval								☑												
**1.8**	Behavioural contract																				
**2.7**	Feedback on outcome(s) of behaviour		☑																		
**4.4**	Behavioural experiments								☑												
**5.3**	Information about social and environmental consequences																				
**5.6**	Information about emotional consequences																☑				
**6.2**	Social comparison								☑												
**8.3**	Habit formation								☑												
**8.7**	Graded tasks													☑							
**9.1**	Credible source																			☑	
**9.2**	Pros and cons																				
**10.4**	Social reward								☑												
**15.1**	Verbal persuasion about capability																				
	**Total number of BCTs identified per experimental arm**	4	10	9	5	8	2	1	15	12	6	7	5	6	3	1	7	9	2	8	10
	**Total number of BCTs identified per control arm**	0	0	0	0	0	0	0	0	0	0	0	0	0	0	1	0	0	0	0	0

BCT	BCT label	Lead author of publication included in review and name of trial^**a**^													No of intervention studies where BCT coded as present (*n* = 53)	% of intervention studies where BCT coded as present
		Robertson FitQuest	Seibert No trial name	Smedegaard Move for Wellbeing in School	Sutherland No trial name	Tarp LCoMotion	Tymms Peer mentoring vs control	Tymms Participative learning vs control	van den Berg No trial name	Verloigne UP4FUN - The ENERGY Project	Vik UP4FUN	Wright 100 mile vs control	Wright Chalk vs control	Zhou SPE vs control		
**4.1**	Instruction on how to perform the behaviour	☑	☑	☑	☑	☑	☑	☑	☑	☑	☑	☑	☑	☑	52	98.1
**3.1**	Social support (unspecified)		☑	☑	☑						☑	☑	☑		27	50.9
**12.5**	Adding objects to the environment				☑	☑				☑	☑			☑	21	39.6
**1.1**	Goal setting (behaviour)				☑	☑				☑				☑	19	35.8
**2.2**	Feedback on behaviour				☑				☑					☑	16	30.2
**6.1**	Demonstration of the behaviour	☑							☑						15	28.3
**1.4**	Action planning				☑									☑	15	28.3
**8.1**	Behavioural practice/rehersal			☑	☑									☑	15	28.3
**1.2**	Problem solving				☑							☑			11	20.8
**2.3**	Self-monitoring of behaviour					☑									8	15.1
**3.2**	Social support (practical)		☑												7	13.2
**5.1**	Information about health consequences									☑	☑				7	13.2
**12.1**	Restructuring the environment									☑				☑	6	11.3
**1.3**	Goal setting (outcome)														5	9.4
**7.1**	Prompts/cues				☑						☑				5	9.4
**2.6**	Biofeedback													☑	4	7.5
**10.2**	Material reward (behaviour)				☑										4	7.5
**10.3**	Non-specific reward														3	5.7
**13.1**	Identification of self as role model														3	5.7
**13.2**	Framing/reframing														3	5.7
**1.5**	Review behaviour goal(s)														2	3.8
**1.6**	Discrepancy between current behavior and goal														2	3.8
**1.7**	Review outcome goal(s)														2	3.8
**2.1**	Monitoring of behaviour by others without feedback													☑	2	3.8
**6.3**	Information about others’ approval														2	3.8
**1.8**	Behavioural contract					☑									1	1.9
**2.7**	Feedback on outcome(s) of behaviour														1	1.9
**4.4**	Behavioural experiments														1	1.9
**5.3**	Information about social and environmental consequences														1	1.9
**5.6**	Information about emotional consequences														1	1.9
**6.2**	Social comparison														1	1.9
**8.3**	Habit formation														1	1.9
**8.7**	Graded tasks														1	1.9
**9.1**	Credible source														1	1.9
**9.2**	Pros and cons														1	1.9
**10.4**	Social reward														1	1.9
**15.1**	Verbal persuasion about capability				☑										1	1.9
	**Total number of BCTs identified per experimental arm**	2	3	3	11	5	1	1	3	5	5	3	2	9		
	**Total number of BCTs identified per control arm**	0	0	0	0	0	0	0	0	0	0	0	0	0		

Bold indicates BCT coded in both experimental and control arm of intervention; a indicates lead authors last name

^a^Name of arm cited where more than one training eligible for inclusion within a study

### Association with intervention fidelity (RQ2)

Thirty-five studies reported eligible fidelity data. Most (32/35; 91.4%) achieved a ‘weak’ overall quality assessment rating. Ten interventions (28.6%) were delivered with high fidelity, 18 with medium fidelity (51%) and seven with low fidelity (20%) (see Additional file 7 for domain ratings and fidelity classifications). Nineteen BCTs were associated with promising fidelity outcomes. The BCTs that held the highest promise ratio were ‘Adding objects to the environment’, ‘Feedback on behaviour’, ‘Demonstration of the behaviour’, ‘Behavioural practice/rehearsal’, and ‘Goal setting (behaviour)’. Eleven BCTs were unique to promising interventions (see Table 4).

**Table 4:** Behaviour change techniques (BCTs) associated with promising fidelity outcomes, in descending order of promise ratio (*n* = 35 studies)

BCT label^a^	Times BCT coded in a promising intervention (*N* = 28)	Times BCT coded in a non-promising intervention (*N* = 7)	Promise ratio
Adding objects to the environment	12	1	12.0
Feedback on behaviour	12	0	12.0
Demonstration of the behaviour	11	1	11.0
Behavioural practice/rehearsal	11	0	11.0
Goal setting (behaviour)	10	1	10.0
Action planning	7	1	7.0
Instruction on how to perform the behaviour	28	6	4.7
Social support (practical)	4	0	4.0
Social support (unspecified)	14	4	3.5
Information about health consequences	3	0	3.0
Non-specific reward	3	0	3.0
Problem solving	5	2	2.5
Self-monitoring of behaviour	5	2	2.5
Discrepancy between current behaviour and goal	2	0	2.0
Biofeedback	2	0	2.0
Prompts/cues	2	0	2.0
Material reward (behaviour)	2	0	2.0
Restructuring the environment	2	0	2.0
Identification of self as role model	2	0	2.0
Goal setting (outcome)	2	2	1.0
Framing/reframing	1	1	1.0

^a^BCTs coded in at least two interventions with a fidelity classification

Moderate/high fidelity studies were significantly more likely to include theory-informed and shorter training programmes than low fidelity studies (see Table 5). All other differences between training features and fidelity outcomes were non-significant.

**Table 5:** Training features associated with promising fidelity outcomes (*n* = 35 studies)

Training features	Moderate/high fidelity *N* = 28	Low fidelity *N* = 7	***P***-value^a^
Mean number of BCTs used (±SD)	5.2 (± 3.6)	3.4 (± 2.4)	0.19
Number of studies reporting any theory/rationale used (%)	17 (85.0)	2 (28.5)	**< 0.01**
Median hours of total training time (IQR)	11.6 (4.3–14.0)	3.9 (0.6–7.0)	0.22
Median number of training sessions (IQR)	2.0 (1.0–3.0)	2.0 (1.0–2)	0.85
Median training delivery period (months) (IQR)^b^	6.0 (1.8–12)	21.0 (15–24)	**0.02**

*N* Number of studies with available data, *IQR* Interquartile range

^a^Based on Chi-square and Wilcoxon rank-sum tests (bold: *p* < 0.05)

^b^Period over which training delivered if more than one session delivered

### Impact on student activity behaviour (RQ3)

Fifteen studies reported eligible physical activity data for inclusion in meta-analysis and 11 reported eligible sedentary behaviour data. Six studies (6/16 studies; 37.5%) achieved a ‘weak’ overall quality assessment rating, eight studies (50.0%) achieved a ‘moderate’ rating and two studies (12.5%) achieved a ‘strong’ rating (see Additional file 8 for domain ratings).

#### Physical activity

The median follow-up period for physical activity outcomes was 3 months (IQR: 6 weeks-8 months). The pooled effect size estimate was 0.44 (95% CI: 0.18, 0.71), indicating a significant positive intervention effect on students’ physical activity at follow-up (see Additional file 9). Heterogeneity was wide between studies (τ^2^ = 0.25; 95% prediction interval: −0.67, 1.56). Egger’s test indicated evidence of publication bias (*p* < 0.01) (see Additional file 9). Heterogeneity was largely driven by two studies [77, 78] that reported big effects and large adjusted SEs. When they were excluded from analyses, the pooled effect size estimate remained significant, 0.17 (95% CI: 0.02, 0.32), and Egger’s test did not indicate publication bias (*p* > 0.05) (see Additional file 9).

Meta-regressions were performed between BCTs eligible for analysis (*n* = 9), total number of BCTs, total training time, number of training sessions, and training period, and changes in physical activity outcomes from baseline to follow-up (Table 6). We found significant associations for the BCTs ‘Action planning’ and ‘Feedback on behaviour’, and total number of BCTs used (see Table 6). No other significant associations were identified.

**Table 6:** Meta-regression showing univariate effects of training features on physical activity outcomes (*n* = 15 studies)

Training features		β	SE	95% CI	***P***
Behaviour Change Techniques^a^					
1.1	Goal setting (behaviour)	0.29	0.44	−0.67, 1.25	0.53
1.2	Problem solving	−0.04	0.51	−1.14, 1.06	0.94
1.4	Action planning	1.40	0.32	0.70, 2.10	**< 0.01**
2.2	Feedback on behaviour	1.19	0.38	0.36, 2.02	**0.01**
3.1	Social support (unspecified)	0.24	0.45	−0.74, 1.22	0.61
6.1	Demonstration of the behaviour	−0.59	0.45	−1.55, 0.38	0.21
8.1	Behavioural practice/rehearsal	0.82	0.40	−0.03, 1.68	0.06
12.5	Adding objects to the environment	0.64	0.41	−0.26, 1.53	0.15
Total number of BCTs used		0.18	0.06	0.05, 0.31	**0.01**
Total training time (> 1 day)		0.16	0.53	−1.01, 1.32	0.78
Total number of training sessions		0.63	0.45	−0.36, 1.62	0.19
Period training delivered over (months)		0.05	0.09	−0.16, 0.26	0.61

Bold: *p* < 0.05

*β* Effect size estimate, *SE* Standard error, *CI* Confidence interval

^a^‘4.1 Instruction on how to perform the behaviour’ not analysed due to collinearity

#### Sedentary behaviour

The median follow-up period for sedentary behaviour outcomes was 4 months (IQR: 6 weeks-10 months). The pooled effect size estimate was 0.06 (95% CI: − 0.40, 0.53), indicating no effect on students’ sedentary behaviour at follow-up (see Additional file 10). Heterogeneity was wide between studies (τ^2^ = 0.59; 95% prediction interval: − 0.20, 0.36). Inspection of funnel plot and Egger’s test did not indicate publication bias (*p* > 0.05; see Additional file 10). Meta-regressions between training features and changes in sedentary behaviour outcomes from baseline to follow-up showed no significant associations (see Table 7).

**Table 7:** Meta-regression showing univariate effects of training features on sedentary behaviour outcomes (*n* = 11 studies)

Training features		β	SE	95% CI	***P***
Behaviour Change Techniques^a^					
1.1	Goal setting (behaviour)	−0.49	0.49	−1.60, 0.62	0.35
3.1	Social support (unspecified)	−0.73	0.46	−1.76, 0.30	0.15
8.1	Behavioural practice/rehearsal	−0.34	0.52	−1.52, 0.84	0.53
12.5	Adding objects to the environment	−0.41	0.50	−1.54, 0.72	0.43
Total number of BCTs used		−0.08	0.09	−0.28, 0.11	0.37
Total training time (> 1 day)		−0.09	0.53	−1.29, 1.10	0.87
Total number of training sessions		−0.52	0.48	−1.61, 0.56	0.31
Period training delivered over (months)		0.00	0.03	−0.07, 0.07	0.95

*β* Effect size estimate, *SE* Standard error, *CI* Confidence interval

^a^‘4.1 Instruction on how to perform the behaviour’ not analysed due to collinearity

## Discussion

This is the first systematic review to identify BCTs used in staff training programmes delivered within school-based intervention studies aimed at changing student activity behaviour. We identified 53 eligible training programmes and found evidence that 37 unique BCTs have been used to change teacher behaviour. We found evidence that 19 BCTs are positively associated with promising fidelity outcomes, and that moderate/high fidelity studies are more likely to include theory-based and shorter training programmes (≤ 6 months) than low fidelity studies. We also found training programmes that use more BCTs and those that use ‘Action planning’ and ‘Feedback on the behaviour’ are associated with significant changes to students’ device-measured physical activity. We found no associations between training features and sedentary behaviour outcomes.

The mean number of BCTs identified per training programme suggests that few teacher-targeted BCTs have been used within school-based teacher-led activity behaviour interventions. The only frequently identified BCTs were ‘Instruction on how to perform the behaviour’ and ‘Social support (unspecified)’. The literature suggests that the use of these BCTs alone is unlikely to achieve or sustain professional change [14]. Certain well-evidenced BCTs were absent across studies. For example, a large body of research has highlighted the importance of providing teachers with tools to notice change in their students to promote professional change (e.g. [79]). Yet we identified ‘Feedback on outcome of the behaviour’ in just one training programme [50].

Many study authors reported that the training was underpinned by some rationale or theory, but the theory underpinning the intervention aimed at the student was often conflated with the theory underpinning the staff training (e.g. [38, 80]). In such instances, it was often unclear how the theory was used to inform the training. Few authors drew on relevant teacher professional development literature or theory to inform the design of programmes; this may help to explain the limited number of evidence-based BCTs identified across training programmes. Further, many authors provided no information (e.g. [59, 62]) or confirmed that the training was not informed by any theory or rationale (e.g. [63, 64, 81, 82]).

We found evidence to support an association between 19 BCTs and teacher fidelity. The most promising BCTs we identified were ‘Feedback on behaviour’, ‘Demonstration of the behaviour’, ‘Behavioural practice/rehearsal’, and ‘Goal setting (behaviour)’. Their use in future training programmes is supported by reviews examining causal components of effective teacher professional development for other school subjects (e.g. [14, 15, 83]). ‘Adding objects to the environment’ is less frequently cited within the literature. The objects provided (e.g. maths bingo tiles, sports equipment, signage, standing desks [53, 59, 84–86]) may have prompted teachers to implement the intervention on an ongoing basis. Further research is needed to determine how teaching resources and their placement within school settings may promote implementation. Consistent with findings from recent reviews (e.g. [15, 16, 87]), we found that training quality (i.e. theory-based training and use of evidence-based BCTs) rather than a longer training duration was associated with intervention fidelity.

We also found evidence to support the use of more BCTs and the use of ‘Action Planning’ and ‘Feedback on behaviour’ in staff training to increase students’ physical activity. Conversely, we found no evidence to support an association between training features and sedentary behaviour outcomes. These findings may be explained by the small number of studies that observed significant intervention effects, that measured sedentary behaviour during teacher periods and that specifically targeted students’ sedentary behaviour. Interventions must not just be effective but also feasible for teachers to implement and sustain within their workload. Recent research has found that participants often receive more implementation support in pilot interventions than those participating in larger-scale trials of the same or similar interventions [88]. Hence, it is also possible that the interventions were not feasible for teachers to deliver. Finally, quality teaching indicators (e.g. [89]) have yet to be identified within the context of student physical activity and sedentary behaviour. The techniques teachers were requested to implement, even when delivered with fidelity, may have been ineffective in changing student’s activity behaviour.

## Strengths and limitations of the review

We employed a comprehensive search to identify and extract data about staff training by using a standardised reporting checklist, searching across study publications and outputs, and contacting authors to overcome limitations of existing reviews that observed poor reporting practices [12]. We achieved a high response rate from study authors and few changes were made to our partially completed forms, suggesting that data about the teacher training programmes was reliably extracted. We overcame limitations associated with recent teacher professional development reviews for other subjects (e.g. [14, 15, 90]), by exploring training effects on both professional practice and student outcomes [90], and by examining data from largely pre-registered [14, 90] and medium-to-large-scale studies [15].

Eligible studies and outputs may have been missed. To reduce the likelihood of missing outputs, all authors were contacted and requested to add study publications not listed. Due to resource limitations, all data extraction was conducted by a single reviewer, which may have resulted in extraction errors. Further, while a structured process was used to classify fidelity data into outcomes, this was conducted by a single reviewer and solely checked by a second. Studies conducted in low and middle-income countries and not published in English are likely disproportionately excluded due to eligibility criteria and databases used. Researchers and practitioners should be cautious about applying the findings to settings and populations underrepresented in this review. Where authors reported fidelity outcomes at multiple time points (e.g. [45, 54, 56, 77]), we selected outcomes measured closest to the training start time. BCTs identified may hence promote short-term fidelity, and should be used alongside evidence-based BCTs that promote sustained professional change (e.g. ‘Habit formation’ [14]). Finally, effective training features that are beyond the scope of the BCTTv1 and TIDieR checklist may exist but were not explored in the current review.

## Limitations of the underlying evidence

Most of the limitations associated with our findings relate to the quality of the evidence we reviewed. Consistent with previous reviews [12, 91, 92], we observed poor reporting on staff training across studies. Consequently, it is difficult to discern whether the BCTs identified reflect what was delivered in practice. In line with previous reviews [93], fidelity measures used across studies were methodologically weak. Many studies did not report on fidelity to all intervention components or at the individual level. The BCTs identified may therefore overestimate the extent to which their use can promote overall fidelity, and warrant testing across intervention components, teacher populations and school climates. We sought to include all quantitative fidelity data in our analyses to make the best use of available data [94], but had to exclude 30% of studies as outcomes were reported in isolation of any identifiable target with which we could interpret the data (e.g. [78, 95]). This reduced the number of studies on which we could base our findings.

## Implications

In line with existing guidance [7], we recommend that researchers engage with discipline-specific experts and literature when designing and evaluating all intervention components. In order for the field to progress, complete and consistent reporting is needed to determine what interventions have been delivered to the various actors within activity behaviour intervention studies. Consistent and effective implementation of reporting guidelines are important for this, but at the time of paper submission, we found that out of 33 journals that published articles included here just one explicitly requested submission of reporting checklists for all intervention components. We have therefore written to journal editors to update their submission policies to require study authors to submit relevant reporting checklists (e.g. [17, 96]) that describe each of the interventions being implemented and/or assessed [97]. We also advise that study authors use machine-readable tools (e.g. [98]) from the protocol stage to avoid inconsistent reporting within and across study outputs. Finally, valid, reliable and acceptable fidelity measures are needed to determine how school-based interventions are being implemented in practice. Progress is needed to understand the level of support teachers require for effective implementation, components teachers are most likely to deliver, and practices causally related to student activity behaviour change.

## Conclusion

This review advances our understanding of how school-based interventions have been implemented, and identifies specific, replicable techniques that can be incorporated into future programmes to promote intervention fidelity and increase student physical activity. Our findings suggest training programmes should be informed by relevant theory and literature and include a combination of BCTs that provide teachers with i) a demonstration of the desired behaviour, ii) an opportunity to practice the behaviour, iii) feedback on their performance of the behaviour, iv) a behavioural goal (self-defined or otherwise) and v) objects that facilitate and cue performance of the behaviour. Our findings also suggest teachers should be prompted to make a detailed action plan regarding their performance of the behaviour. We encourage researchers to incorporate BCTs that have been shown to promote sustained professional change for other school subjects, so that their effectiveness can be assessed within the context of physical activity and sedentary behaviour. Changes to reporting practices in the field will enable researchers in time to determine BCT combinations and features (e.g. frequency, sequence) that best predict desired outcomes for defined teacher and student populations.

## Supplementary Information

**Additional file 1.** Search terms and records identified.

**Additional file 2.** Structured process to classify fidelity outcomes.

**Additional file 3.** Hierarchies used to select activity behaviour outcomes.

**Additional file 4.** Formulae used for meta-analyses of physical activity and sedentary behaviour outcomes.

**Additional file 5.** Publications excluded with reasons at stages 1 and 2 of full-text screening.

**Additional file 6. **Table of descriptive characteristics of studies (*n* = 51) included in systematic review of school-based activity behaviour interventions.

**Additional file 7.** Quality assessment ratings and classification results for fidelity outcomes.

**Additional file 8.** Quality assessment ratings for activity behaviour outcomes (physical activity and sedentary behaviour combined).

**Additional file 9.** Forest and funnel plots for physical activity outcomes.

**Additional file 10.** Forest plots and funnel plots for sedentary behaviour outcomes.

## Data Availability

A summary of reviewed studies and their outputs is available in Additional file 6.

## Abbreviations

MRC: Medical Research Council
BCT: Behaviour Change Technique
SMD: Standardised Mean Difference
TIDieR: Template for Intervention Description and Replication
EPHPP: Effective Public Health Practice Project
SD: Standard deviation
SE: Standard error
CI: Confidence interval
IQR: Interquartile range
