# Supplementary material for: Features of effective staff training programmes within school-based interventions targeting student activity behaviour: a systematic review and meta-analysis
Source: Int J Behav Nutr Phys Act. 2022 Sep 24;19:125. doi: 10.1186/s12966-022-01361-6 (PMC9509574; doi:10.1186/s12966-022-01361-6)
Supplement: Supplementary file 1 — Additional file 1. Search terms and records identified. [file 12966_2022_1361_MOESM1_ESM.docx]

Additional File 1. Search terms and records identified

| **Database** | **Number of identified records** |
| --- | --- |
| MEDLINE (via Ovid) | 7,172 |
| EMBASE (via Ovid) | 10,909 |
| Applied Social Sciences Index and Abstracts (ASSIA) | 573 |
| Scopus | 3,633 |
| Education Resources Information Center (ERIC) | 3,043 |
| Web of Science Core Collection | 22,361 |
| SPORTDiscus | 2,471 |
| **Total** | **50,162** |
|  |  |

All searches were performed on May 15^th^ 2020.

**Search terms and records identified (MEDLINE (via Ovid))**

| **Search** | **Terms** | **Records** |
| --- | --- | --- |
|  | (child* or boy* or girl* or kid* or mid-adolescen* or adolescen* or youth* or (young adj (people or person)) or teen* or juvenile or student* or school* or pupil*).ti,ab. | 2506455 |
|  | child/ | 1673861 |
|  | adolescent/ | 2009709 |
|  | students/ | 57723 |
|  | 1 or 2 or 3 or 4 | 4164657 |
|  | ("physical* adj activ*" or sport* or cycle or cycling or bicycling or bicycle* or walk* or "physical education" or "physical training" or exercis* or (energy adj expenditure) or danc* or inactiv* or "physical fitness" or lifestyle or "active lesson*" or "aerobic fitness" or sedentar* or sit*).ti,ab. | 3135158 |
|  | motor activity/ | 96231 |
|  | sports/ | 30073 |
|  | exercise/ | 107801 |
|  | sedentary behavior/ | 9091 |
|  | physical exertion/ | 56223 |
|  | "physical education and training"/ | 13414 |
|  | 6 or 7 or 8 or 9 or 10 or 11 or 12 | 3265709 |
|  | ("control* trial" or randomi* or randomly or trial* or evaluation or effect* or control* or cluster or intervention or pilot or feasibility or prospective* or observation* or longitudinal or follow-up* or followup* or (follow* adj up*) or cohort* or "process evaluation").ti,ab. | 11948838 |
|  | randomized controlled trial/ | 505469 |
|  | longitudinal studies/ | 133769 |
|  | prospective studies/ | 537453 |
|  | follow-up studies/ | 639934 |
|  | cohort studies/ | 260552 |
|  | 14 or 15 or 16 or 17 or 18 or 19 | 12250964 |
|  | ("case study" or "case report" or "abstract report" or letter).ti,ab. | 458585 |
|  | letter/ | 1076957 |
|  | historical article/ | 358050 |
|  | case report/ | 2096342 |
|  | 22 or 23 or 24 | 3310529 |
|  | 20 not 25 | 11696728 |
|  | (acceleromet* or accelerometer-assessed or "counts per minute" or CPM or actigraph* or "heart rate" or pedomet* or "objective* measur*" or "device measur*" or "activity monitor" or MVPA or LIPA or ((process or program*) adj (evaluation* or monitoring)) or (qualitative adj (component* or aspect* or approach*)) or participant observation* or fidelity or adher* or monitor* or implement* or integrat* or adopt* or uptake or dosage or reach or dose or quality or sustainabil* or implement*).ti,ab. | 4444538 |
|  | monitoring, ambulatory/ | 8061 |
|  | actigraphy/ | 3563 |
|  | Implementation Science/ | 335 |
|  | Process Assessment, Health Care/ | 4625 |
|  | Program Evaluation/ | 62399 |
|  | "Outcome and Process Assessment, Health Care"/ | 26867 |
|  | 27 or 28 or 29 or 30 or 31 or 32 or 33 | 4496078 |
|  | (school* or educat* or teach* or academi*).ti,ab. | 994685 |
|  | 5 and 13 and 26 and 34 and 35 | 17472 |
|  | limit 36 to yr="2015 -Current" | **7,172** |

**Search terms and records identified (EMBASE via Ovid)**

| **Search** | **Terms** | **Records** |
| --- | --- | --- |
|  | (child* or boy* or girl* or kid* or mid-adolescen* or adolescen* or youth* or (young adj (people or person)) or teen* or juvenile or student* or school* or pupil*).ti,ab. | 2527454 |
|  | child/ | 1296375 |
|  | adolescent/ | 1158670 |
|  | student/ | 101733 |
|  | 1 or 2 or 3 or 4 | 3304599 |
|  | ("physical* adj activ*" or sport* or cycle or cycling or bicycling or bicycle* or walk* or "physical education" or "physical training" or exercis* or (energy adj expenditure) or danc* or inactiv* or "physical fitness" or lifestyle or "active lesson*" or "aerobic fitness" or sedentar* or sit*).ti,ab. | 3193475 |
|  | motor activity/ | 33461 |
|  | sport/ | 40511 |
|  | exercise/ | 226290 |
|  | sedentary lifestyle/ | 13782 |
|  | physical education/ | 8268 |
|  | 6 or 7 or 8 or 9 or 10 or 11 | 3277327 |
|  | ("control* trial" or randomi* or randomly or trial* or evaluation or effect* or control* or cluster or intervention or pilot or feasibility or prospective* or observation* or longitudinal or follow-up* or followup* or (follow* adj up*) or cohort* or "process evaluation").ti,ab. | 12554961 |
|  | randomized controlled trial/ | 556538 |
|  | longitudinal study/ | 132011 |
|  | prospective study/ | 577754 |
|  | follow up/ | 1463848 |
|  | cohort analysis/ | 568195 |
|  | 13 or 14 or 15 or 16 or 17 or 18 | 12860162 |
|  | ("case study" or "case report" or "abstract report" or letter).ti,ab. | 484817 |
|  | letter/ | 783910 |
|  | 20 or 21 | 1231470 |
|  | 19 not 22 | 12558623 |
|  | (acceleromet* or accelerometer-assessed or "counts per minute" or CPM or actigraph* or "heart rate" or pedomet* or "objective* measur*" or "device measur*" or "activity monitor" or MVPA or LIPA or ((process or program*) adj (evaluation* or monitoring)) or (qualitative adj (component* or aspect* or approach*)) or participant observation* or fidelity or adher* or monitor* or implement* or integrat* or adopt* or uptake or dosage or reach or dose or quality or sustainabil* or implement*).ti,ab. | 5068065 |
|  | ambulatory monitoring/ | 9149 |
|  | actimetry/ | 8920 |
|  | implementation science/ | 974 |
|  | program evaluation/ | 14508 |
|  | 24 or 25 or 26 or 27 or 28 | 5076977 |
|  | (school* or educat* or teach* or academi*).ti,ab. | 1148009 |
|  | 5 and 12 and 23 and 29 and 30 | 23441 |
|  | limit 31 to yr="2015 -Current" | **10,909** |

**Search terms and records identified (Applied Social Sciences Index and Abstracts (ASSIA))**

| **Search** | **Terms** | **Records** |
| --- | --- | --- |
|  | ((ab((child* OR boy* OR girl* OR kids OR mid-adolescen* OR adolescen* OR youth* OR (young adj (people OR person)) OR teen* OR juvenile OR student* OR school* OR pupil*)) OR ti((child* OR boy* OR girl* OR kids OR mid-adolescen* OR adolescen* OR youth* OR (young adj (people OR person)) OR teen* OR juvenile OR student* OR school* OR pupil*)))  OR MAINSUBJECT.EXACT("Children")  OR MAINSUBJECT.EXACT("Adolescents")  OR MAINSUBJECT.EXACT("Students")) | 356,444* |
|  | AND |  |
|  | ((ab(("physical* adj activ*" or sport* or cycle or cycling or bicycling or bicycle* or walk* or "physical education" or "physical training" or exercis* or (energy adj expenditure) or danc* or inactiv* or "physical fitness" or lifestyle or "active lesson*" or "aerobic fitness" or sedentar* or sit*)) OR ti(("physical* adj activ*" or sport* or cycle or cycling or bicycling or bicycle* or walk* or "physical education" or "physical training" or exercis* or (energy adj expenditure) or danc* or inactiv* or "physical fitness" or lifestyle or "active lesson*" or "aerobic fitness" or sedentar* or sit*)))  OR MAINSUBJECT.EXACT("Motor activity")  OR MAINSUBJECT.EXACT("Sports")  OR MAINSUBJECT.EXACT("Exercise")  OR MAINSUBJECT.EXACT("Sedentary")  OR MAINSUBJECT.EXACT("Sedentary people")  OR MAINSUBJECT.EXACT("Physical education")) | 70,805* |
|  | AND |  |
|  | ((ab(("control* trial" or randomi* or randomly or trial* or evaluation or effect* or control* or cluster or intervention or pilot or feasibility or prospective* or observation* or longitudinal or follow-up* or followup* or (follow* adj up*) or cohort* or "process evaluation")) OR ti(("control* trial" or randomi* or randomly or trial* or evaluation or effect* or control* or cluster or intervention or pilot or feasibility or prospective* or observation* or longitudinal or follow-up* or followup* or (follow* adj up*) or cohort* or "process evaluation")))  OR MAINSUBJECT.EXACT("Randomized controlled trials")  OR MAINSUBJECT.EXACT("Clinical trials")  OR MAINSUBJECT.EXACT("Longitudinal studies")  OR MAINSUBJECT.EXACT("Prospective studies")  OR MAINSUBJECT.EXACT("Followup studies")  OR MAINSUBJECT.EXACT("Cohort analysis")  OR MAINSUBJECT.EXACT("Clinical trials")) | 511,361* |
|  | ((ab(("case study" OR "case report" OR "abstract report" OR letter)) OR ti(("case study" OR "case report" OR "abstract report" OR letter))  OR MAINSUBJECT.EXACT("Letters") OR MAINSUBJECT.EXACT("Case records"))) | 27,449* |
|  | S3 NOT S4 | 500,883* |
|  | ((ab((acceleromet* or accelerometer-assessed or "counts per minute" or CPM or actigraph* or "heart rate" or pedomet* or "objective* measur*" or "device measur*" or "activity monitor" or MVPA or LIPA or ((process or program*) adj (evaluation* or monitoring)) or (qualitative adj (component* aspect* or approach*)) or participant observation* or fidelity or adher* or monitor* or implement* or integrat* or adopt* or uptake or dosage or reach or dose or quality or sustainabil* or implement*)) OR ti((acceleromet* or accelerometer-assessed or "counts per minute" or CPM or actigraph* or "heart rate" or pedomet* or "objective* measur*" or "device measur*" or "activity monitor" or MVPA or LIPA or ((process or program*) adj (evaluation* or monitoring)) or (qualitative adj (component* or aspect* or approach*)) or participant observation* or fidelity or adher* or monitor* or implement* or integrat* or adopt* or uptake or dosage or reach or dose or quality or sustainabil* or implement*)) OR MAINSUBJECT.EXACT("monitoring") OR MAINSUBJECT.EXACT("actigraphy"))) | 241,298* |
|  | ti(school* OR educat* OR teach* OR academi*) OR ab(school* OR educat* OR teach* OR academi*) | 218,018* |
|  | S1 AND S2 AND S5 AND S6 AND S7 | 2,015° |
|  | S1 AND S2 AND S5 AND S6 AND S7 Limits applied | **573** |

**Search terms and records identified (Scopus)**

| **Search** | **Terms** | **Records** |
| --- | --- | --- |
|  | ( TITLE-ABS ( child* OR boy* OR girl* OR kids OR mid-adolescen* OR adolescen* OR youth* OR ( young W/1 ( people OR person ) ) OR teen* OR juvenile OR student* OR school* OR pupil* ) ) |  |
|  | ( TITLE-ABS ("physical* W/1 activ*" or sport* or cycle or cycling or bicycling or bicycle* or walk* or "physical education" or "physical training" or exercis* or (energy W/1 expenditure) or danc* or inactiv* or "physical fitness" or lifestyle or "active lesson*" or "aerobic fitness" or sedentar* or sit*)) |  |
|  | ( TITLE-ABS ("control* trial" or randomi* or randomly or trial* or evaluation or effect* or control* or cluster or intervention or pilot or feasibility or prospective* or observation* or longitudinal or follow-up* or followup* or (follow* W/1 up*) or cohort* or "process evaluation")) AND NOT ( TITLE-ABS ( "case study" OR "case report" OR "abstract report" OR letter ) ) |  |
|  | ( TITLE-ABS ( acceleromet* OR accelerometer-assessed OR "counts per minute" OR cpm OR actigraph* OR "heart rate" OR pedomet* OR "objective* measur*" OR "device measur*" OR "activity monitor" OR mvpa OR lipa OR ( ( process OR program* ) W/1 ( evaluation* OR monitoring ) ) OR ( qualitative W/1 ( component* OR aspect* OR approach* ) ) OR participant AND observation* OR fidelity OR adher* OR monitor* OR implement* OR integrat* OR adopt* OR uptake OR dosage OR reach OR dose OR quality OR sustainabil* OR implement* ) ) |  |
|  | ( TITLE-ABS (school* or educat* or teach* or academi*)) |  |
|  | #1 AND #2 AND #3 AND #4 AND #5 () |  |
|  | #1 AND #2 AND #3 AND #4 AND #5 Limits applied | **3,633** |

**Search terms and records identified (Education Resources Information Center (ERIC))**

| **Search** | **Terms** | **Records** |
| --- | --- | --- |
|  | TI ( (child* or boy* or girl* or kids or mid-adolescen* or adolescen* or youth* or (young adj (people or person)) or teen* or juvenile or student* or school* or pupil*) ) OR AB ( (child* or boy* or girl* or kids or mid-adolescen* or adolescen* or youth* or (young adj (people or person)) or teen* or juvenile or student* or school* or pupil*) ) | 1,132,011 |
|  | DE "Children" | 46,245 |
|  | DE "Adolescents" | 50,656 |
|  | DE "Students" | 4,948 |
|  | S1 OR S2 OR S3 OR S4 | 1,137,336 |
|  | TI (("physical* adj activ*" or sport* or cycle or cycling or bicycling or bicycle* or walk* or "physical education" or "physical training" or exercis* or (energy adj expenditure) or danc* or inactiv* or "physical fitness" or lifestyle or "active lesson*" or "aerobic fitness" or sedentar* or sit*)) OR AB (("physical* adj activ*" or sport* or cycle or cycling or bicycling or bicycle* or walk* or "physical education" or "physical training" or exercis* or (energy adj expenditure) or danc* or inactiv* or "physical fitness" or lifestyle or "active lesson*" or "aerobic fitness" or sedentar* or sit*)) | 172,311 |
|  | DE "Exercise" | 2,367 |
|  | DE "Physical Fitness" OR DE "Health Related Fitness" | 4,239 |
|  | DE "Physical Education" OR DE "Adapted Physical Education" OR DE "Movement Education" | 13,253 |
|  | S6 OR S7 OR S8 OR S9 | 175,863 |
|  | TI (("control* trial" or randomi* or randomly or trial* or evaluation or effect* or control* or cluster or intervention or pilot or feasibility or prospective* or observation* or longitudinal or follow-up* or followup* or (follow* adj up*) or cohort* or "process evaluation")) OR AB (("control* trial" or randomi* or randomly or trial* or evaluation or effect* or control* or cluster or intervention or pilot or feasibility or prospective* or observation* or longitudinal or follow-up* or followup* or (follow* adj up*) or cohort* or "process evaluation")) | 639,818 |
|  | DE "Randomized Controlled Trials" | 1,527 |
|  | DE "Longitudinal Studies" OR DE "Followup Studies" | 31,439 |
|  | S11 OR S12 OR S13 | 645,136 |
|  | TI ( ("case study" or "case report" or "abstract report" or letter) ) OR AB ( ("case study" or "case report" or "abstract report" or letter) ) | 66,091 |
|  | DE "Letters (Correspondence)" | 1,680 |
|  | S15 OR S16 | 66,500 |
|  | S14 NOT S17 | 614,912 |
|  | TI ( (acceleromet* or accelerometer-assessed or "counts per minute" or CPM or actigraph* or "heart rate" or pedomet* or "objective* measur*" or "device measur*" or "activity monitor" or MVPA or LIPA or ((process or program*) adj (evaluation* or monitoring)) or (qualitative adj (component* or aspect* or approach*)) or participant observation* or fidelity or adher* or monitor* or implement* or integrat* or adopt* or uptake or dosage or reach or dose or quality or sustainabil* or implement*) ) OR AB ( (acceleromet* or accelerometer-assessed or "counts per minute" or CPM or actigraph* or "heart rate" or pedomet* or "objective* measur*" or "device measur*" or "activity monitor" or MVPA or LIPA or ((process or program*) adj (evaluation* or monitoring)) or (qualitative adj (component* or aspect* or approach*)) or participant observation* or fidelity or adher* or monitor* or implement* or integrat* or adopt* or uptake or dosage or reach or dose or quality or sustainabil* or implement*) ) | 368,716 |
|  | DE "Program Evaluation" OR DE "Program Implementation" | 75,528 |
|  | S19 OR S20 | 406,269 |
|  | TI ( school* or educat* or teach* or academi* ) OR AB ( school* or educat* or teach* or academi* ) | 1,153,012 |
|  | S5 AND S10 AND S18 AND S21 AND S22 | **3,043** |

**Search terms and records identified (Web of Science)**

| **Search** | **Terms** | **Records** |
| --- | --- | --- |
|  | TS=(child* or boy* or girl* or kids or mid-adolescen* or adolescen* or youth* or (young NEAR/1 (people or person)) or teen* or juvenile or student* or school* or pupil*) |  |
|  | TS=("physical* NEAR/1 activ*" or sport* or cycle or cycling or bicycling or bicycle* or walk* or "physical education" or "physical training" or exercis* or (energy NEAR/1 expenditure) or danc* or inactiv* or "physical fitness" or lifestyle or "active lesson*" or "aerobic fitness" or sedentar* or sit*) |  |
|  | TS=("control* trial" or randomi* or randomly or trial* or evaluation or effect* or control* or cluster or intervention or pilot or feasibility or prospective* or observation* or longitudinal or follow-up* or followup* or (follow* NEAR/1 up) or cohort* or "process evaluation") |  |
|  | TS=("case study" or "case report" or "abstract report" or letter) |  |
|  | #3 NOT #4 |  |
|  | TS=( acceleromet* OR accelerometer-assessed OR "counts per minute" OR cpm OR actigraph* OR "heart rate" OR pedomet* OR "objective* measur*" OR "device measur*" OR "activity monitor" OR mvpa OR lipa OR ( ( process OR program* ) NEAR/1 ( evaluation* OR monitoring ) ) OR ( qualitative NEAR/1 ( component* OR aspect* OR approach* ) ) OR participant AND observation* OR fidelity OR adher* OR monitor* OR implement* OR integrat* OR adopt* OR uptake OR dosage OR reach OR dose OR quality OR sustainabil* OR implement*) |  |
|  | TS=(school* or educat* or teach* or academi*) |  |
|  | #1 AND #2 AND #5 AND #6 AND #7 (57,564) |  |
|  | #1 AND #2 AND #5 AND #6 AND #7 limits applied (); Web of Science Core Collection & 2015-2020 | **22,361** |

**Search terms and records identified (SPORTDiscus)**

| **Search** | **Terms** | **Records** |
| --- | --- | --- |
|  | TI ( (child* or boy* or girl* or kids or mid-adolescen* or adolescen* or youth* or (young adj (people or person)) or teen* or juvenile or student* or school* or pupil*) ) OR AB ( (child* or boy* or girl* or kids or mid-adolescen* or adolescen* or youth* or (young adj (people or person)) or teen* or juvenile or student* or school* or pupil*) ) |  |
|  | DE "CHILDREN" OR DE "SCHOOL children" |  |
|  | DE "TEENAGERS" |  |
|  | DE "STUDENTS" |  |
|  | S1 OR S2 OR S3 OR S4 |  |
|  | TI ( ("physical* adj activ*" or sport* or cycle or cycling or bicycling or bicycle* or walk* or "physical education" or "physical training" or exercis* or (energy adj expenditure) or danc* or inactiv* or "physical fitness" or lifestyle or "active lesson*" or "aerobic fitness" or sedentar* or sit*)) OR AB ( ("physical* adj activ*" or sport* or cycle or cycling or bicycling or bicycle* or walk* or "physical education" or "physical training" or exercis* or (energy adj expenditure) or danc* or inactiv* or "physical fitness" or lifestyle or "active lesson*" or "aerobic fitness" or sedentar* or sit*)) |  |
|  | DE "SPORTS" |  |
|  | DE "EXERCISE" |  |
|  | DE "SEDENTARY behavior" |  |
|  | DE "PHYSICAL fitness" |  |
|  | DE "PHYSICAL education" |  |
|  | S6 OR S7 OR S8 OR S9 OR S10 OR S11 |  |
|  | TI ( ("control* trial" or randomi* or randomly or trial* or evaluation or effect* or control* or cluster or intervention or pilot or feasibility or prospective* or observation* or longitudinal or follow-up* or followup* or (follow* adj up*) or cohort* or "process evaluation")) OR AB ( ("control* trial" or randomi* or randomly or trial* or evaluation or effect* or control* or cluster or intervention or pilot or feasibility or prospective* or observation* or longitudinal or follow-up* or followup* or (follow* adj up*) or cohort* or "process evaluation")) |  |
|  | TI ( ("case study" or "case report" or "abstract report" or letter) ) OR AB ( ("case study" or "case report" or "abstract report" or letter) ) |  |
|  | S13 NOT S14 |  |
|  | TI ( (acceleromet* or accelerometer-assessed or "counts per minute" or CPM or actigraph* or "heart rate" or pedomet* or "objective* measur*" or "device measur*" or "activity monitor" or MVPA or LIPA or ((process or program*) adj (evaluation* or monitoring)) or (qualitative adj (component* aspect* or approach*)) or participant observation* or fidelity or adher* or monitor* or implement* or integrat* or adopt* or uptake or dosage or reach or dose or quality or sustainabil* or implement*) ) OR AB ( (acceleromet* or accelerometer-assessed or "counts per minute" or CPM or actigraph* or "heart rate" or pedomet* or "objective* measur*" or "device measur*" or "activity monitor" or MVPA or LIPA or ((process or program*) adj (evaluation* or monitoring)) or (qualitative adj (component* or aspect* or approach*)) or participant observation* or fidelity or adher* or monitor* or implement* or integrat* or adopt* or uptake or dosage or reach or dose or quality or sustainabil* or implement*) ) |  |
|  | TI ( (school* or educat* or teach* or academi*) ) OR AB ( (school* or educat* or teach* or academi*) ) |  |
|  | S5 AND S12 AND S15 AND S16 AND S17 | 5,810 |
|  | S5 AND S12 AND S15 AND S16 AND S17 Year limits applied | **2,471** |
