# Supplementary material for: Features of effective staff training programmes within school-based interventions targeting student activity behaviour: a systematic review and meta-analysis
Source: Int J Behav Nutr Phys Act. 2022 Sep 24;19:125. doi: 10.1186/s12966-022-01361-6 (PMC9509574; doi:10.1186/s12966-022-01361-6)
Supplement: Supplementary file 2 — Additional file 2. Structured process to classify fidelity outcomes. [file 12966_2022_1361_MOESM2_ESM.docx]

Additional File 2. Structured process to classify fidelity outcomes

**Context:**

To be eligible for inclusion, studies had to report either staff fidelity (any quantitative measure), or any pre-post device measured physical activity behaviour

**Definitions:**

**Fidelity**: “the extent to which the intervention is delivered^[[1]](#footnote-1)^ as intended” [1]

**Issue:**

We observed considerable methodological and conceptual heterogeneity for fidelity measures. Intervention evaluation authors either provided different definitions of fidelity, or definitions were not clearly stated. Perhaps as a result, we observed wide variation in measures of fidelity, the number of fidelity measures taken, the number of time points where fidelity measures were taken, and the level at which fidelity outcomes were measured (e.g. at school vs teacher level). To account for these differences, a systematised approach was employed to categorise fidelity outcomes reported into three groups: low, medium and high fidelity. This approach is outlined below.

**Approach:**

Measures that solely focus on teachers/other school staff fidelity to the intervention were prioritised above summary scores that include other actors’ roles in the intervention (e.g. how active children were in new PE programme). Where multiple fidelity outcomes were reported for feasibility, efficacy and effectiveness trials reported within a paper, the effectiveness fidelity outcome was used (in line with review inclusion criteria). Where multiple teacher/school staff measures of fidelity were reported, a summary score was calculated; the mean of multiple teacher/staff fidelity scores was taken as the indicator of overall fidelity. Authors’ own overall interpretation of the overall fidelity score they report was not be used (e.g. “teachers delivered the intervention with high fidelity”). The authors must have provided an overall or component specific quantitative target outcome score to compare the reported fidelity score against (e.g. teachers were requested to deliver 3 active maths lessons per week, and initiate one recess activity per week). Fidelity outcomes were thereafter interpreted using the following criteria: staff fidelity was considered high, medium or low if the reported or calculated fidelity outcomes were between 80-100%, 50-79% or 0-49% respectively [2]. In cases where calculations resulted in decimal portions, values greater than or equal to 0.5 were rounded up; all others values were rounded down. Where fidelity scores at multiple time points were reported, the fidelity measure taken at the time point closest to the exposure of interest (i.e. the staff training) was used.

**Studies excluded from fidelity outcomes:**

Given that the definition of fidelity adopted for the purposes for this review was “the extent to which teachers delivered the intervention as intended”[1], studies that solely reported fidelity outcomes in the absence of any quantitative goal/aim to compare outcomes against were excluded (e.g. fidelity scores were solely compared against the control group only or fidelity outcomes reported in other studies).

**References**

1. Moore GF, Audrey S, Barker M, Bond L, Bonell C, Hardeman W, Moore L, O'Cathain A, Tinati T, Wight D *et al*. Process evaluation of complex interventions: Medical Research Council guidance. *BMJ* 2015, 350:h1258.

2. Borrelli B. The assessment, monitoring, and enhancement of treatment fidelity in public health clinical trials. *J Public Health Dent* 2011, 71:S52-S63.

1. Delivered by a teacher/or other member of school staff within an intervention arm [↑](#footnote-ref-1)
