# Supplementary material for: Features of effective staff training programmes within school-based interventions targeting student activity behaviour: a systematic review and meta-analysis
Source: Int J Behav Nutr Phys Act. 2022 Sep 24;19:125. doi: 10.1186/s12966-022-01361-6 (PMC9509574; doi:10.1186/s12966-022-01361-6)
Supplement: Supplementary file 3 — Additional file 3. Hierarchies used to select activity behaviour outcomes. [file 12966_2022_1361_MOESM3_ESM.doc]

Additional File 3. Hierarchies used to select activity behaviour outcomes

Where more than one of either activity behaviour reported at both baseline and follow-up within a study, we applied a hierarchy to focus on outcomes closest to the review’s exposure of interest (i.e. staff training). The hierarchies we used to select outcomes for both activity behaviours are outlined below. Where multiple follow-up measures were reported for either activity behaviour were reported, outcomes measured closest to the end of the student-targeted intervention were extracted (i.e. post-intervention priortised above mid-intervention or maintenance measures).

**Physical activity outcomes were selected in the following numerical and then alphabetical order**

1. **Teacher period (e.g. active lesson only)**
2. time spent in moderate-to-vigorous physical activity
3. total physical activity
4. vigorous physical activity
5. moderate physical activity
6. light physical activity
7. **School hours (e.g. 09:00-15:00)**
8. time spent in moderate-to-vigorous physical activity
9. total physical activity
10. vigorous physical activity
11. moderate physical activity
12. light physical activity
13. **Weekdays (e.g. Monday 07:00-21:00)**
14. time spent in moderate-to-vigorous physical activity
15. total physical activity
16. vigorous physical activity
17. moderate physical activity
18. light physical activity
19. **Whole of week (including weekends)**
20. time spent in moderate-to-vigorous physical activity
21. total physical activity
22. vigorous physical activity
23. moderate physical activity
24. light physical activity

**Sedentary behaviour outcomes were selected in the following numerical and then alphabetical order**

1. **Teacher period (e.g. active lesson only)**
2. time spent in any sedentary behavior
3. **School hours (e.g. 09:00-15:00)**
4. time spent in any sedentary behavior
5. **Weekdays (e.g. Monday 07:00-21:00)**
6. time spent in any sedentary behavior
7. **Whole of week (including weekends)**
8. time spent in any sedentary behavior
