# Supplementary material for: Features of effective staff training programmes within school-based interventions targeting student activity behaviour: a systematic review and meta-analysis
Source: Int J Behav Nutr Phys Act. 2022 Sep 24;19:125. doi: 10.1186/s12966-022-01361-6 (PMC9509574; doi:10.1186/s12966-022-01361-6)
Supplement: Supplementary file 4 — Additional file 4. Formulae used for meta-analyses of physical activity and sedentary behaviour outcomes. [file 12966_2022_1361_MOESM4_ESM.docx]

Additional File 4. Formulae used for meta-analyses of physical activity and sedentary behaviour outcomes.

Standardised mean differences (SMDs) were calculated based on the number, mean, and standard deviations (SDs) of the treatment and control groups at baseline and follow-up. Formulae used for physical activity (PA) outcomes are outlined below and based on the Cochrane handbook [1]. The same formulae were used for sedentary behaviour analyses.

We calculated the difference in means (MD) between treatment (E) and control groups (C) as:

MD= M_E_ - M_C_

where M_E_ is the mean difference between baseline and follow-up in the treatment group and M_c_ is the mean difference between baseline and follow-up in the control group.

We imputed the change-from-baseline SDs (SD’change) for treatment and control groups separately using a correlation coefficient (Corr):

SD’change=√SD^2^,baseline +SD^2^,follow-up - (2*Corr*SD,baseline* SD,follow-up),

where Corr was set at 0.5 [2] based on similar reviews and studies [3, 4]. We performed sensitivity analyses by varying the correlation coefficient between 0.5 and 0.95.

Given that groups were dissimilar in size, we calculated the weighted and pooled SD (SD*pooled) of MDs for treatment and control groups as:

SD_pool=√(((n_c_-1)*SD’change_c_^2^+(n_E_-1)* SD’change_E_^2^)/(N-2))

where n_c_ is the is the number of participants in the control group, SD’change_c_ is the change-from-baseline SD in the control group, n_E_ is the number of participants in the treatment group, SD’change_E_ is the change-from-baseline SD in the treatment group and N is the number of participants in treatment and control groups.

The SMD was calculated as:

SMD=(MD)/SD_pool

A bias correction (SMD_bias) using Hedges’ adjusted *g* was applied to studies that reported on small sample sizes (defined as <50 participants):

SMD_bias = SMD*(1-3/(4*N-9))

where N is the number of participants in treatment and control groups.

The standardised error (SE) of the SMD (SE(SMD)) was calculated as:

SE(SMD)=√ (1/(N)+SMD^2^/(2*N)))*√ (2*(1-Corr))[5],

where N is the number of participants in treatment and control groups and Corr was set at 0.5.

The SEs of SMDs were corrected using the intra-cluster correlation coefficient (ICC), where reported. The design effect (DE) was calculated as:

DE=1+ (*m*-1)**ρ* [6]*,*

where *m* is the average cluster size and *ρ* is the ICC. Where study authors did not report the ICC, we used estimates from similar studies (0.11 for physical activity; 0.22 for sedentary behaviour [7]).

Estimated design effects were used to produce an adjusted SE (SE_adj):

SE_adj=√ (DE)*SE(SMD).

SMDs and their adjusted SEs were used to perform random-effects meta-analyses.

**References**

1. Deeks JJ HJ, Altman DG (editors). Chapter 10: Analysing data and undertaking meta-analyses. In: Higgins JPT, Thomas J, Chandler J, Cumpston M, Li T, Page MJ, Welch VA (editors). In: *Cochrane Handbook for Systematic Reviews of Interventions version 63 (updated February 2022).* edn. Cochrane 2022. Available from <www.training.cochrane.org/handbook>.; 2022.

2. Morris SB. Estimating Effect Sizes From Pretest-Posttest-Control Group Designs. *Organizational Research Methods* 2008, 11(2):364-386.

3. Jones M, Defever E, Letsinger A, Steele J, Mackintosh KA. A mixed-studies systematic review and meta-analysis of school-based interventions to promote physical activity and/or reduce sedentary time in children. *Journal of sport and health science* 2020, 9(1):3-17.

4. Corder K, Sharp SJ, Jong ST, Foubister C, Brown HE, Wells EK, Armitage SM, Croxson CH, Vignoles A, Wilkinson PO. Effectiveness and cost-effectiveness of the GoActive intervention to increase physical activity among UK adolescents: A cluster randomised controlled trial. *PLoS Med* 2020, 17(7):e1003210.

5. Higgins JP, Thomas J, Chandler J, Cumpston M, Li T, Page MJ, Welch VA. Cochrane handbook for systematic reviews of interventions: John Wiley & Sons; 2019.

6. Higgins JPT ES, Li T (editors). Chapter 23: Including variants on randomized trials. In: Higgins JPT, Thomas J, Chandler J, Cumpston M, Li T, Page MJ, Welch VA (editors). In: *Cochrane Handbook for Systematic Reviews of Interventions version 63 (updated February 2022).* edn. Cochrane, 2022. Available from <www.training.cochrane.org/handbook>.; 2022.

7. van Sluijs EMF, Jones NR, Jones AP, Sharp SJ, Harrison F, Griffin SJ. School-level correlates of physical activity intensity in 10-year-old children. *Int J Pediatr Obes* 2011, 6(2-2):e574-e581.
