# Supplementary material for: Features of effective staff training programmes within school-based interventions targeting student activity behaviour: a systematic review and meta-analysis
Source: Int J Behav Nutr Phys Act. 2022 Sep 24;19:125. doi: 10.1186/s12966-022-01361-6 (PMC9509574; doi:10.1186/s12966-022-01361-6)
Supplement: Supplementary file 5 — Additional file 5. Publications excluded with reasons at stages 1 and 2 of full-text screening. [file 12966_2022_1361_MOESM5_ESM.docx]

Additional File 5. Publications excluded with reasons at stages 1 and 2 of full-text screening

| **Title** | **Authors** | **Year** | **Exclusion reason** |
| --- | --- | --- | --- |
| **Stage 1** (n=490) |  |  |  |
| No title identified | Lawlor et al | 2016 | Exclusion reason: stage 1 - Conference abstract/poster |
| The effect of a school-based active video game intervention on children's aerobic fitness, physical activity level, and exercise related psychological variables: A preliminary RCT trial | Lau et al | 2015 | Exclusion reason: stage 1 - Conference abstract/poster |
| Bright spots, physical activity investments that work: the Finnish Schools on the Move programme | Blom et al | 2018 | Exclusion reason: stage 1 - Conference abstract/poster |
| Bright spots physical activity investments that work: Youth-Physical Activity Towards Health (Y-PATH) | Belton et al | 2019 | Exclusion reason: stage 1 - Conference abstract/poster |
| The impact of long-term school-based physical activity interventions on body mass index of primary school children - a meta-analysis of randomized controlled trials | Mei et al | 2016 | Exclusion reason: stage 1 - Conference abstract/poster |
| Partnering with Schools to Implement Physical Activity Interventions | McClary King et al | 2018 | Exclusion reason: stage 1 - Conference abstract/poster |
| Understanding "agency" in the translation of a health promotion program | Page-Reeves et al | 2015 | Exclusion reason: stage 1 - Conference abstract/poster |
| Scaling-up an efficacious comprehensive school-based physical activity intervention: development, evaluation and dissemination of the iPLAY program | Lonsdale et al | 2019 | Exclusion reason: stage 1 - Conference abstract/poster |
| The mediating effects of breaking up classroom sitting with cognitively engaging or simple active breaks on children's cognition | Mazzoli et al | 2019 | Exclusion reason: stage 1 - Conference abstract/poster |
| The Application of an Implementation Science Framework to Comprehensive School Physical Activity Programs: Be a Champion! | Moore et al | 2018 | Exclusion reason: stage 1 - Conference abstract/poster |
| Physically Active Lessons: Evaluation Report and Executive Summary | Miller et al | 2015 | Exclusion reason: stage 1 - Conference abstract/poster |
| Effects of a school-based intervention program on metabolic syndrome parameters in school-aged youth | Cocca et al | 2016 | Exclusion reason: stage 1 - Conference abstract/poster |
| Bright spots, physical activity investments that work: JUMP-in: promoting physical activity and healthy nutrition at primary schools in Amsterdam | Busch et al | 2018 | Exclusion reason: stage 1 - Conference abstract/poster |
| Beyond the randomised controlled trial and BMI--evaluation of effectiveness of through-school nutrition and physical activity programmes | Rush et al | 2015 | Exclusion reason: stage 1 - Conference abstract/poster |
| Are benefits from an 8-month exercise intervention in pre-and peri-pubertal children maintained after 1 year of detraining? Follow-up data from the CAPO kids trial | Beck et al | 2017 | Exclusion reason: stage 1 - Conference abstract/poster |
| Adding Context: Process evaluation of a childhood obesity prevention trial (the WAVES study) | Griffin et al | 2015 | Exclusion reason: stage 1 - Conference abstract/poster |
| Process evaluation of the school-based Girls Active programme | Gorely et al | 2019 | Exclusion reason: stage 1 - Conference abstract/poster |
| The physical education and physical literacy (pepl) approach: a multicomponent primary school intervention targeting physical literacy | Telford et al | 2019 | Exclusion reason: stage 1 - Conference abstract/poster |
| A Process Evaluation of A Fitness Curriculum To Meet Physical Activity Policy Requirements: A Pilot Study | Esquivel et al | 2019 | Exclusion reason: stage 1 - Conference abstract/poster |
| A little more time around the track may go a long way: Implications of increasing moderate to vigorous physical activity in pre-adolescents | Farukhi et al | 2019 | Exclusion reason: stage 1 - Conference abstract/poster |
| A 7-year school-based exercise intervention improves musculoskeletal traits in both genders and reduces in girls with each year with the program the fracture risk | Rosengren et al | 2015 | Exclusion reason: stage 1 - Conference abstract/poster |
| Impact of height-adjustable desks on adolescents' energy expenditure, adiposity and perceived musculoskeletal discomfort | Ayala et al | 2019 | Exclusion reason: stage 1 - Conference abstract/poster |
| Research on the influence of Tai Chi teaching on physical fitness based on virtual reality technology | Wang et al | 2018 | Exclusion reason: stage 1 - Conference abstract/poster |
| Integrating movement in academic classrooms: Understanding, applying and advancing the knowledge base | Webster et al | 2015 | Exclusion reason: stage 1 - Conference abstract/poster |
| Research on the construction of student' physical fitness assessment model from the perspective of health promotion | Yan et al | 2018 | Exclusion reason: stage 1 - Conference abstract/poster |
| Effect of Active Lessons on Physical Activity, Academic, and Health Outcomes: A Systematic Review | Martin et al | 2017 | Exclusion reason: stage 1 - Conference abstract/poster |
| Evaluation of school-based interventions of active breaks in primary schools: A systematic review and meta-analysis | Masini et al | 2020 | Exclusion reason: stage 1 - Conference abstract/poster |
| A Review of Implementation Outcome Measures of School-based Physical Activity Interventions | Shah et al | 2017 | Exclusion reason: stage 1 - Conference abstract/poster |
| The effectiveness of interventions on sustained childhood physical activity: A systematic review and meta-analysis of controlled studies | Sims et al | 2015 | Exclusion reason: stage 1 - Conference abstract/poster |
| Pedagogical Approaches to and Effects of Fundamental Movement Skill Interventions on Health Outcomes: A Systematic Review | Tompsett et al | 2017 | Exclusion reason: stage 1 - Conference abstract/poster |
| Indoor school environments, physical activity, sitting behaviour and pedagogy: a scoping review | Ucci et al | 2015 | Exclusion reason: stage 1 - Conference abstract/poster |
| Effect of classroom-based physical activity interventions on academic and physical activity outcomes: A systematic review and meta-analysis | Watson et al | 2017 | Exclusion reason: stage 1 - Conference abstract/poster |
| School-based intervention programs for preventing obesity and promoting physical activity and fitness: A systematic review | Yuksel et al | 2020 | Exclusion reason: stage 1 - Conference abstract/poster |
| Physical activity interventions and nutritional education to combat childhood obesity in school: Systematic review | Serra et al | 2018 | Exclusion reason: stage 1 - Conference abstract/poster |
| A mixed-studies systematic review and meta-analysis of school-based interventions to promote physical activity and/or reduce sedentary time in children | Jones et al | 2020 | Exclusion reason: stage 1 - Conference abstract/poster |
| Effect of school-based interventions to control childhood obesity: A review of reviews | Amini et al | 2015 | Exclusion reason: stage 1 - Conference abstract/poster |
| Characteristics of physical activity interventions and effects on cardiorespiratory fitness in children aged 6-12 years-A systematic review | Braaksma et al | 2018 | Exclusion reason: stage 1 - Conference abstract/poster |
| Efficacy of School-Based Interventions for Improving Muscular Fitness Outcomes in Adolescent Boys: A Systematic Review and Meta-analysis | Cox et al | 2020 | Exclusion reason: stage 1 - Conference abstract/poster |
| Utilising active play interventions to promote physical activity and improve fundamental movement skills in children: a systematic review and meta-analysis | Johnstone et al | 2018 | Exclusion reason: stage 1 - Conference abstract/poster |
| What Works in Sedentary Behavior Interventions for Youth: A Review of Reviews | dos Santos et al | 2019 | Exclusion reason: stage 1 - Conference abstract/poster |
| A systematic review of randomized and case‐controlled trials investigating the effectiveness of school‐based motor skill interventions in 3‐to 12‐year‐old children. | Eddy et al | 2019 | Exclusion reason: stage 1 - Conference abstract/poster |
| The Project P.A.T.H.S. in Hong Kong: Work Done and Lessons Learned in a Decade | Shek et al | 2016 | Exclusion reason: stage 1 - Conference abstract/poster |
| Developmental Physical Education: How to Implement a Peer-assistance Program to Help Low Performers | Gagnon et al | 2016 | Exclusion reason: stage 1 - Conference abstract/poster |
| Systematic Review of Physical Education-Based Physical Activity Interventions Among Elementary School Children | Errisuriz et al | 2018 | Exclusion reason: stage 1 - Conference abstract/poster |
| School and family-based interventions for promoting a healthy lifestyle among children and adolescents in Italy: a systematic review | Gorga et al | 2016 | Exclusion reason: stage 1 - Conference abstract/poster |
| Measuring implementation fidelity of school-based obesity prevention programmes: A systematic review | Schaap et al | 2018 | Exclusion reason: stage 1 - Conference abstract/poster |
| A systematic review of school-based physical activity interventions on children's wellbeing | Rafferty et al | 2016 | Exclusion reason: stage 1 - Conference abstract/poster |
| Effectiveness of school-based physical activity programmes on cardiorespiratory fitness in children: a meta-analysis of randomised controlled trials | Pozuelo-Carrascosa et al | 2018 | Exclusion reason: stage 1 - Conference abstract/poster |
| Implementing health promotion programmes in schools: a realist systematic review of research and experience in the United Kingdom | Pearson et al | 2015 | Exclusion reason: stage 1 - Conference abstract/poster |
| Physically active lessons as physical activity and educational interventions: A systematic review of methods and results | Norris et al | 2015 | Exclusion reason: stage 1 - Conference abstract/poster |
| Physical activity interventions in schools for improving lifestyle in European countries | Mura et al | 2015 | Exclusion reason: stage 1 - Conference abstract/poster |
| Classroom standing desks and sedentary behavior: A systematic review | Minges et al | 2016 | Exclusion reason: stage 1 - Conference abstract/poster |
| School-Based Interventions to Improve Cardiorespiratory Fitness in Adolescents: Systematic Review with Meta-analysis | Minatto et al | 2016 | Exclusion reason: stage 1 - Conference abstract/poster |
| Classroom-Based Physical Activity and Sedentary Behavior Interventions in Adolescents: A Systematic Review and Meta-Analysis | McMichan et al | 2018 | Exclusion reason: stage 1 - Conference abstract/poster |
| Evaluation of physical activity interventions in children via the reach, efficacy/effectiveness, adoption, implementation, and maintenance (RE-AIM) framework: A systematic review of randomized and non-randomized trials | McGoey et al | 2016 | Exclusion reason: stage 1 - Conference abstract/poster |
| Studies of Physical Education in the United States Using SOFIT: A Review | McKenzie et al | 2017 | Exclusion reason: stage 1 - Conference abstract/poster |
| Interventions aimed at preventing and reducing overweight/obesity among children and adolescents: a meta-synthesis | Kobes et al | 2018 | Exclusion reason: stage 1 - Conference abstract/poster |
| Ceria, respek, gigih, aktif, sihat (C.E.R.G.A.S.): Factors influencing sustainability of a school-based obesity intervention for young adolescents | Hoe et al | 2019 | Exclusion reason: stage 1 - Conference abstract/poster |
| Academic, cognitive and physical outcomes of two strategies to integrate movement in classroom: active lessons and active breaks | Mendez-Gimenez et al | 2020 | Exclusion reason: stage 1 - Conference abstract/poster |
| A school-based health promotion program to promote physical activity among young adolescents in Hong Kong | Abraham et al | 2015 | Exclusion reason: stage 1 - Conference abstract/poster |
| Evaluation of the Carol M. White Physical Education Program: Final Report | Jones et al | 2015 | Exclusion reason: stage 1 - Conference abstract/poster |
| Increasing Children's Physical Activity During the School Day | Hatfield et al | 2015 | Exclusion reason: stage 1 - Conference abstract/poster |
| A new way to prevent obesity for kids aged between 8-10 years | Knopf et al | 2018 | Exclusion reason: stage 1 - Conference abstract/poster |
| Adoption, implementation and sustainability of school-based physical activity and sedentary behaviour interventions in real-world settings: A systematic review | Cassar et al | 2019 | Exclusion reason: stage 1 - Conference abstract/poster |
| The perfect physical activity program-to not reduce adolescent risk | Feldstein Ewing et al | 2017 | Exclusion reason: stage 1 - Conference abstract/poster |
| Scale-up and dissemination of a school-based resistance training program: RE-AIM evaluation of impact | Kennedy et al | 2019 | Exclusion reason: stage 1 - Conference abstract/poster |
| Feasibility of the SWITCH Classroom Module and its Effect on Classroom Engagement | Long et al | 2016 | Exclusion reason: stage 1 - Conference abstract/poster |
| United for healthier kids program experience-is it on track to achieve its goals: Country experiences from Pakistan and Mexico | Das et al | 2017 | Exclusion reason: stage 1 - Conference abstract/poster |
| 'Fitness for princess'-a study to improve physical fitness among school going children in Western India | Harihara Prakash et al | 2015 | Exclusion reason: stage 1 - Conference abstract/poster |
| Health education program at the "happy life, healthy heart" school: A randomized clinical trial | Alievi Mari et al | 2019 | Exclusion reason: stage 1 - Conference abstract/poster |
| Implementation of Brain Breaks in the Classroom and Effects on Attitudes toward Physical Activity in a Macedonian School Setting | Popeska et al | 2018 | Exclusion reason: stage 1 - Duplicate |
| Trends in physical activity, health-related fitness, and gross motor skills in children during a two-year comprehensive school physical activity program | Brusseau et al | 2018 | Exclusion reason: stage 1 - Insufficient detail on teacher role |
| The ‘uptake’ of a sport-for-development programme in South Africa | Burnett et al | 2015 | Exclusion reason: stage 1 - Insufficient detail on teacher role |
| The impact of playworks on boys' and girls' physical activity during recess | Bleeker et al | 2015 | Exclusion reason: stage 1 - Insufficient detail on teacher role |
| Exploring the impact of high intensity interval training on adolescents' objectively measured physical activity: Findings from a randomized controlled trial | Costigan et al | 2018 | Exclusion reason: stage 1 - Insufficient detail on teacher role |
| Use of Stand-Biased Desks to Reduce Sedentary Time in High School Students: A Pilot Study | Pickens et al | 2016 | Exclusion reason: stage 1 - Insufficient detail on teacher role |
| Long-term effects of comprehensive school health on health-related knowledge, attitudes, self-efficacy, health behaviours and weight status of adolescents | Ofosu et al | 2018 | Exclusion reason: stage 1 - Insufficient detail on teacher role |
| Evaluation of a Walking-Track Intervention to Increase Children's Physical Activity during Primary School Break Times | Powell et al | 2018 | Exclusion reason: stage 1 - Insufficient detail on teacher role |
| Health Empowers You: Impact of a School-Based Physical Activity Program in Elementary School Students, Georgia, 2015-2016 | Hyde et al | 2020 | Exclusion reason: stage 1 - Insufficient detail on teacher role |
| Implementation intentions improve exercise self-efficacy and exercise behavior regardless of task difficulty | Shen et al | 2019 | Exclusion reason: stage 1 - Insufficient detail on teacher role |
| A Multi-Week Assessment of a Mobile Exergame Intervention in an Elementary School | Garde et al | 2018 | Exclusion reason: stage 1 - Insufficient detail on teacher role |
| Increasing physical activity of children during school recess | Hayes et al | 2015 | Exclusion reason: stage 1 - Insufficient detail on teacher role |
| Evaluation of a Novel Mobile Exergame in a School-Based Environment | Garde et al | 2016 | Exclusion reason: stage 1 - Insufficient detail on teacher role |
| Trajectories of objectively measured sedentary time among secondary students in Manitoba, Canada in the context of a province-wide physical education policy: A longitudinal analysis | Zuo et al | 2016 | Exclusion reason: stage 1 - Insufficient detail on teacher role |
| Measuring the Implementation of a School Wellness Policy | Snelling et al | 2017 | Exclusion reason: stage 1 - Insufficient detail on teacher role |
| Elementary School-Based Obesity Intervention Using an Educational Curriculum | Lynch et al | 2016 | Exclusion reason: stage 1 - Insufficient detail on teacher role |
| Long term impact of one daily unit of physical exercise at school on cardiovascular risk factors in school children | Muller et al | 2016 | Exclusion reason: stage 1 - Insufficient detail on teacher role |
| Moderate-to-vigorous physically active academic lessons and academic engagement in children with and without a social disadvantage: a within subject experimental design | Mullender-Wijnsma et al | 2015 | Exclusion reason: stage 1 - Insufficient detail on teacher role |
| Effectiveness of the Healthy Lifestyles Programme (HeLP) to prevent obesity in UK primary-school children: a cluster randomised controlled trial | Lloyd et al | 2018 | Exclusion reason: stage 1 - Insufficient detail on training or no training provided |
| Improving Cognitive Performance of 9-12 Years Old Children: Just Dance? A Randomized Controlled Trial | van den Berg et al | 2019 | Exclusion reason: stage 1 - Insufficient detail on training or no training provided |
| PACE: A group randomised controlled trial to increase children's break-time playground physical activity | Parrish et al | 2016 | Exclusion reason: stage 1 - Insufficient detail on training or no training provided |
| Unravelling the Effects of the Healthy Primary School of the Future: For Whom and Where Is It Effective? | Bartelink et al | 2019 | Exclusion reason: stage 1 - Insufficient detail on training or no training provided |
| Evaluation of a concept-based physical education unit for energy balance education | Chen et al | 2018 | Exclusion reason: stage 1 - Insufficient detail on training or no training provided |
| How Feedback and Goal-Setting Impact Children's Recess Physical Activity | Koufoudakis et al | 2016 | Exclusion reason: stage 1 - Insufficient detail on training or no training provided |
| Promoting physical activity at the school playground: a quasi-experimental intervention study | López-Fernández et al | 2016 | Exclusion reason: stage 1 - Insufficient detail on training or no training provided |
| Feasibility of implementing an outdoor walking break in Italian middle schools | Brustio et al | 2018 | Exclusion reason: stage 1 - Insufficient detail on training or no training provided |
| Effects of Common Core State Standards on Student Physical Activity Rates and Student and Teacher Perceptions in Physical Education | Seymour et al | 2019 | Exclusion reason: stage 1 - Insufficient detail on training or no training provided |
| Evaluation of Daily Physical Activity (DPA) policy implementation in Ontario: surveys of elementary school administrators and teachers | Allison et al | 2016 | Exclusion reason: stage 1 - Insufficient detail on training or no training provided |
| How 'The Daily Mile^TM^' works in practice: A process evaluation in a UK primary school | Harris et al | 2019 | Exclusion reason: stage 1 - Insufficient detail on training or no training provided |
| Exploring the effectiveness of a school-based physical activity policy in British Columbia, Canada: a mixed-methods observational study | Weatherson et al | 2019 | Exclusion reason: stage 1 - Insufficient detail on training or no training provided |
| Lessons learned and insights from the implementation of a food and physical activity policy to prevent obesity in Mexican schools: An analysis of nationally representative survey results | Theodore et al | 2018 | Exclusion reason: stage 1 - Insufficient detail on training or no training provided |
| The effect of a school-centered multicomponent intervention on daily physical activity and sedentary behavior in primary school children: The Active Living study | Van Kann et al | 2016 | Exclusion reason: stage 1 - Insufficient detail on training or no training provided |
| Effect of gymnastic school lessons on the student's physical activity levels: jump in young and adult education (YAE) | Batista Lemes et al | 2017 | Exclusion reason: stage 1 - Insufficient detail on training or no training provided |
| Maybe it is not a goal that matters: a report from a physical activity intervention in youth | Bronikowski et al | 2018 | Exclusion reason: stage 1 - Insufficient detail on training or no training provided |
| COPE: A Pilot Study With Urban-Dwelling Minority Sixth-Grade Youth to Improve Physical Activity and Mental Health Outcomes | Hoying et al | 2016 | Exclusion reason: stage 1 - Insufficient detail on training or no training provided |
| The relationship between school-level characteristics and implementation fidelity of a coordinated school health childhood obesity prevention intervention | Lederer et al | 2015 | Exclusion reason: stage 1 - Insufficient detail on training or no training provided |
| The Daily Mile as a public health intervention: a rapid ethnographic assessment of uptake and implementation in South London, UK | Hanckel et al | 2019 | Exclusion reason: stage 1 - Insufficient detail on training or no training provided |
| Effects of School Gardening Lessons on Elementary School Children's Physical Activity and Sedentary Time | Rees-Punia et al | 2017 | Exclusion reason: stage 1 - Insufficient detail on training or no training provided |
| Impact of goal setting on physical activity in physical education | Chase et al | 2018 | Exclusion reason: stage 1 - Insufficient detail on training or no training provided |
| Active and Healthy Lifestyle - Nationwide Programs in Israeli Schools | Zach et al | 2018 | Exclusion reason: stage 1 - Insufficient detail on training or no training provided |
| Changes in school-day step counts during a physical activity for Lent intervention: a cluster randomized crossover trial of the Savior's Sandals | Kahan et al | 2019 | Exclusion reason: stage 1 - Insufficient detail on training or no training provided |
| Active learning improves on-task behaviors in 4th grade children | Bartholomew et al | 2018 | Exclusion reason: stage 1 - Insufficient detail on training or no training provided |
| Intervention fidelity in the definitive cluster randomised controlled trial of the Healthy Lifestyles Programme (HeLP) trial: Findings from the process evaluation | Lloyd et al | 2017 | Exclusion reason: stage 1 - Insufficient detail on training or no training provided |
| Effects of Sharing Data With Teachers on Student Physical Activity and Sedentary Behavior in the Classroom | Hodgin et al | 2020 | Exclusion reason: stage 1 - Insufficient detail on training or no training provided |
| A classroom-based physical activity intervention for urban kindergarten and first-grade students: a feasibility study | Reznik et al | 2015 | Exclusion reason: stage 1 - Insufficient detail on training or no training provided |
| Effects of Music on Physical Activity Rates of Elementary Physical Education Students | Barney et al | 2015 | Exclusion reason: stage 1 - Insufficient detail on training or no training provided |
| An integrated curriculum approach to increasing habitual physical activity in deprived South Asian children | Eyre et al | 2016 | Exclusion reason: stage 1 - Insufficient detail on training or no training provided |
| Implementation of Local Wellness Policies in Schools: Role of School Systems, School Health Councils, and Health Disparities | Hager et al | 2016 | Exclusion reason: stage 1 - Insufficient detail on training or no training provided |
| The Effect of a Comprehensive School Physical Activity Program on Physical Activity and Health-Related Fitness in Children From Low-Income Families | Brusseau et al | 2016 | Exclusion reason: stage 1 - Led by external provider/specialist |
| The effect of sport for LIFE: All island in children from low socio-economic status: A clustered randomized controlled trial | Breslin et al | 2019 | Exclusion reason: stage 1 - Led by external provider/specialist |
| Maximizing children's physical activity using the LET US Play principles | Brazendale et al | 2015 | Exclusion reason: stage 1 - Led by external provider/specialist |
| Pragmatic evaluation of the Go2Play Active Play intervention on physical activity and fundamental movement skills in children | Johnstone et al | 2017 | Exclusion reason: stage 1 - Led by external provider/specialist |
| The effect of educational intervention based on the Theory of Planned Behavior on thephysical activity of female students in Behbehan City (2016) | Leila et al | 2017 | Exclusion reason: stage 1 - Led by external provider/specialist |
| The effects of integrating physical activity into mathematic lessons on mathematic test performance, body mass index and short term memory among 10 year old children | Fakri et al | 2020 | Exclusion reason: stage 1 - Led by external provider/specialist |
| Proposal for an Enhanced Physical Education Program in the Primary School: Evaluation of Feasibility and Effectiveness in Improving Physical Skills and Fitness | Dallolio et al | 2016 | Exclusion reason: stage 1 - Led by external provider/specialist |
| Fit "N" Cool Kids: The Effects of Character Modeling and Goal Setting on Children's Physical Activity and Fruit and Vegetable Consumption | Larson et al | 2018 | Exclusion reason: stage 1 - Led by external provider/specialist |
| Evaluation of the computer-based intervention program stayingfit Brazil to promote healthy eating habits: The results from a school cluster-randomized controlled trial | Da Silva et al | 2019 | Exclusion reason: stage 1 - Led by external provider/specialist |
| Effects of School-based Health Promotion Intervention on Health Behaviors among School Adolescents in North Lima and Callao, Peru | Sharma et al | 2018 | Exclusion reason: stage 1 - Led by external provider/specialist |
| Cost and Cost-Effectiveness of Students for Nutrition and eXercise (SNaX) | Ladapo et al | 2016 | Exclusion reason: stage 1 - Led by external provider/specialist |
| Preliminary Efficacy and Feasibility of "Thinking While Moving in English": A Program with Physical Activity Integrated into Primary School English Lessons | Mavilidi et al | 2018 | Exclusion reason: stage 1 - Led by external provider/specialist |
| Physically active vs. sedentary academic lessons: A dose response study for elementary student time on task | Grieco et al | 2016 | Exclusion reason: stage 1 - Led by external provider/specialist |
| Mediators of Physical Activity Behavior Change in the "Girls on the Move" Intervention | Robbins et al | 2019 | Exclusion reason: stage 1 - Led by external provider/specialist |
| Integrating mindfulness training in school health education to promote healthy behaviors in adolescents: Feasibility and preliminary effects on exercise and dietary habits | Salmoirago-Blotcher et al | 2018 | Exclusion reason: stage 1 - Led by external provider/specialist |
| Observing the Delivery of a Curriculum-Integrated Dance Programme Across Four New Zealand Primary Schools | Sharma et al | 2019 | Exclusion reason: stage 1 - Led by external provider/specialist |
| Evaluation of the Good Start Program: a healthy eating and physical activity intervention for Maori and Pacific Islander children living in Queensland, Australia | Mihrshahi et al | 2017 | Exclusion reason: stage 1 - Led by external provider/specialist |
| Efficacy of the health promotion model-based intervention in enhancing the health responsibility of middle school female student: A randomized controlled trial | Dawood et al | 2019 | Exclusion reason: stage 1 - Led by external provider/specialist |
| Effects of an empowerment-based health-promotion school intervention on physical activity and sedentary time among adolescents in a multicultural area | Froberg et al | 2018 | Exclusion reason: stage 1 - Led by external provider/specialist |
| Effects of a Three-Tiered Intervention Model on Physical Activity and Fitness Levels of Elementary School Children | Dauenhauer et al | 2016 | Exclusion reason: stage 1 - Led by external provider/specialist |
| Effects of a structured recess intervention on physical activity levels, cardiorespiratory fitness, and anthropometric characteristics in primary school children | Casolo et al | 2019 | Exclusion reason: stage 1 - Led by external provider/specialist |
| Effects of a Physical Education Supportive Curriculum and Technological Devices on Physical Activity | Clapham et al | 2015 | Exclusion reason: stage 1 - Led by external provider/specialist |
| Does the Healthy Body Image program improve lifestyle habits among high school students? A randomized controlled trial with 12-month follow-up | Sundgot-Borgen et al | 2019 | Exclusion reason: stage 1 - Led by external provider/specialist |
| Assessing the impact of a physical education project based on games approach on the actual motor competence of primary school children | Sgro et al | 2019 | Exclusion reason: stage 1 - Led by external provider/specialist |
| Adolescent girls' physical activity, fitness and psychological well-being during a health club physical education approach | McNamee et al | 2017 | Exclusion reason: stage 1 - Led by external provider/specialist |
| Outcomes and process evaluation of a programme integrating physical activity into the primary school mathematics curriculum: The EASY Minds pilot randomised controlled trial | Riley et al | 2015 | Exclusion reason: stage 1 - Led by external provider/specialist |
| Outcomes of a four-year specialist-taught physical education program on physical activity: A cluster randomized controlled trial, the LOOK study | Telford et al | 2016 | Exclusion reason: stage 1 - Led by external provider/specialist |
| Elementary student and teacher perceptions of a mindfulness and yoga-based program in school: A qualitative evaluation | Reindl et al | 2020 | Exclusion reason: stage 1 - Led by external provider/specialist |
| Exploring Gender Differences within Forest Schools as a Physical Activity Intervention | Trapasso et al | 2018 | Exclusion reason: stage 1 - Led by external provider/specialist |
| Lessons Learned: A Strategic Alliance to Improve Elementary Physical Education in an Urban School District | Thompson et al | 2015 | Exclusion reason: stage 1 - Led by external provider/specialist |
| Changes in sedentary and active lifestyle, diet quality and body composition nine months after an education program in Polish students aged 11-12 years: Report from the ABC of healthy eating study | Wadolowska et al | 2019 | Exclusion reason: stage 1 - Led by external provider/specialist |
| 'A really good balance': Thematic analysis of stakeholders' views on classroom- and games-based positive choices interventions for primary school children | McCullogh et al | 2019 | Exclusion reason: stage 1 - Led by external provider/specialist |
| A non-equivalent group pilot trial of a school-based physical activity and fitness intervention for 10-11 year old english children: born to move | Fairclough et al | 2016 | Exclusion reason: stage 1 - Led by external provider/specialist |
| A clustered randomized controlled trial to determine impacts of the Harvest of the Month program | LaChausse et al | 2017 | Exclusion reason: stage 1 - Led by external provider/specialist |
| Fuel for Fun: a cluster-randomized controlled study of cooking skills, eating behaviors, and physical activity of 4th graders and their families | Cunningham-Sabo et al | 2016 | Exclusion reason: stage 1 - Led by external provider/specialist |
| 'Project Spraoi': A randomized control trial to improve nutrition and physical activity in school children | Coppinger et al | 2016 | Exclusion reason: stage 1 - Led by external provider/specialist |
| Positive Effects of Promoting Physical Activity and Balanced Diets in a Primary School Setting with a High Proportion of Migrant School Children | Weber et al | 2017 | Exclusion reason: stage 1 - Led by external provider/specialist |
| Physical activity and situational interest in mobile technology integrated physical education: A preliminary study | Xihe et al | 2016 | Exclusion reason: stage 1 - Led by external provider/specialist |
| Influence of sport education on high school students' motivational response: A gender perspective | Burgueño et al | 2020 | Exclusion reason: stage 1 - Led by external provider/specialist |
| A Community-Based Participatory Research Approach for Preventing Childhood Obesity: The Communities and Schools Together Project | Johnson-Shelton et al | 2015 | Exclusion reason: stage 1 - Led by external provider/specialist |
| A Nurse-Led and Teacher-Assisted Adolescent Healthy Weight Program to Improve Health Behaviors in the School Setting | Dupart et al | 2019 | Exclusion reason: stage 1 - Led by external provider/specialist |
| Intervention of childhood and adolescents obesity in Shantou city | Guo et al | 2015 | Exclusion reason: stage 1 - Led by external provider/specialist |
| The CHIRPY DRAGON intervention in preventing obesity in Chinese primaryschool-aged children: A cluster-randomised controlled trial | Li et al | 2019 | Exclusion reason: stage 1 - Led by external provider/specialist |
| Effect of a 12-Week Physical Activity Program on Gross Motor Skills in Children | Burns et al | 2017 | Exclusion reason: stage 1 - Led by external provider/specialist |
| Participants' physical activity levels and evaluations of a school sport programme in Papua New Guinea | Hanrahan et al | 2019 | Exclusion reason: stage 1 - Led by external provider/specialist |
| Effects of Moderate Interval Training on Heart Rate Variability among Primary School Children | Ketelhut et al | 2017 | Exclusion reason: stage 1 - Led by external provider/specialist |
| Effects of a school based intervention on children's physical activity and healthy eating: A mixed methods study | Khan et al | 2019 | Exclusion reason: stage 1 - Led by external provider/specialist |
| Empowering aspects for healthy food and physical activity habits: adolescents' experiences of a school-based intervention in a disadvantaged urban community | Holmberg et al | 2018 | Exclusion reason: stage 1 - Led by external provider/specialist |
| The Impact of Playworks on Students' Physical Activity by Race/Ethnicity: Findings from a Randomized Controlled Trial | James-Burdumy et al | 2016 | Exclusion reason: stage 1 - Led by external provider/specialist |
| Multi-teaching styles approach and active reflection: Effectiveness in improving fitness level, motor competence, enjoyment, amount of physical activity, and effects on the perception of physical education lessons in primary school children | Invernizzi et al | 2019 | Exclusion reason: stage 1 - Led by external provider/specialist |
| Effects of a strategy for the promotion of physical activity in students from Bogotá | Gutiérrez-Martínez et al | 2018 | Exclusion reason: stage 1 - Led by external provider/specialist |
| The use of information communication technology in teachers' practical training in the framework of physical education | Majerič et al | 2019 | Exclusion reason: stage 1 - Led by external provider/specialist |
| Effects of combined physical education and nutritional programs on schoolchildren's healthy habits | Gallotta et al | 2016 | Exclusion reason: stage 1 - Led by external provider/specialist |
| Effect of a school-based intervention on nutritional knowledge and habits of low-socioeconomic school children in Israel: A cluster-randomized controlled trial | Kaufman-Shriqui et al | 2016 | Exclusion reason: stage 1 - Led by external provider/specialist |
| Physical fitness of school age children post-implementation of an educational intervention to prevent childhood obesity in Morelos, Mexico. [Spanish] | Gatica-Dominguez et al | 2019 | Exclusion reason: stage 1 - Led by external provider/specialist |
| Health education: Effects on classroom climate and physical activity | Efstathiou et al | 2016 | Exclusion reason: stage 1 - Led by external provider/specialist |
| The effectiveness of the COPE healthy lifestyles TEEN program: a school-based intervention in middle school adolescents with 12-month follow-up | Ardic et al | 2017 | Exclusion reason: stage 1 - Led by external provider/specialist |
| The Effects of the Physical Best Health-Related Fitness Curriculum on Physical Activity Levels of Primary-Aged Physical Education Students | Deutsch et al | 2019 | Exclusion reason: stage 1 - Led by external provider/specialist |
| Mental training can improve physical activity behavior in adolescent girls | Najafabadi et al | 2017 | Exclusion reason: stage 1 - Led by external provider/specialist |
| Recess environment and curriculum intervention on children's physical activity: IPlay | Nigg et al | 2019 | Exclusion reason: stage 1 - Led by external provider/specialist |
| Energy Balance 4 Kids with Play: Results from a Two-Year Cluster-Randomized Trial | Madsen et al | 2015 | Exclusion reason: stage 1 - Led by external provider/specialist |
| Newham's Every Child a Sports Person (NECaSP): A Summative Process Evaluation of a School- and Community- Based Intervention in East London, United Kingdom | Curry et al | 2016 | Exclusion reason: stage 1 - Led by external provider/specialist |
| An active play intervention to improve physical activity and fundamental movement skills in children of low socioeconomic status: Feasibility cluster randomised controlled trial | Johnstone et al | 2019 | Exclusion reason: stage 1 - Led by external provider/specialist |
| Impact and Moderating Variables of an Intervention Promoting Physical Activity Among Children: Results From a Pilot Study | Gourlan et al | 2018 | Exclusion reason: stage 1 - Led by external provider/specialist |
| The effects of persuasive communication and planning on intentions to be more physically active and on physical activity behaviour among low-active adolescents | Tessier et al | 2015 | Exclusion reason: stage 1 - Led by external provider/specialist |
| Psychologically informed physical fitness practice in schools: A field experiment | Vazou et al | 2019 | Exclusion reason: stage 1 - Led by external provider/specialist |
| Using a co-creational approach to develop, implement and evaluate an intervention to promote physical activity in adolescent girls from vocational and technical schools: A case control study | Verloigne et al | 2017 | Exclusion reason: stage 1 - Led by external provider/specialist |
| Impact of combined theory-based intervention on psychological effects and physical activity among chinese adolescents | Zhang et al | 2020 | Exclusion reason: stage 1 - Led by external provider/specialist |
| HybridPLAY: A New Technology to Foster Outdoors Physical Activity, Verbal Communication and Teamwork | Diaz et al | 2016 | Exclusion reason: stage 1 - Led by external provider/specialist |
| Effectiveness of solution-focused brief counselling in dealing with problems with physical education among senior students | Indriūnienė et al | 2017 | Exclusion reason: stage 1 - Led by external provider/specialist |
| Does School-Based Health Promotion Affect Physical Activity on Weekends? And, Does It Reach Those Students Most in Need of Health Promotion? | Bastian et al | 2015 | Exclusion reason: stage 1 - Led by external provider/specialist |
| Targeting bone and fat with novel exercise for peripubertal boys: the CAPO kids trial | Nogueira et al | 2015 | Exclusion reason: stage 1 - Led by external provider/specialist |
| Using the Step it UP! Game to increase physical activity during physical-education classes | Normand et al | 2020 | Exclusion reason: stage 1 - Led by external provider/specialist |
| Evaluating Mailed Motivational, Individually Tailored Postcard Boosters for Promoting Girls' Postintervention Moderate-to-Vigorous Physical Activity | Bakhoya et al | 2016 | Exclusion reason: stage 1 - Not a school-based intervention |
| Anthropology in the design of preventive behavioral health programs for children and families living in disadvantaged neighborhoods | Azevedo et al | 2015 | Exclusion reason: stage 1 - Not a school-based intervention |
| "The Stomp and Catch Was Too Easy!" Children's and Teachers' Perceptions of Inclusive High and Low Autonomy Motor Skills Instruction | Buchanan et al | 2019 | Exclusion reason: stage 1 - Not school-based |
| Classroom-Based Physical Activity: Minimizing Disparities in School-Day Physical Activity Among Elementary School Students | Calvert et al | 2018 | Exclusion reason: stage 1 - Not school-based |
| Mixed-Methods Evaluation of a Healthy Exercise, Eating, and Lifestyle Program for Primary Schools | Cochrane et al | 2017 | Exclusion reason: stage 1 - Not school-based |
| Promoting Healthy Diet, Physical Activity, and Life-Skills in High School Athletes: Results from the WAVE Ripples for Change Childhood Obesity Prevention Two-Year Intervention | Meng et al | 2018 | Exclusion reason: stage 1 - Not school-based |
| Student obesity prevalence and behavioral outcomes for the massachusetts childhood obesity research demonstration project | Franckle et al | 2017 | Exclusion reason: stage 1 - Not school-based |
| From surveillance to intervention: Overview and baseline findings for the active city of Liverpool active schools and sportslinx (A-CLASS) project | McWhannell et al | 2018 | Exclusion reason: stage 1 - Not school-based |
| Using outdoor adventure to enhance intrinsic motivation and engagement in science and physical activity: An exploratory study | Mackenzie et al | 2018 | Exclusion reason: stage 1 - Not school-based |
| Effects of the Girls on the Move randomized trial on adiposity and aerobic performance (secondary outcomes) in low-income adolescent girls | Pfeiffer et al | 2019 | Exclusion reason: stage 1 - Not school-based |
| Changes in developmental assets and physical activity frequency among 3rd-5th grade girls participating in a girl-focused sport-based positive youth development program | DeBate et al | 2016 | Exclusion reason: stage 1 - Not school-based |
| Promoting Physical Activity and Science Learning in an Outdoor Education Program | Finn et al | 2018 | Exclusion reason: stage 1 - Not school-based |
| WAVE~ripples for change obesity two-year intervention in high school soccer players: Process evaluation, best practices, and youth engagement | Meng et al | 2018 | Exclusion reason: stage 1 - Not school-based |
| Increases in lifestyle activities as a result of experience Corps participation | Parisi et al | 2015 | Exclusion reason: stage 1 - Not school-based |
| Effect of augmented reality game Pokemon GO on cognitive performance and emotional intelligence in adolescent young | Ruiz-Ariza et al | 2018 | Exclusion reason: stage 1 - Not school-based |
| Decreasing sedentary behavior: Effects on academic performance, meta-cognition, and sleep | Pilcher et al | 2017 | Exclusion reason: stage 1 - Not school-based |
| Challenges and Facilitators to the Implementation of a Sport Education Season: The Voices of Teacher Candidates | Braga et al | 2017 | Exclusion reason: stage 1 - Not school-based |
| Effect of a Physical Education Training Program on the Physical Education Teaching Efficacy of Classroom Teacher Candidates | Unlu et al | 2019 | Exclusion reason: stage 1 - Not school-based |
| Influence of Visual and Auditory Stimuli on Exercise Intensity Among School-Age Children | Sandoval et al | 2019 | Exclusion reason: stage 1 - Not school-based |
| Preventing weight-related problems among adolescent girls: A cluster randomized trial comparing the Brazilian 'New Moves' program versus observation | Dunker et al | 2018 | Exclusion reason: stage 1 - Not school-based |
| Organization of domiciliary sports and physical training and recreation activities with children (the case of Irkutsk) | Abramovich et al | 2015 | Exclusion reason: stage 1 - Not school-based |
| A School- and Home-Based Intervention to Improve Adolescents' Physical Activity and Healthy Eating: A Pilot Study | Robbins et al | 2020 | Exclusion reason: stage 1 - Not school-based |
| A cluster randomized controlled trial of a positive physical activity intervention | Ho et al | 2020 | Exclusion reason: stage 1 - Not school-based |
| Active children through incentive vouchers - evaluation (ACTIVE): a mixed-method feasibility study | Christian et al | 2016 | Exclusion reason: stage 1 - Not school-based |
| Development of a Workplace Intervention for Child Care Staff: Caring and Reaching for Health's (CARE) Healthy Lifestyles Intervention | Arandia et al | 2020 | Exclusion reason: stage 1 - Not school-based |
| Longitudinal data from a school-based intervention - The ACORDA project. | Aires et al | 2015 | Exclusion reason: stage 1 - Not school-based |
| Foundations for Fitness: A Multi-Cohort Pediatric Weight Management Intervention | Morrison et al | 2018 | Exclusion reason: stage 1 - Not school-based |
| Lessons from a peer-led obesity prevention programme in English schools | Bell et al | 2017 | Exclusion reason: stage 1 - Not teacher-led |
| One- and two-year effects of the healthy primary school of the future on children's dietary and physical activity behaviours: A quasi-experimental study | Bartelink et al | 2019 | Exclusion reason: stage 1 - Not teacher-led |
| Modifying the classroom environment to increase standing and reduce sitting | Aminian et al | 2015 | Exclusion reason: stage 1 - Not teacher-led |
| The health Oriented pedagogical project (HOPP) - a controlled longitudinal school-based physical activity intervention program | Fredriksen et al | 2017 | Exclusion reason: stage 1 - Protocol paper |
| A cluster randomized control trial to assess the impact of active learning on child activity, attention control, and academic outcomes: The Texas I-CAN trial | Bartholomew et al | 2017 | Exclusion reason: stage 1 - Protocol paper |
| Evaluation overview for the Massachusetts Childhood Obesity Research Demonstration (MA-CORD) project | Davison et al | 2015 | Exclusion reason: stage 1 - Protocol paper |
| Effects of a multi-level intervention on the pattern of physical activity among in-school adolescents in Oyo state Nigeria: a cluster randomised trial | Oluwasanu et al | 2017 | Exclusion reason: stage 1 - Protocol paper |
| Design and methods for "Commit to Get Fit" - A pilot study of a school-based mindfulness intervention to promote healthy diet and physical activity among adolescents | Salmoirago-Blotcher et al | 2015 | Exclusion reason: stage 1 - Protocol paper |
| Development of the 'Sigue la Huella' physical activity intervention for adolescents in Huesca, Spain | Murillo Pardo et al | 2019 | Exclusion reason: stage 1 - Protocol paper |
| Physical activity across the curriculum (PAAC3): Testing the application of technology delivered classroom physical activity breaks | Szabo-Reed et al | 2020 | Exclusion reason: stage 1 - Protocol paper |
| Background and evaluation design of a community-based health-promoting school intervention: Fit Lifestyle at School and at Home (FLASH) | van Dongen et al | 2019 | Exclusion reason: stage 1 - Protocol paper |
| A cluster randomised controlled trial to evaluate the effectiveness and cost-effectiveness of the GoActive intervention to increase physical activity among adolescents aged 13-14 years | Brown et al | 2017 | Exclusion reason: stage 1 - Protocol paper |
| Design, randomization and methodology of the TriAtiva Program to reduce obesity in school children in Southern Brazil | Friedrich et al | 2015 | Exclusion reason: stage 1 - Protocol paper |
| One Step Forward: Development of a Program Promoting Active School Transportation | Lindqvist et al | 2018 | Exclusion reason: stage 1 - Protocol paper |
| How To Increase The Daily Physical Activity During The School Day? Example Of An Interdisciplinary Project Between Physical Education And Mathematics | Carriedo et al | 2019 | Exclusion reason: stage 1 - Protocol paper |
| Improving the well-being of children and youths: a randomized multicomponent, school-based, physical activity intervention | Smedegaard et al | 2016 | Exclusion reason: stage 1 - Protocol paper |
| Activating schoolyards: study design of a quasi-experimental schoolyard intervention study | Andersen et al | 2015 | Exclusion reason: stage 1 - Protocol paper |
| A cluster-randomised controlled trial to promote physical activity in adolescents: the Raising Awareness of Physical Activity (RAW-PA) Study | Ridgers et al | 2017 | Exclusion reason: stage 1 - Protocol paper |
| Evaluation of a comprehensive school physical activity program: Be a Champion! | Singletary et al | 2019 | Exclusion reason: stage 1 - Protocol paper |
| Study design and protocol for a mixed methods evaluation of an intervention to reduce and break up sitting time in primary school classrooms in the UK: The CLASS PAL (Physically Active Learning) Programme | Routen et al | 2017 | Exclusion reason: stage 1 - Protocol paper |
| Effectiveness of a universal parental support programme to promote healthy dietary habits and physical activity and to prevent overweight and obesity in 6-year-old children: The healthy school start study, a cluster-randomised controlled trial | Nyberg et al | 2015 | Exclusion reason: stage 1 - Special population (e.g. children with overweight, obesity or ASD) |
| Longitudinal levels and bouts of objectively measured sedentary time among young Australian children in the HAPPY study | Carson et al | 2016 | Exclusion reason: stage 1 - Special population (e.g. children with overweight, obesity or ASD) |
| Juara Sihat: Assessing the sustained impact of a school-based obesity intervention | Mok et al | 2018 | Exclusion reason: stage 1 - Special population (e.g. children with overweight, obesity or ASD) |
| Promoting physical activity and improving dietary quality of Singaporean adolescents: Effectiveness of a school-based fitness and wellness program | Loong et al | 2018 | Exclusion reason: stage 1 - Special population (e.g. children with overweight, obesity or ASD) |
| Effectiveness of structured physical activity intervention on physical activity and body mass index among adolescents - a pilot study | Sumathy et al | 2018 | Exclusion reason: stage 1 - Special population (e.g. children with overweight, obesity or ASD) |
| Holistic Health Promotion for Adolescent Girls in an Alternative School Setting: Lessons Learned | Saltzman et al | 2015 | Exclusion reason: stage 1 - Special population (e.g. children with overweight, obesity or ASD) |
| Effects of 28 weeks of high-intensity interval training during physical education classes on cardiometabolic risk factors in Chilean schoolchildren: a pilot trial | Delgado-Floody et al | 2018 | Exclusion reason: stage 1 - Special population (e.g. children with overweight, obesity or ASD) |
| Vocational Education Students' Perceptions About The Effects Of A Positive Development Program (Responsibility Hellison's Model) | Caballero et al | 2015 | Exclusion reason: stage 1 - Special population (e.g. children with overweight, obesity or ASD) |
| Implementation and evaluation of a collaborative gymnastic strategy | Ávalos Ramos et al | 2019 | Exclusion reason: stage 1 - Special population (e.g. children with overweight, obesity or ASD) |
| One size does not fit all-qualitative process evaluation of the Healthy School Start parental support programme to prevent overweight and obesity among children in disadvantaged areas in Sweden | Norman et al | 2016 | Exclusion reason: stage 1 - Special population (e.g. children with overweight, obesity or ASD) |
| Reducing children's classroom sitting time using sit-to-stand desks: findings from pilot studies in UK and Australian primary schools | Clemes et al | 2016 | Exclusion reason: stage 1 - Teacher has little/no role e.g. structural changes |
| Trends in sedentary behavior, physical activity, and motivation during a classroom-based active video game program | Fu et al | 2019 | Exclusion reason: stage 1 - Teacher has little/no role e.g. structural changes |
| The Effects of Active Videogame Feedback and Practicing Experience on Children's Physical Activity Intensity and Enjoyment | Chen et al | 2017 | Exclusion reason: stage 1 - Teacher has little/no role e.g. structural changes |
| Effect of the Shaping Healthy Choices Program, a Multicomponent, School-Based Nutrition Intervention, on Physical Activity Intensity | Fetter et al | 2018 | Exclusion reason: stage 1 - Teacher has little/no role e.g. structural changes |
| Low-cost and Scalable Classroom Equipment to Promote Physical Activity and Improve Education | McCrady-Spitzer et al | 2015 | Exclusion reason: stage 1 - Teacher has little/no role e.g. structural changes |
| Standing desks in a grade 4 classroom over the full school year | Parry et al | 2019 | Exclusion reason: stage 1 - Teacher has little/no role e.g. structural changes |
| Active Living: Development and quasi-experimental evaluation of a school-centered physical activity intervention for primary school children Energy balance-related behaviours | Van Kann et al | 2015 | Exclusion reason: stage 1 - Teacher has little/no role e.g. structural changes |
| Self-directed learning: An innovative strategy for sport and physical education | Toto et al | 2019 | Exclusion reason: stage 1 - Teacher has little/no role e.g. structural changes |
| The Impact and Feasibility of Introducing Height-Adjustable Desks on Adolescents' Sitting in a Secondary School Classroom | Sudholz et al | 2016 | Exclusion reason: stage 1 - Teacher has little/no role e.g. structural changes |
| A feasibility study of 'The StepSmart Challenge' to promote physical activity in adolescents | Corepal et al | 2019 | Exclusion reason: stage 1 - Teacher has little/no role e.g. structural changes |
| A Multicomponent Schoolyard Intervention Targeting Children's Recess Physical Activity and Sedentary Behavior: Effects After One Year | Van Kann et al | 2016 | Exclusion reason: stage 1 - Teacher has little/no role e.g. structural changes |
| A process evaluation of the PLAN-A intervention (Peer-Led physical Activity iNtervention for Adolescent girls) | Sebire et al | 2019 | Exclusion reason: stage 1 - Teacher has little/no role e.g. structural changes |
| The Impact of Classroom Physical Activity Breaks on Middle School Students' Health-Related Fitness: An Xbox One Kinetic Delivered 4-Week Randomized Controlled Trial | Yli-Piipari et al | 2016 | Exclusion reason: stage 1 - Teacher has little/no role e.g. structural changes |
| Effects of school-based exergaming on urban children's physical activity and cardiorespiratory fitness: A quasi-experimental study | Ye et al | 2019 | Exclusion reason: stage 1 - Teacher has little/no role e.g. structural changes |
| Do stand-biased desks in the classroom change school-time activity and sedentary behavior? | Swartz et al | 2019 | Exclusion reason: stage 1 - Teacher has little/no role e.g. structural changes |
| Exploring the acceptability and usability of a novel social innovation to encourage physical activity: The iStep prototype | Grindell et al | 2019 | Exclusion reason: stage 1 - Teacher has little/no role e.g. structural changes |
| Impact of a pedometer-based goal-setting intervention on children’s motivation, motor competence, and physical activity in physical education | Gu et al | 2018 | Exclusion reason: stage 1 - Teacher has little/no role e.g. structural changes |
| Does a classroom standing desk intervention modify standing and sitting behaviour and musculoskeletal symptoms during school time and physical activity during waking time? | Ee et al | 2018 | Exclusion reason: stage 1 - Teacher has little/no role e.g. structural changes |
| The effect of playground-and nature-based playtime interventions on physical activity and self-esteem in UK school children | Barton et al | 2015 | Exclusion reason: stage 1 - Teacher has little/no role e.g. structural changes |
| Do they need goals or support? A report from a goal-setting intervention using physical activity monitors in youth | Bronikowski et al | 2016 | Exclusion reason: stage 1 - Teacher has little/no role e.g. structural changes |
| e-Gibalec: Mobile application to monitor and encourage physical activity in schoolchildren | Janko et al | 2017 | Exclusion reason: stage 1 - Teacher has little/no role e.g. structural changes |
| Changes in physical activity and sedentary time in the Finnish Schools on the Move program: a quasi-experimental study | Haapala et al | 2017 | Exclusion reason: stage 1 - Teacher has little/no role e.g. structural changes |
| Feasibility and efficacy of the Great Leaders Active StudentS (GLASS) program on children's physical activity and object control skill competency: A non-randomised trial | Nathan et al | 2017 | Exclusion reason: stage 1 - Teacher has little/no role e.g. structural changes |
| The evaluation of outdoor learning activities in primary school | Henrietta et al | 2019 | Exclusion reason: stage 1 - Wrong intervention (e.g. social inclusion) |
| Studying the Effectiveness of Physical Education in the Secondary School (by the Example of Kazakhstan) | Botagariyev et al | 2016 | Exclusion reason: stage 1 - Wrong intervention (e.g. social inclusion) |
| The effect of a content knowledge teacher professional workshop on enacted pedagogical content knowledge and student learning in a throwing unit | Chang et al | 2020 | Exclusion reason: stage 1 - Wrong intervention (e.g. social inclusion) |
| A cluster-randomized controlled trial to improve student experiences in physical education: Results of a student-centered learning intervention with high school teachers | Bechter et al | 2019 | Exclusion reason: stage 1 - Wrong intervention (e.g. social inclusion) |
| Monitoring and evaluation of sports load for primary and middle school students | Luo et al | 2018 | Exclusion reason: stage 1 - Wrong intervention (e.g. social inclusion) |
| Effects of a School-Based Social-Emotional and Character Development Program on Health Behaviors: A Matched-Pair, Cluster-Randomized Controlled Trial | Bavarian et al | 2016 | Exclusion reason: stage 1 - Wrong intervention (e.g. social inclusion) |
| Effects of physical activity and breaks on mathematics engagement in adolescents | Owen et al | 2018 | Exclusion reason: stage 1 - Wrong intervention (e.g. social inclusion) |
| Health promotion initiatives at school related to overweight, insulin resistance, hypertension and dyslipidemia in adolescents: a cross-sectional study in Recife, Brazil | de Assuncao Bezerra et al | 2018 | Exclusion reason: stage 1 - Wrong intervention (e.g. social inclusion) |
| Implementing differentiated instruction approach in physical training and sports lesson | Özbal et al | 2019 | Exclusion reason: stage 1 - Wrong intervention (e.g. social inclusion) |
| Effect of applied health-oriented exercises in physical and sport education on musculoskeletal system of female students | Bendíková et al | 2018 | Exclusion reason: stage 1 - Wrong intervention (e.g. social inclusion) |
| Promoting schoolchildren's self-esteem in physical education: testing the effectiveness of a five-month teacher training | Rubeli et al | 2020 | Exclusion reason: stage 1 - Wrong intervention (e.g. social inclusion) |
| A successful nationwide implementation of the 'FIFA 11 for Health' programme in Brazilian elementary schools | Fuller et al | 2015 | Exclusion reason: stage 1 - Wrong intervention (e.g. social inclusion) |
| An evaluation of the developed physical education curriculum from the viewpoint of teachers at the governorate of Irbid, Jordan | Deity et al | 2019 | Exclusion reason: stage 1 - Wrong intervention (e.g. social inclusion) |
| An examination of Greek physical educators’ implementation and perceptions of Spectrum teaching styles | Syrmpas et al | 2016 | Exclusion reason: stage 1 - Wrong intervention (e.g. social inclusion) |
| Quality of local school wellness policies for physical activity and resultant implementation in Pennsylvania schools | Francis et al | 2018 | Exclusion reason: stage 1 - Wrong intervention (e.g. social inclusion) |
| Perceptions and measurement of playtime physical activity in English primary school children: The influence of socioeconomic status | McWhannell et al | 2019 | Exclusion reason: stage 1 - Wrong intervention (e.g. social inclusion) |
| Changes in weight status, quality of life and behaviours of South Australian primary school children: results from the Obesity Prevention and Lifestyle (OPAL) community intervention program | Bell et al | 2019 | Exclusion reason: stage 1 - Wrong intervention (e.g. social inclusion) |
| Benefits of Strength and Skill-based Training During Primary School Physical Education | Faigenbaum et al | 2015 | Exclusion reason: stage 1 - Wrong intervention (e.g. social inclusion) |
| Understanding implementation and change in complex interventions. From single- to multi-methodological research on the promotion of youths’ participation in physical education | Agergaard et al | 2018 | Exclusion reason: stage 1 - Wrong intervention (e.g. social inclusion) |
| A school-based intervention improved dietary intake outcomes and reduced waist circumference in adolescents: a cluster randomized controlled trial | Ochoa-Aviles et al | 2017 | Exclusion reason: stage 1 - Wrong intervention (e.g. social inclusion) |
| Advancing School and Community Engagement Now for Disease Prevention (ASCEND): A Quasi-experimental Trial of School-Based Interventions to Prevent Childhood Obesity | Treu et al | 2017 | Exclusion reason: stage 1 - Wrong intervention (e.g. social inclusion) |
| School-based physical education: Physical activity and implementation barriers in Vietnamese elementary schools | To et al | 2020 | Exclusion reason: stage 1 - Wrong intervention (e.g. social inclusion) |
| A school educational intervention based on a serious game to promote a healthy lifestyle | Marchetti et al | 2018 | Exclusion reason: stage 1 - Wrong intervention (e.g. social inclusion) |
| High-intensity training enhances executive function in children in a randomized, placebo-controlled trial | Moreau et al | 2017 | Exclusion reason: stage 1 - Wrong intervention (e.g. social inclusion) |
| Relationship of Physical Education Curriculum Implementation and Mathematics Achievement in Chinese Youth | Wang et al | 2019 | Exclusion reason: stage 1 - Wrong intervention (e.g. social inclusion) |
| The Reality of Sustaining Community-Based Sport and Physical Activity Programs to Enhance the Development of Underserved Youth: Challenges and Potential Strategies | Whitley et al | 2015 | Exclusion reason: stage 1 - Wrong intervention (e.g. social inclusion) |
| Active School Lesson Breaks Increase Daily Vigorous Physical Activity, but Not Daily Moderate to Vigorous Physical Activity in Elementary School Boys | Wilson et al | 2017 | Exclusion reason: stage 1 - Wrong intervention (e.g. social inclusion) |
| "Now we help ourselves more": Team-teachinq and social classroom climate. Experience with Sport Education | Calderón et al | 2016 | Exclusion reason: stage 1 - Wrong intervention (e.g. social inclusion) |
| A model for promoting physical activity among rural South African adolescent girls | Kinsman et al | 2015 | Exclusion reason: stage 1 - Wrong intervention (e.g. social inclusion) |
| Cluster-randomized, controlled evaluation of a teacher led multi factorial school based back education program for 10 to 12-year old children | Dullien et al | 2018 | Exclusion reason: stage 1 - Wrong intervention (e.g. social inclusion) |
| Development and optimisation of an in-service teacher training programme on motivational assessment in physical education | Slingerland et al | 2017 | Exclusion reason: stage 1 - Wrong intervention (e.g. social inclusion) |
| The Role of School in Helping Children and Adolescents Reach the Physical Activity Recommendations: The UP&DOWN Study | Grao-Cruces et al | 2019 | Exclusion reason: stage 1 - Wrong intervention (e.g. social inclusion) |
| Development of coordination capacities through dancing among primary school children | Nanu et al | 2018 | Exclusion reason: stage 1 - Wrong intervention (e.g. social inclusion) |
| Superheroes and education for leisure: description of a pedagogical proposal in Physical Education classes | Rossi Filho et al | 2019 | Exclusion reason: stage 1 - Wrong intervention (e.g. social inclusion) |
| Teens Implementing a Childhood Obesity Prevention Program in the Community: Feasibility and Perceptions of a Partnership with HSTA and iCook 4-H | Hagedorn et al | 2018 | Exclusion reason: stage 1 - Wrong intervention (e.g. social inclusion) |
| The implementation of health education in Subcarpathian schools after the introduction of the new core curriculum as viewed by PE teachers | Zadarko-Domaradzka et al | 2015 | Exclusion reason: stage 1 - Wrong intervention (e.g. social inclusion) |
| The relationship between physical activity and content of the physical education classes in 11-12 years old lithuanian schoolchildren. The pilot study | Emeljanovas et al | 2015 | Exclusion reason: stage 1 - Wrong intervention (e.g. social inclusion) |
| Innovation with change: developing a community of practice to help teachers move beyond the ‘honeymoon’ of pedagogical renovation | Goodyear et al | 2015 | Exclusion reason: stage 1 - Wrong intervention (e.g. social inclusion) |
| A classroom-based intervention to help teachers decrease students' amotivation | Cheon et al | 2015 | Exclusion reason: stage 1 - Wrong intervention (e.g. social inclusion) |
| Positive experiences in physical education through teacher intervention in the teaching unit futsal | Abos Catalan et al | 2015 | Exclusion reason: stage 1 - Wrong intervention (e.g. social inclusion) |
| Implementation of Kenyan comprehensive school health program: improvement and association with students' academic attainment | Akiyama et al | 2020 | Exclusion reason: stage 1 - Wrong intervention (e.g. social inclusion) |
| A Professional Development Program to Enhance Primary School Teachers' Knowledge and Operationalization of Physical Literacy | Edwards et al | 2019 | Exclusion reason: stage 1 - Wrong intervention (e.g. social inclusion) |
| Effect of educational intervention based on Self-Efficacy on preventive behaviors of overweight and obesity among secondary-school female students in Mashhad. [Persian] | Hejazi et al | 2017 | Exclusion reason: stage 1 - Wrong intervention (e.g. social inclusion) |
| The Flipped Classroom Through The Smartphone: Effects Of Its Experimentation In High School Physical Education | Gomez Garcia et al | 2015 | Exclusion reason: stage 1 - Wrong intervention (e.g. social inclusion) |
| Teaching-Learning In The Physical Education Classs | Garcia Pena et al | 2018 | Exclusion reason: stage 1 - Wrong intervention (e.g. social inclusion) |
| An Intervention to Improve Teachers' Interpersonally Involving Instructional Practices in High School Physical Education: Implications for Student Relatedness Support and In-Class Experiences | Sparks et al | 2017 | Exclusion reason: stage 1 - Wrong intervention (e.g. social inclusion) |
| Teacher And Administrative Opinions On Physical Education Course Efficiency In Ib-Pyp Applied Schools In Ankara | Bulut et al | 2018 | Exclusion reason: stage 1 - Wrong intervention (e.g. social inclusion) |
| Improving children's physical self-perception through a school-based physical activity intervention: The Move for Well-being in School study | Christiansen et al | 2018 | Exclusion reason: stage 1 - Wrong intervention (e.g. social inclusion) |
| The Co-Construction of Cooperative Learning in Physical Education With Elementary Classroom Teachers | Dyson et al | 2016 | Exclusion reason: stage 1 - Wrong intervention (e.g. social inclusion) |
| Aerobic Gymnastics On Kangoo-Jumps Boots And Its Impact On Students' Fitness | Germina et al | 2015 | Exclusion reason: stage 1 - Wrong intervention (e.g. social inclusion) |
| Assessment of the Sport Schools program: Body composition, physical activity and cardiorespiratory fitness in adolescents | Grao-Cruces et al | 2015 | Exclusion reason: stage 1 - Wrong intervention (e.g. social inclusion) |
| A school-based physical activity intervention to promote motor proficiency among adolescent girls: A randomized controlled trial | Hajihosseini et al | 2016 | Exclusion reason: stage 1 - Wrong intervention (e.g. social inclusion) |
| Acute effects of reducing sitting time in adolescents: a randomized cross-over study | Penning et al | 2017 | Exclusion reason: stage 1 - Wrong intervention (e.g. social inclusion) |
| One-year changes in physical activity and sedentary behavior among adolescents: The Croatian Physical Activity in Adolescence Longitudinal Study (CRO-PALS) | Stefan et al | 2018 | Exclusion reason: stage 1 - Wrong intervention (e.g. social inclusion) |
| Effects of a Participatory School-Based Intervention on Students' Health-Related Knowledge and Understanding | Strobl et al | 2020 | Exclusion reason: stage 1 - Wrong intervention (e.g. social inclusion) |
| Physical activity in physical education: are longer lessons better? | Smith et al | 2015 | Exclusion reason: stage 1 - Wrong intervention (e.g. social inclusion) |
| Investigating Children's Short-Term Responses to Imposed or Restricted Physical Activity | Ridgers et al | 2018 | Exclusion reason: stage 1 - Wrong intervention (e.g. social inclusion) |
| Mapping physical education teachers’ professional learning and impacts on pupil learning in a community of practice in South Korea | Yoon et al | 2017 | Exclusion reason: stage 1 - Wrong intervention (e.g. social inclusion) |
| Manipulation of the task constraints in Physical Education: A proposal from nonlinear pedagogy | Arias et al | 2016 | Exclusion reason: stage 1 - Wrong intervention (e.g. social inclusion) |
| Assessing the Implementation Fidelity of a School-Based Teaching Personal and Social Responsibility Program in Physical Education and Other Subject Areas | Escartí et al | 2018 | Exclusion reason: stage 1 - Wrong intervention (e.g. social inclusion) |
| Off-Balance: The Integration of Physical Education Content Learning and Irish Language Learning in English-Medium Primary Schools in Ireland | Ní Chróinín et al | 2016 | Exclusion reason: stage 1 - Wrong intervention (e.g. social inclusion) |
| 'Maths on the move': Effectiveness of physically-active lessons for learning maths and increasing physical activity in primary school students | Vetter et al | 2020 | Exclusion reason: stage 1 - Wrong outcome(s) |
| Relationship Between Teacher Fidelity and Physical Education Student Outcomes | Loflin et al | 2015 | Exclusion reason: stage 1 - Wrong outcome(s) |
| The effectiveness of an Autonomy-Supportive Teaching Structure in Physical Education. | How Yew et al | 2016 | Exclusion reason: stage 1 - Wrong outcome(s) |
| Building healthy communities: A comprehensive school health program to prevent obesity in elementary schools | Centeio et al | 2018 | Exclusion reason: stage 1 - Wrong outcome(s) |
| Active Learning Increases Children's Physical Activity across Demographic Subgroups | Bartholomew et al | 2018 | Exclusion reason: stage 1 - Wrong outcome(s) |
| School-based intervention on healthy behaviour among Ecuadorian adolescents: effect of a cluster-randomized controlled trial on screen-time | Andrade et al | 2015 | Exclusion reason: stage 1 - Wrong outcome(s) |
| Effectiveness and Cost-Effectiveness of daily School Sport in the Primary School - Project "fit for pisa" | Liersch et al | 2015 | Exclusion reason: stage 1 - Wrong outcome(s) |
| Implementation practices of the Rugby-5 into the physical education of schoolchildren 12-13 years old using information technology | Ashanin et al | 2018 | Exclusion reason: stage 1 - Wrong outcome(s) |
| Program Reach and Implementation Feasibility of a Physical Activity School Health Program: A Qualitative Study of Teachers' Perception | Guldager et al | 2019 | Exclusion reason: stage 1 - Wrong outcome(s) |
| Team-teaching in physical education for promoting coordinative motor skills in children: the more you invest the more you get | Bardaglio et al | 2015 | Exclusion reason: stage 1 - Wrong outcome(s) |
| The outcomes of health-promoting communities: Being active eating well initiative- A community-based obesity prevention intervention in Victoria, Australia | Bolton et al | 2017 | Exclusion reason: stage 1 - Wrong outcome(s) |
| Positive Impact on Physical Activity and Health Behaviour Changes of a 15-Week Family Focused Intervention Program: "juniors for Seniors" | Bronikowski et al | 2016 | Exclusion reason: stage 1 - Wrong outcome(s) |
| Supporting active school travel: A qualitative analysis of implementing a regional safe routes to school program | Buttazzoni et al | 2018 | Exclusion reason: stage 1 - Wrong outcome(s) |
| School-Based Obesity Intervention Associated with Three Year Decrease in Student Weight Status in a Low-Income School District | Cadzow et al | 2015 | Exclusion reason: stage 1 - Wrong outcome(s) |
| School Physical Activity Programming and Gross Motor Skills in Children | Burns et al | 2017 | Exclusion reason: stage 1 - Wrong outcome(s) |
| Satisfaction in the Physical Education classroom and intention to be physically active in Primary school children | Enríquez et al | 2020 | Exclusion reason: stage 1 - Wrong outcome(s) |
| Physical activity school intervention: context matters | Guldager et al | 2018 | Exclusion reason: stage 1 - Wrong outcome(s) |
| Promoting Healthy Lifestyle Behaviour through the Life-Orientation Curriculum: Teachers' Perceptions of the HealthKick Intervention | Hill et al | 2015 | Exclusion reason: stage 1 - Wrong outcome(s) |
| Using the critical incident technique for qualitative process evaluation of interventions: The example of the "Let's Move It" trial | Kostamo et al | 2019 | Exclusion reason: stage 1 - Wrong outcome(s) |
| Voices from Pupil Participation in the Health Promotion Intervention "Pulse for Learning and Health PuLH " in Primary and Middle School | Lindgren et al | 2019 | Exclusion reason: stage 1 - Wrong outcome(s) |
| A Preliminary Evaluation of a Cost-effective, In-class Physical Activity and Nutrition Education Intervention for 3rd through 6th Grade Students | Mouttapa et al | 2016 | Exclusion reason: stage 1 - Wrong outcome(s) |
| Implementing and Evaluating Environmental and Policy Interventions for Promoting Physical Activity in Rural Schools | Baker et al | 2017 | Exclusion reason: stage 1 - Wrong outcome(s) |
| Prospective effects of pedometer use and class competitions on physical activity in youth: A cluster-randomized controlled trial | Suchert et al | 2015 | Exclusion reason: stage 1 - Wrong outcome(s) |
| CDC childhood physical activity strategies fail to show sustained fitness impact in middle school children | Seibert et al | 2018 | Exclusion reason: stage 1 - Wrong outcome(s) |
| An intervention to promote physical activity in Mexican elementary school students: building public policy to prevent noncommunicable diseases | Polo-Oteyza et al | 2017 | Exclusion reason: stage 1 - Wrong outcome(s) |
| School-based systems change for obesity prevention in adolescents: outcomes of the Australian Capital Territory 'It's Your Move!' | Malakellis et al | 2017 | Exclusion reason: stage 1 - Wrong outcome(s) |
| A stepped-wedge implementation and evaluation of the healthy active peaceful playgrounds for youth (HAPPY) intervention | Dudley et al | 2018 | Exclusion reason: stage 1 - Wrong outcome(s) |
| Implementation of the HealthKick intervention in primary schools in low-income settings in the Western Cape Province, South Africa: a process evaluation | de Villiers et al | 2015 | Exclusion reason: stage 1 - Wrong outcome(s) |
| 'No one ever asked us': a feasibility study assessing the co-creation of a physical activity programme with adolescent girls | Corr et al | 2019 | Exclusion reason: stage 1 - Wrong outcome(s) |
| Initial experience with the Sport Education model in primary school first-grade. Students and teachers’ perceptions | de Ojeda et al | 2019 | Exclusion reason: stage 1 - Wrong outcome(s) |
| Evaluation of a pilot school-based physical activity challenge for primary students | Passmore et al | 2017 | Exclusion reason: stage 1 - Wrong outcome(s) |
| The Positive Effects of a Combined Program of Creative Dance and BrainDance on Health-Related Ouality of Life as Perceived by Primary School Students | Olga et al | 2018 | Exclusion reason: stage 1 - Wrong outcome(s) |
| Understanding the Importance of Context: A Qualitative Study of a Location-Based Exergame to Enhance School Childrens Physical Activity | Robertson et al | 2016 | Exclusion reason: stage 1 - Wrong outcome(s) |
| Playing Fair: The Contribution of High-Functioning Recess to Overall School Climate in Low-Income Elementary Schools | London et al | 2015 | Exclusion reason: stage 1 - Wrong outcome(s) |
| Effects of Generalization of Engagement in Parkour from Physical Education to Recess on Physical Activity | Coolkens et al | 2018 | Exclusion reason: stage 1 - Wrong outcome(s) |
| Bilingual physical education: the effects of CLIL on physical activity levels | Salvador-García et al | 2019 | Exclusion reason: stage 1 - Wrong outcome(s) |
| An Evaluation of an Unstructured and Structured Approach to Increasing Recess Physical Activity | Behrens et al | 2019 | Exclusion reason: stage 1 - Wrong outcome(s) |
| Effectiveness Of A School-Based Multicomponent Intervention On Nutritional Status Among Primary School Children In Bangkok, Thailand | Chawla et al | 2017 | Exclusion reason: stage 1 - Wrong outcome(s) |
| Effectiveness of the Sport Education Fitness Model on Fitness Levels, Knowledge, and Physical Activity | Pritchard et al | 2015 | Exclusion reason: stage 1 - Wrong outcome(s) |
| Effectiveness of a school-based program focusing on diet and health habits taught through physical exercise | Pablos et al | 2018 | Exclusion reason: stage 1 - Wrong outcome(s) |
| School-Based Health Promotion Initiative Increases Children's Physical Activity | Cluss et al | 2016 | Exclusion reason: stage 1 - Wrong outcome(s) |
| Improvements in fundamental movement skill competency mediate the effect of the SCORES intervention on physical activity and cardiorespiratory fitness in children | Cohen et al | 2015 | Exclusion reason: stage 1 - Wrong outcome(s) |
| The Effect of Organized Versus Supervised Recess on Elementary School Children's Participation, Physical Activity, Play, and Social Behavior: A Cluster Randomized Controlled Trial | Coolkens et al | 2018 | Exclusion reason: stage 1 - Wrong outcome(s) |
| Long-term effects of physically active academic lessons on physical fitness and executive functions in primary school children | de Greeff et al | 2016 | Exclusion reason: stage 1 - Wrong outcome(s) |
| Sport Education model effects in the social climate classroom, perceived competence and intent to be physically active: an extended study in primary education | de Ojeda Perez et al | 2016 | Exclusion reason: stage 1 - Wrong outcome(s) |
| Targeting the Body and the Mind: Evaluation of a P.E. Curriculum Intervention for Adolescents | Loukaitou-Sideris et al | 2015 | Exclusion reason: stage 1 - Wrong outcome(s) |
| Twelve-Month Effects of the COPE Healthy Lifestyles TEEN Program on Overweight and Depressive Symptoms in High School Adolescents | Melnyk et al | 2015 | Exclusion reason: stage 1 - Wrong outcome(s) |
| The daily mile: Teachers' perspectives of the barriers and facilitators to the delivery of a school-based physical activity intervention | Malden et al | 2019 | Exclusion reason: stage 1 - Wrong outcome(s) |
| Teachers' and students' perspectives of participating in the 'Active Classrooms' movement integration programme | Martin et al | 2017 | Exclusion reason: stage 1 - Wrong outcome(s) |
| Increasing physical activity levels in primary school physical education: The SHARP Principles Model | Powell et al | 2016 | Exclusion reason: stage 1 - Wrong outcome(s) |
| The Personal and Social Responsibility Model to Enhance Innovation in Physical Education | Prat et al | 2019 | Exclusion reason: stage 1 - Wrong outcome(s) |
| Teacher perceptions on the delivery and implementation of movement integration strategies: The CLASS PAL (Physically Active Learning) Programme | Routen et al | 2018 | Exclusion reason: stage 1 - Wrong outcome(s) |
| Promoting Healthy Lifestyles to Children at School: Using a Multidisciplinary Train-the-Trainer Approach | Sanders et al | 2015 | Exclusion reason: stage 1 - Wrong outcome(s) |
| Implementation of brain breaks in the classroom and effects on attitudes toward physical activity in a macedonian school setting | Popeska et al | 2018 | Exclusion reason: stage 1 - Wrong outcome(s) |
| Impacts of an intervention to reduce sedentary behaviour on measures of obesity in primary school children: A cluster controlled study | Loosemore et al | 2019 | Exclusion reason: stage 1 - Wrong outcome(s) |
| "I want to do it all day!"-Students' experiences of classroom movement integration | McMullen et al | 2019 | Exclusion reason: stage 1 - Wrong outcome(s) |
| "I Just Like the Feeling of It, Outside Being Active": Pupils' Experiences of a School-Based Running Program, a Qualitative Study | Chalkley et al | 2020 | Exclusion reason: stage 1 - Wrong outcome(s) |
| 'FIFA 11 for Health' for Europe. 1: effect on health knowledge and well-being of 10-to 12-year-old Danish school children | Fuller et al | 2017 | Exclusion reason: stage 1 - Wrong outcome(s) |
| Effects of a school based intervention to promote healthy habits in children 8-11 years old, living in the lowland area of Bologna Local Health Unit | Sacchetti et al | 2015 | Exclusion reason: stage 1 - Wrong outcome(s) |
| Effects of a futsal periodized program on physical fitness of female students of 13 and 14 years of age | Fiorante et al | 2018 | Exclusion reason: stage 1 - Wrong outcome(s) |
| Effectiveness of Secondary School Conceptual Physical Education: A 20-Year Longitudinal Study | Kulinna et al | 2018 | Exclusion reason: stage 1 - Wrong outcome(s) |
| Effectiveness of a School Based Intervention for Prevention of Non-communicable Diseases in Middle School Children of Rural North India: A Randomized Controlled Trial | Saraf et al | 2015 | Exclusion reason: stage 1 - Wrong outcome(s) |
| Effectiveness of a Playground Intervention for Antisocial, Prosocial, and Physical Activity Behaviors | Mayfield et al | 2017 | Exclusion reason: stage 1 - Wrong outcome(s) |
| Effectiveness of a 5-year school-based intervention programme to reduce adiposity and improve fitness and lifestyle in Indian children; the SYM-KEM study | Bhave et al | 2016 | Exclusion reason: stage 1 - Wrong outcome(s) |
| Effectiveness evaluation of a health promotion programme in primary schools: a cluster randomised controlled trial | Grillich et al | 2016 | Exclusion reason: stage 1 - Wrong outcome(s) |
| Effective implementation of primary school-based healthy lifestyle programmes: a qualitative study of views of school staff | Day et al | 2019 | Exclusion reason: stage 1 - Wrong outcome(s) |
| Effect of an Elementary School Walking Program on Physical Activity and Classroom Behavior | Lassiter et al | 2019 | Exclusion reason: stage 1 - Wrong outcome(s) |
| Disseminating Evidence-Based Physical Education Practices in Rural Schools: The San Luis Valley Physical Education Academy | Belansky et al | 2016 | Exclusion reason: stage 1 - Wrong outcome(s) |
| Assessing the Wider Implementation of the SHARP Principles: Increasing Physical Activity in Primary Physical Education | Powell et al | 2020 | Exclusion reason: stage 1 - Wrong outcome(s) |
| An innovative school-based intervention to promote healthy lifestyles | Piana et al | 2017 | Exclusion reason: stage 1 - Wrong outcome(s) |
| Adolescents' perspectives on a school-based physical activity intervention: A mixed method study | Jong et al | 2020 | Exclusion reason: stage 1 - Wrong outcome(s) |
| Project FIT: A School, Community and Social Marketing Intervention Improves Healthy Eating Among Low-Income Elementary School Children | Alaimo et al | 2015 | Exclusion reason: stage 1 - Wrong outcome(s) |
| Feasibility of breaking up sitting time in mainstream and special schools with a cognitively challenging motor task | Mazzoli et al | 2019 | Exclusion reason: stage 1 - Wrong outcome(s) |
| Implementation of an intervention program with physical activity and healthy diet for health promotion at school: A possible challenge | Tkac et al | 2017 | Exclusion reason: stage 1 - Wrong outcome(s) |
| The effects of an enhanced quality Physical Education programme on the physical activity levels of Grade 7 learners in Potchefstroom, South Africa | Tian et al | 2017 | Exclusion reason: stage 1 - Wrong outcome(s) |
| Long-term follow-up on biological risk factors, adiposity, and cardiorespiratory fitness development in a physical education intervention: a natural experiment (CHAMPS-study DK) | Tarp et al | 2018 | Exclusion reason: stage 1 - Wrong outcome(s) |
| Process evaluation of a pilot multi-component physical activity intervention - active schools: Skelmersdale | Taylor et al | 2018 | Exclusion reason: stage 1 - Wrong outcome(s) |
| Project SoL-A community-based, multi-component health promotion intervention to improve eating habits and physical activity among Danish families with young children. Part 1: Intervention development and implementation | Toft et al | 2018 | Exclusion reason: stage 1 - Wrong outcome(s) |
| Implementation of KEIGAAF in Primary Schools: A Mutual Adaptation Physical Activity and Nutrition Intervention | Verjans-Janssen et al | 2020 | Exclusion reason: stage 1 - Wrong outcome(s) |
| A retrospective qualitative evaluation of barriers and facilitators to the implementation of a school-based running programme | Chalkley et al | 2018 | Exclusion reason: stage 1 - Wrong outcome(s) |
| Barriers and facilitators to the implementation of a school-based physical activity policy in Canada: application of the theoretical domains framework | Weatherson et al | 2017 | Exclusion reason: stage 1 - Wrong outcome(s) |
| A Mixed Methods Study to Examine the Influence of CLIL on Physical Education Lessons: Analysis of Social Interactions and Physical Activity Levels | Salvador-Garcia et al | 2020 | Exclusion reason: stage 1 - Wrong outcome(s) |
| A Pilot Intervention Using Gamification to Enhance Student Participation in Classroom Activity Breaks | Beemer et al | 2019 | Exclusion reason: stage 1 - Wrong outcome(s) |
| Negotiating the Daily Mile Challenge; looking-like a walking break from the classroom | Ward et al | 2019 | Exclusion reason: stage 1 - Wrong outcome(s) |
| An Exploratory Study of Elementary Classroom Teachers' Physical Activity Promotion From a Social Learning Perspective | Webster et al | 2015 | Exclusion reason: stage 1 - Wrong outcome(s) |
| Building school-based cardiovascular health promotion capacity in youth: a mixed methods study | Woodgate et al | 2015 | Exclusion reason: stage 1 - Wrong outcome(s) |
| Predicting physical exercise changes in Chinese rural adolescents: the application of the health action process approach model | Xu et al | 2019 | Exclusion reason: stage 1 - Wrong outcome(s) |
| Impact evaluation of educational-motivational intervention "Como Jugando" to prevent obesity in school children of Cercado de Lima: results in the first year | Aparco et al | 2017 | Exclusion reason: stage 1 - Wrong outcome(s) |
| Transferring primary generalists' positive classroom pedagogy to the physical education setting: a collaborative PE-CPD process | Morgan et al | 2019 | Exclusion reason: stage 1 - Wrong outcome(s) |
| Qualitative Evaluation Of A School Intervention For The Promotion Of Physical Activity: Learning From The Perspective Of The Target Population | Javier Beltran-Carrillo et al | 2017 | Exclusion reason: stage 1 - Wrong outcome(s) |
| Improvement in game performance and adherence after an aligned TGfU floorball unit in physical education | MoralesBelando et al | 2018 | Exclusion reason: stage 1 - Wrong outcome(s) |
| Project Energize: intervention development and 10 years of progress in preventing childhood obesity | Rush et al | 2016 | Exclusion reason: stage 1 - Wrong outcome(s) |
| The Daily Mile: What factors are associated with its implementation success? | Ryde et al | 2018 | Exclusion reason: stage 1 - Wrong outcome(s) |
| Promoting motor skills in low-income, ethnic children: The Physical Activity in Linguistically Diverse Communities (PALDC) nonrandomized trial | Okely et al | 2017 | Exclusion reason: stage 1 - Wrong outcome(s) |
| The effect of the implementation of a planned peer group session model on obesity prevention among students of an integrated islamic primary school in Makassar | Hadi et al | 2019 | Exclusion reason: stage 1 - Wrong outcome(s) |
| A Self-determination theory based intervention to promote healthy eating and physical activity in school-aged children. | Girelli et al | 2016 | Exclusion reason: stage 1 - Wrong outcome(s) |
| A School-Based Motivational Intervention to Promote Physical Activity from a Self-Determination Theory Perspective | González-Cutre et al | 2018 | Exclusion reason: stage 1 - Wrong outcome(s) |
| Enhancing Physical Education with Exergames and Wearable Technology | Lindberg et al | 2016 | Exclusion reason: stage 1 - Wrong outcome(s) |
| Effectiveness of a school-based program to prevent obesity | Perez Solis et al | 2015 | Exclusion reason: stage 1 - Wrong outcome(s) |
| Study and promotion of healthy eating habits and physical activity among Spanish adolescents: TAS program (you and Alicia for health) | Pareja Sierra et al | 2018 | Exclusion reason: stage 1 - Wrong outcome(s) |
| A quasi-experimental examination of how school-based physical activity changes impact secondary school student moderate- to vigorous- intensity physical activity over time in the COMPASS study | Hunter et al | 2016 | Exclusion reason: stage 1 - Wrong outcome(s) |
| When mathematics meets physical activity in the school-aged child: The effect of an integrated motor and cognitive approach to learning geometry | Hraste et al | 2018 | Exclusion reason: stage 1 - Wrong outcome(s) |
| Effects of the COPE Cognitive Behavioral Skills Building TEEN Program on the Healthy Lifestyle Behaviors and Mental Health of Appalachian Early Adolescents | Hoying et al | 2016 | Exclusion reason: stage 1 - Wrong outcome(s) |
| Effects of a sports-oriented primary school on students’ physical literacy and cognitive performance | Demetriou et al | 2018 | Exclusion reason: stage 1 - Wrong outcome(s) |
| Sport education model versus traditional model: effects on motivation and sportsmanship | Mendez-Gimenez et al | 2015 | Exclusion reason: stage 1 - Wrong outcome(s) |
| Evaluation of an Intervention to Increase Physical Activity in Low-Income, Urban Middle Schools | Gill et al | 2019 | Exclusion reason: stage 1 - Wrong outcome(s) |
| The effects of introducing Tabata interval training and stability exercises to school children as a school-based intervention program | Ekstrom et al | 2019 | Exclusion reason: stage 1 - Wrong outcome(s) |
| A Physical Activity Intervention and Changes in Body Mass Index at a Middle School With a Large American Indian Population, Oklahoma, 2004-2009 | Eichner et al | 2016 | Exclusion reason: stage 1 - Wrong outcome(s) |
| The Effect of Music- and Video-Distraction on High School Physical Education Student Exercise Intensity | Higginson et al | 2019 | Exclusion reason: stage 1 - Wrong outcome(s) |
| The effect of nutritional and physical activity interventions on nutritional status and obesity in primary school children: A cluster randomized controlled study | Akdemir et al | 2017 | Exclusion reason: stage 1 - Wrong outcome(s) |
| Reducing Obesity in Students Everywhere (ROSE): A Brief, Interactive, School-Based Approach to Promoting Health | Alert et al | 2015 | Exclusion reason: stage 1 - Wrong outcome(s) |
| Barriers and facilitators to adoption, implementation and sustainment of obesity prevention interventions in schoolchildren- a DEDIPAC case study | Hayes et al | 2019 | Exclusion reason: stage 1 - Wrong outcome(s) |
| Physical education of students, considering their physical fitness level | Andres et al | 2017 | Exclusion reason: stage 1 - Wrong outcome(s) |
| Adiposity and response to an obesity prevention intervention in Pakistani and Bangladeshi primary school boys and girls: a secondary analysis using the BEACHeS feasibility study | Cezard et al | 2016 | Exclusion reason: stage 1 - Wrong outcome(s) |
| Effect of a 20-week physical activity intervention on selective attention and academic performance in children living in disadvantaged neighborhoods: A cluster randomized control trial | Gall et al | 2018 | Exclusion reason: stage 1 - Wrong outcome(s) |
| One-Year Follow-up of the CAPO Kids Trial: Are Physical Benefits Maintained? | Nogueira et al | 2017 | Exclusion reason: stage 1 - Wrong outcome(s) |
| Effects of a brief physical activity program on young students' physical fitness | Hayes et al | 2015 | Exclusion reason: stage 1 - Wrong outcome(s) |
| Cluster randomised trial of a school-community child health promotion and obesity prevention intervention: findings from the evaluation of fun 'n healthy in Moreland! | Waters et al | 2017 | Exclusion reason: stage 1 - Wrong outcome(s) |
| Virtual field trips as physically active lessons for children: a pilot study | Norris et al | 2015 | Exclusion reason: stage 1 - Wrong outcome(s) |
| Examining the impact of a province-wide physical education policy on secondary students' physical activity as a natural experiment | Hobin et al | 2017 | Exclusion reason: stage 1 - Wrong outcome(s) |
| The effect of a two-year school-based daily physical activity intervention on a clustered CVD risk factor score-The Sogndal school-intervention study | Resaland et al | 2018 | Exclusion reason: stage 1 - Wrong outcome(s) |
| Effects of a School‐Based Pedometer Intervention in Adolescents: 1‐Year Follow‐Up of a Cluster‐Randomized Controlled Trial | Isensee et al | 2018 | Exclusion reason: stage 1 - Wrong outcome(s) |
| Effects of a Randomised Controlled School-Based Health Promotion Intervention on Obesity Related Behavioural Outcomes of Children with Migration Background | Kobel et al | 2017 | Exclusion reason: stage 1 - Wrong outcome(s) |
| Food, Health, & Choices: Curriculum and Wellness Interventions to Decrease Childhood Obesity in Fifth-Graders | Koch et al | 2019 | Exclusion reason: stage 1 - Wrong outcome(s) |
| A professional development system to optimize the implementation of a daily physical activity program in a school setting | Beaudoin et al | 2018 | Exclusion reason: stage 1 - Wrong outcome(s) |
| Lessons learned from the AFLY5 RCT process evaluation: implications for the design of physical activity and nutrition interventions in schools | Jago et al | 2015 | Exclusion reason: stage 1 - Wrong outcome(s) |
| Long term effect of a school based intervention to prevent chronic diseases in Tunisia, 2009-2015 | Ghammam et al | 2017 | Exclusion reason: stage 1 - Wrong outcome(s) |
| Does health promotion in primary schools work? A randomized waiting list control group study for the Klasse2000 program | Kolip et al | 2017 | Exclusion reason: stage 1 - Wrong outcome(s) |
| Active breaks to improve class attention: Educational Interventions | Suarez-Manzano et al | 2018 | Exclusion reason: stage 1 - Wrong outcome(s) |
| Exploring the use of a gamified intervention for encouraging physical activity in adolescents: A qualitative longitudinal study in Northern Ireland | Corepal et al | 2018 | Exclusion reason: stage 1 - Wrong outcome(s) |
| Effect of an obesity prevention program focused on motivating environments in childhood: a school-based prospective study | Yang et al | 2017 | Exclusion reason: stage 1 - Wrong outcome(s) |
| Implementing School-Based Policies to Prevent Obesity: Cluster Randomized Trial | Ickovics et al | 2019 | Exclusion reason: stage 1 - Wrong outcome(s) |
| Effects of an Integrated Health Care Program for Children | Kim et al | 2017 | Exclusion reason: stage 1 - Wrong outcome(s) |
| The Viennese Prevention Study (EDDY): initial results | Poeppelmeyer et al | 2016 | Exclusion reason: stage 1 - Wrong outcome(s) |
| Evaluation of Let's Move! active schools activation grants | Miller et al | 2018 | Exclusion reason: stage 1 - Wrong outcome(s) |
| Evaluation of a classroom movement integration training delivered in a low socioeconomic school district | Stewart et al | 2019 | Exclusion reason: stage 1 - Wrong outcome(s) |
| Using Family-Focused Garden, Nutrition, and Physical Activity Programs To Reduce Childhood Obesity: The Texas! Go! Eat! Grow! Pilot Study | Spears-Lanoix et al | 2015 | Exclusion reason: stage 1 - Wrong outcome(s) |
| System-Activity Approach Implemented At Physical Education Lessons | Sinyavsky et al | 2015 | Exclusion reason: stage 1 - Wrong outcome(s) |
| "“FIFA 11 for Health” for Europe in the Faroe Islands: Effects on health markers and physical fitness in 10‐to 12‐year‐old schoolchildren." | Skoradal et al | 2018 | Exclusion reason: stage 1 - Wrong outcome(s) |
| Effectiveness of a creative physical education intervention on elementary school students' leisure-time physical activity motivation and overall physical activity in Finland | Kokkonen et al | 2019 | Exclusion reason: stage 1 - Wrong outcome(s) |
| Professional Development for Increased Classroom-based Physical Activity: Elements and Strategies to Reduce Barriers and Facilitate Implementation | O'Hara Tompkins et al | 2019 | Exclusion reason: stage 1 - Wrong outcome(s) |
| Promoting physical activity with a school-based dance mat exergaming intervention: qualitative findings from a natural experiment | Burges Watson et al | 2016 | Exclusion reason: stage 1 - Wrong outcome(s) |
| The daily mile: 15 minutes running improves the physical fitness of italian primary school children | Brustio et al | 2019 | Exclusion reason: stage 1 - Wrong outcome(s) |
| Association of a behaviorally based high school health education curriculum with increased exercise | Annesi et al | 2015 | Exclusion reason: stage 1 - Wrong outcome(s) |
| The effects of autonomy support in physical education classes | Antonio Moreno-Murcia et al | 2016 | Exclusion reason: stage 1 - Wrong outcome(s) |
| Effect of a multidimensional physical activity intervention on body mass index, skinfolds and fitness in south african children: Results from a cluster-randomised controlled trial | Muller et al | 2019 | Exclusion reason: stage 1 - Wrong outcome(s) |
| Effectiveness Of Yoga-Aerobic Means' Application In Physical Education Of Primary School Pupils | Mykhno et al | 2016 | Exclusion reason: stage 1 - Wrong outcome(s) |
| Case study of a health optimizing physical education-based comprehensive school physical activity program | Egan et al | 2019 | Exclusion reason: stage 1 - Wrong outcome(s) |
| Actions of the School Health Program and school meals in the prevention of childhood overweight: experience in the municipality of Itapevi, Sao Paulo State, Brazil, 2014 | Alves Batista et al | 2017 | Exclusion reason: stage 1 - Wrong outcome(s) |
| A prevention program for multiple health-compromising behaviors in adolescence: Baseline results from a cluster randomized controlled trial | Allara et al | 2015 | Exclusion reason: stage 1 - Wrong outcome(s) |
| Effects of a prevention program on multiple health-compromising behaviours in adolescence: A cluster randomized controlled trial | Allara et al | 2019 | Exclusion reason: stage 1 - Wrong outcome(s) |
| Teachers' Training and Involvement in School Health Programme in Oyo State, Southwest Nigeria | Adebayo et al | 2018 | Exclusion reason: stage 1 - Wrong outcome(s) |
| Implementation practices in school health promotion: findings from an Austrian multiple-case study | Adamowitsch et al | 2017 | Exclusion reason: stage 1 - Wrong outcome(s) |
| Improving students' predisposition towards physical education by optimizing their motivational processes in an acrosport unit | Abós et al | 2017 | Exclusion reason: stage 1 - Wrong outcome(s) |
| Executive Function, Behavioral Self-Regulation, and School Related Well-Being Did Not Mediate the Effect of School-Based Physical Activity on Academic Performance in Numeracy in 10-Year-Old Children. The Active Smarter Kids (ASK) Study | Aadland et al | 2018 | Exclusion reason: stage 1 - Wrong outcome(s) |
| Physically Active Math and Language Lessons Improve Academic Achievement: A Cluster Randomized Controlled Trial | Mullender-Wijnsma et al | 2016 | Exclusion reason: stage 1 - Wrong outcome(s) |
| Implementation of triple the time spent on physical education in pre-school to 6th grade: A qualitative study from the programme managers' perspective | Nielsen et al | 2018 | Exclusion reason: stage 1 - Wrong outcome(s) |
| Implementation of a successful long-term school based physical education intervention: Exploring provider and programme characteristics | Nielsen et al | 2019 | Exclusion reason: stage 1 - Wrong outcome(s) |
| **Stage 2** (n=98) |  |  |  |
| Two-year process evaluation of a pilot program to increase elementary children's physical activity during school | Webster et al | 2018 | Exclusion reason: stage 2 - feasibility/pilot |
| Partnerships for active elementary schools: Physical education outcomes after 4 months of a 2-year pilot study | Weaver et al | 2017 | Exclusion reason: stage 2 - feasibility/pilot |
| Partnerships for Active Children in Elementary Schools: Outcomes of a 2-Year Pilot Study to Increase Physical Activity During the School Day | Weaver et al | 2018 | Exclusion reason: stage 2 - feasibility/pilot |
| An Intervention to Increase Students' Physical Activity: A 2-Year Pilot Study | Weaver et al | 2018 | Exclusion reason: stage 2 - feasibility/pilot |
| Initial Outcomes of a Participatory-Based, Competency-Building Approach to Increasing Physical Education Teachers' Physical Activity Promotion and Students' Physical Activity: A Pilot Study | Weaver et al | 2018 | Exclusion reason: stage 2 - feasibility/pilot |
| Process evaluation of a classroom active break (ACTI-BREAK) program for improving academic-related and physical activity outcomes for students in years 3 and 4 | Watson et al | 2019 | Exclusion reason: stage 2 - feasibility/pilot |
| A pilot primary school active break program (ACTI-BREAK): Effects on academic and physical activity outcomes for students in Years 3 and 4 | Watson et al | 2019 | Exclusion reason: stage 2 - feasibility/pilot |
| The UP4FUN Intervention Effect on Breaking Up Sedentary Time in 10- to 12-Year-Old Belgian Children: The ENERGY Project | Verloigne et al | 2015 | Exclusion reason: stage 2 - feasibility/pilot |
| Evaluation of a pilot school-based physical activity clustered randomised controlled trial-active schools: Skelmersdale | Taylor et al | 2018 | Exclusion reason: stage 2 - feasibility/pilot |
| Acceptability and Feasibility of Single-Component Primary School Physical Activity Interventions to Inform the AS:Sk Project | Taylor et al | 2018 | Exclusion reason: stage 2 - feasibility/pilot |
| Effects of the Quest to Lava Mountain Computer Game on Dietary and Physical Activity Behaviors of Elementary School Children: A Pilot Group-Randomized Controlled Trial | Sharma et al | 2015 | Exclusion reason: stage 2 - feasibility/pilot |
| The feasibility and acceptability of a primary school-based programme targeting diet and physical activity: The PhunkyFoods Programme | Sahota et al | 2019 | Exclusion reason: stage 2 - feasibility/pilot |
| Healthy for life pilot study: A multicomponent school and home based physical activity intervention for disadvantaged children | Pearce et al | 2019 | Exclusion reason: stage 2 - feasibility/pilot |
| Evaluation of an intervention to reduce adolescent sitting time during the school day: The 'stand Up for Health' randomised controlled trial | Parrish et al | 2018 | Exclusion reason: stage 2 - feasibility/pilot |
| Feasibility and Preliminary Efficacy of a Teacher-Facilitated High-Intensity Interval Training Intervention for Older Adolescents | Leahy et al | 2019 | Exclusion reason: stage 2 - feasibility/pilot |
| Randomised controlled feasibility study of a school-based multi-level intervention to increase physical activity and decrease sedentary behaviour among vocational school students | Hankonen et al | 2017 | Exclusion reason: stage 2 - feasibility/pilot |
| Introducing physically active lessons in UK secondary schools: Feasibility study and pilot cluster-randomised controlled trial | Gammon et al | 2019 | Exclusion reason: stage 2 - feasibility/pilot |
| The Daily Mile makes primary school children more active, less sedentary and improves their fitness and body composition: A quasi-experimental pilot study | Chesham et al | 2018 | Exclusion reason: stage 2 - feasibility/pilot |
| Feasibility study and pilot cluster-randomised controlled trial of the GoActive intervention aiming to promote physical activity among adolescents: outcomes and lessons learnt | Corder et al | 2016 | Exclusion reason: stage 2 - feasibility/pilot |
| Stand Out in Class: restructuring the classroom environment to reduce sitting time - findings from a pilot cluster randomised controlled trial | Clemes et al | 2020 | Exclusion reason: stage 2 - feasibility/pilot |
| A Pilot School Sports Program in a Remote Canadian First Nation: Evaluation of Process and Outcomes | Gates et al | 2016 | Exclusion reason: stage 2 - feasibility/pilot |
| Implementing a Nutrition and Physical Activity Curriculum in Head Start Through an Academic-Community Partnership | Zahnd et al | 2017 | Exclusion reason: stage 2 - not an RCT |
| Teaching Games for Understanding Intervention to Promote Physical Activity among Secondary School Students | Wang et al | 2018 | Exclusion reason: stage 2 - not an RCT |
| Process evaluation of the IDEFICS school intervention: Putting the evaluation of the effect on children's objectively measured physical activity and sedentary time in context | Verloigne et al | 2015 | Exclusion reason: stage 2 - not an RCT |
| Effectiveness of the IDEFICS intervention on objectively measured physical activity and sedentary time in European children | Verbestel et al | 2015 | Exclusion reason: stage 2 - not an RCT |
| Effect of Integrated Physical Activities with Mathematics on Objectively Assessed Physical Activity | Vazou et al | 2018 | Exclusion reason: stage 2 - not an RCT |
| Implemented or not implemented? Process evaluation of the school-based obesity prevention program DOiT and associations with program effectiveness | van Nassau et al | 2016 | Exclusion reason: stage 2 - not an RCT |
| "... because there's nobody who can just sit that long." | Stylianou et al | 2016 | Exclusion reason: stage 2 - not an RCT |
| Teacher Fidelity to a Physical Education Curricular Model and Physical Activity Outcomes | Stylianou et al | 2016 | Exclusion reason: stage 2 - not an RCT |
| Purposeful Movement: The Integration of Physical Activity into a Mathematics Unit | Snyder et al | 2017 | Exclusion reason: stage 2 - not an RCT |
| Impact of a classroom standing desk intervention on daily objectively measured sedentary behavior and physical activity in youth | Silva et al | 2018 | Exclusion reason: stage 2 - not an RCT |
| Can High Schools Be an Effective Setting to Promote Healthy Lifestyles? Effects of a Multiple Behavior Change Intervention in Adolescents | Sevil et al | 2019 | Exclusion reason: stage 2 - not an RCT |
| Changes in physical activity, physical fitness and well-being following a school-based health promotion program in a Norwegian region with a poor public health profile: A non-randomized controlled study in early adolescents | Schmidt et al | 2020 | Exclusion reason: stage 2 - not an RCT |
| An Evaluation of a Video-based Physical Activity Intervention in the Classrooms of Elementary Schoolchildren | Schmidt et al | 2017 | Exclusion reason: stage 2 - not an RCT |
| Students' physical activity and teachers' motivational styles in physical education | Rupprich et al | 2016 | Exclusion reason: stage 2 - not an RCT |
| Skipping Hearts Goes to School: Short-Term Effects. / Skipping Hearts macht Schule: Kurzzeiteffekte des Projekts | Postler et al | 2017 | Exclusion reason: stage 2 - not an RCT |
| Using the RE-AIM framework to evaluate a school-based municipal programme tripling time spent on PE | Nielsen et al | 2018 | Exclusion reason: stage 2 - not an RCT |
| A mixed-methods exploration of implementation of a comprehensive school healthy eating model one year after scale-up | Naylor et al | 2016 | Exclusion reason: stage 2 - not an RCT |
| Effects of the 3-year Sigue la Huella intervention on sedentary time in secondary school students | Murillo Pardo et al | 2015 | Exclusion reason: stage 2 - not an RCT |
| Improving academic performance of school-age children by physical activity in the classroom: 1-year program evaluation | Mullender-Wijnsma et al | 2015 | Exclusion reason: stage 2 - not an RCT |
| Preliminary findings of Active Classrooms: An intervention to increase physical activity levels of primary school children during class time | Martin et al | 2015 | Exclusion reason: stage 2 - not an RCT |
| Teacher Physical Education Practices and Student Outcomes in a Sample of Middle Schools Participating in the Presidential Youth Fitness Program | Lucas et al | 2019 | Exclusion reason: stage 2 - not an RCT |
| The effects of teacher fidelity of implementation of pathways to health on student outcomes | Little et al | 2015 | Exclusion reason: stage 2 - not an RCT |
| Process Evaluation of an Early Care and Education Intervention: The California Childhood Obesity Research Demonstration Study (CA-CORD) | Lin et al | 2020 | Exclusion reason: stage 2 - not an RCT |
| Results of a 3-year, nutrition and physical activity intervention for children in rural, low-socioeconomic status elementary schools | King et al | 2015 | Exclusion reason: stage 2 - not an RCT |
| Classroom standing desks and time-series variation in sedentary behavior and physical activity among primary school children | Kidokoro et al | 2019 | Exclusion reason: stage 2 - not an RCT |
| Increasing Physical Activity in Schools: Strategies for School Health Practitioners | Kelly et al | 2019 | Exclusion reason: stage 2 - not an RCT |
| The Role of Classroom Teacher Social Capital in a Comprehensive School Physical Activity Program | Jordan et al | 2018 | Exclusion reason: stage 2 - not an RCT |
| The effect of a curriculum-based physical activity intervention on accelerometer-assessed physical activity in schoolchildren: A non-randomised mixed methods controlled before-and-after study | Innerd et al | 2019 | Exclusion reason: stage 2 - not an RCT |
| Evaluation of a student participatory, low-intensity program to improve school wellness environment and students' eating and activity behaviors | Hoelscher et al | 2016 | Exclusion reason: stage 2 - not an RCT |
| Tactical Games Model and Its Effects on Student Physical Activity and Gameplay Performance in Secondary Physical Education | Hodges et al | 2018 | Exclusion reason: stage 2 - not an RCT |
| Gender and School-Level Differences in Students' Moderate and Vigorous Physical Activity Levels When Taught Basketball Through the Tactical Games Model | Harvey et al | 2016 | Exclusion reason: stage 2 - not an RCT |
| Evaluation of a School-Community Linked Physical Activity Intervention Targeting 7- to 12-Year-Olds: A Sociocultural Perspective | Griffiths et al | 2019 | Exclusion reason: stage 2 - not an RCT |
| Effect Of Spark On Physical Activity, Cardiorespiratory Endurance, And Motivation In Middle-school Students | Fu et al | 2015 | Exclusion reason: stage 2 - not an RCT |
| Young Children's School Day Sedentary Behavior and Physical Activity in Interactive versus Non-Interactive Active Video Games | Fu et al | 2019 | Exclusion reason: stage 2 - not an RCT |
| Academic-Based and Aerobic-Only Movement Breaks: Are There Differential Effects on Physical Activity and Achievement? | Fedewa et al | 2018 | Exclusion reason: stage 2 - not an RCT |
| Partnerships for Active Children in Elementary Schools (PACES): First year process evaluation | Egan et al | 2018 | Exclusion reason: stage 2 - not an RCT |
| The effect of the daily mile on primary school children's aerobic fitness levels after 12 weeks: A controlled trial | de Jonge et al | 2020 | Exclusion reason: stage 2 - not an RCT |
| The effect of an intervention on physical activity of moderate-and-vigorous intensity, and sedentary behavior during adolescents' time at school | Costa et al | 2019 | Exclusion reason: stage 2 - not an RCT |
| Involving the headteacher in the development of school-based health interventions: A mixed-methods outcome and process evaluation using the RE-AIM framework | Christian et al | 2020 | Exclusion reason: stage 2 - not an RCT |
| Impact of a Georgia elementary school-based intervention on physical activity opportunities: A quasi-experimental study | Cheung et al | 2019 | Exclusion reason: stage 2 - not an RCT |
| Implementing classroom physical activity breaks: Associations with student physical activity and classroom behavior | Carlson et al | 2015 | Exclusion reason: stage 2 - not an RCT |
| Contextual factors related to implementation of classroom physical activity breaks | Carlson et al | 2017 | Exclusion reason: stage 2 - not an RCT |
| School Day Classroom-Based Physical Activity and Sedentary Behavior | Calvert et al | 2019 | Exclusion reason: stage 2 - not an RCT |
| Classroom active breaks: a feasibility study in Southern Italy | Calella et al | 2019 | Exclusion reason: stage 2 - not an RCT |
| Effect of a Comprehensive School Physical Activity Program on School Day Step Counts in Children | Burns et al | 2015 | Exclusion reason: stage 2 - not an RCT |
| Can Fundamental Movement Skill Mastery Be Increased via a Six Week Physical Activity Intervention to Have Positive Effects on Physical Activity and Physical Self-Perception? | Bryant et al | 2016 | Exclusion reason: stage 2 - not an RCT |
| The Physical Activity Leader and Comprehensive School Physical Activity Program Effectiveness | Brusseau et al | 2018 | Exclusion reason: stage 2 - not an RCT |
| Physical activity patterns associated with a pedagogical intervention in six to eight year old children in an urban school | Briceno et al | 2019 | Exclusion reason: stage 2 - not an RCT |
| Effects of Music on Physical Activity Rates of Junior High School Physical Education Students | Brewer et al | 2016 | Exclusion reason: stage 2 - not an RCT |
| A program evaluation of an in-school daily physical activity initiative for children and youth | Bremer et al | 2018 | Exclusion reason: stage 2 - not an RCT |
| Impact of an Elementary School-Based Intervention on Physical Activity Time and Aerobic Capacity, Georgia, 2013-2014 | Braun et al | 2017 | Exclusion reason: stage 2 - not an RCT |
| Evaluation of the dissemination of SNaX, a middle school-based obesity prevention intervention, within a large US school district | Bogart et al | 2018 | Exclusion reason: stage 2 - not an RCT |
| Using School Staff Members to Implement a Childhood Obesity Prevention Intervention in Low-Income School Districts: the Massachusetts Childhood Obesity Research Demonstration (MA-CORD Project), 2012-2014 | Blaine et al | 2017 | Exclusion reason: stage 2 - not an RCT |
| Bridging Public Health and Education: Results of a School-Based Physical Activity Program to Increase Student Fitness | Barrett-Williams et al | 2017 | Exclusion reason: stage 2 - not an RCT |
| The Longitudinal Impact of NFL PLAY 60 Programming on Youth Aerobic Capacity and BMI | Bai et al | 2017 | Exclusion reason: stage 2 - not an RCT |
| Effects of "Fair Play Game" Strategy on Moderate to Vigorous Physical Activity in Physical Education | Azevedo et al | 2016 | Exclusion reason: stage 2 - not an RCT |
| The impact of height-adjustable desks and prompts to break-up classroom sitting on adolescents' energy expenditure, adiposity markers and perceived musculoskeletal discomfort | Ayala et al | 2018 | Exclusion reason: stage 2 - not an RCT |
| Impact of an 8-month trial using height-adjustable desks on children's classroom sitting patterns and markers of cardio-metabolic and musculoskeletal health | Ayala et al | 2016 | Exclusion reason: stage 2 - not an RCT |
| Children's segment specific light physical activity across two years of schoolbased program | Arto et al | 2015 | Exclusion reason: stage 2 - not an RCT |
| Development of an educational intervention to promote healthy eating and physical activity in Mexican school-age children | Amaya-Castellanos et al | 2015 | Exclusion reason: stage 2 - not an RCT |
| School and classroom effects on Daily Physical Activity (DPA) policy implementation fidelity in Ontario classrooms: a multi-level analysis | Allison et al | 2018 | Exclusion reason: stage 2 - not an RCT |
| The Patterns of Moderate to Vigorous Physical Activity and Physical Education Enjoyment Through a 2‐Year School‐Based Program. | Gråstén et al | 2019 | Exclusion reason: stage 2 - not an RCT |
| Children's segment specific moderate to vigorous physical activity through a school-initiated physical activity program | Gråstén et al | 2015 | Exclusion reason: stage 2 - not an RCT |
| Effectiveness of school-initiated physical activity program on secondary school students' physical activity participation | Grasten et al | 2015 | Exclusion reason: stage 2 - not an RCT |
| Children’s Physical Activity and On-Task Behavior Following Active Academic Lessons | Goh et al | 2017 | Exclusion reason: stage 2 - not an RCT |
| Impact of exergaming on young children's school day energy expenditure and moderate-to-vigorous physical activity levels | Gao et al | 2017 | Exclusion reason: stage 2 - not an RCT |
| Effects of active video games on children's psychosocial beliefs and school day energy expenditure | Gao et al | 2019 | Exclusion reason: stage 2 - not an RCT |
| Training teachers to implement physical activity: Applying social cognitive theory | Hivner et al | 2019 | Exclusion reason: stage 2 - not an RCT |
| The impact of 10-minute activity breaks outside the classroom on male students' on-task behaviour and sustained attention: A randomised crossover design | Wilson et al | 2016 | Exclusion reason: stage 2 - small scale study |
| Physical activity levels and motivational responses of boys and girls: A comparison of direct instruction and tactical games models of games teaching in physical education | Smith et al | 2015 | Exclusion reason: stage 2 - small scale study |
| Physical activity levels, game performance and friendship goals using two different pedagogical models: Sport Education and Direct Instruction | Rocamora et al | 2019 | Exclusion reason: stage 2 - small scale study |
| Impact of a Sustained TPSR Program on Students' Responsibility, Motivation, Sportsmanship, and Intention To Be Physically Active | Merino-Barrero et al | 2020 | Exclusion reason: stage 2 - small scale study |
| The influence of content knowledge on teaching and learning in Traditional and Sport Education contexts: an exploratory study | Iserbyt et al | 2016 | Exclusion reason: stage 2 - small scale study |
| Investigation of Pupils' Levels of MVPA and VPA During Physical Education Units Focused on Direct Instruction and Tactical Games Models | Harvey et al | 2015 | Exclusion reason: stage 2 - small scale study |
| Interdependent Group Contingency to Promote Physical Activity in Children | Foote et al | 2017 | Exclusion reason: stage 2 - small scale study |
| Effects of an Interdisciplinary Approach Integrating Mathematics and Physical Education on Mathematical Learning and Physical Activity Levels | Cecchini et al | 2020 | Exclusion reason: stage 2 - small scale study |
| Effects of Classroom-Based Energizers on Primary Grade Students' Physical Activity Levels | Bailey et al | 2015 | Exclusion reason: stage 2 - small scale study |
