# Supplementary material for: Features of effective staff training programmes within school-based interventions targeting student activity behaviour: a systematic review and meta-analysis
Source: Int J Behav Nutr Phys Act. 2022 Sep 24;19:125. doi: 10.1186/s12966-022-01361-6 (PMC9509574; doi:10.1186/s12966-022-01361-6)
Supplement: Supplementary file 6 — Additional file 6. Table of descriptive characteristics of studies (n = 51) included in systematic review of school-based activity behaviour interventions. [file 12966_2022_1361_MOESM6_ESM.docx]

Additional File 6. Table of descriptive characteristics of studies (n=51) included in systematic review of school-based activity behaviour interventions

| **Study**  Trial name ^References^  Lead author of publication  Year of publication  Country | **Design**  Study design;  level of randomisation (where applicable) | **Intervention (student)**  Intervention study duration  School setting  Number of schools at baseline  Main aim of intervention  PA/SB targeted in any non-school settings? (e.g. via homework/after-school club, parent engagement?) (yes/no) | **Participants (student)**  Number of students at baseline  Students’ mean age: years (± SD) at baseline | **Teacher role**  Description of teacher role  Training duration  Any theory/rationale used to inform training? (yes/no)  BCTs in intervention group (see Table 3 for further details)  Quantitative fidelity data reported? (yes/no) | **PA measurement**  Eligible data reported  Device used  Cut points used  PA intensity, units and outcome period selected for review  PA follow-up time closest to intervention end-point | **SB measurement**  Eligible data reported  Device used  Cut points used  SB outcome and period selected for review  SB follow-up time closest to intervention end-point |
| --- | --- | --- | --- | --- | --- | --- |
| ASK^1^  Aadland et al  2019  Norway | Cluster RCT  School level | 7-month intervention  Primary school  setting  57 schools at baseline  Aim: to create a number of varied PA activities that could be carried out in small groups and which encouraged an inclusive and joyful learning environment, generating an additional 165 mins/week of PA  PA/SB targeted outside of school time - Yes | 1,129 students at baseline  Students’ mean age: 10.2yrs (±0.3) | Teacher role: to implement physically active educational lessons (3 × 30 mins/week) in the subjects Norwegian, mathematics, and English, and PA breaks during classroom lessons (5 mins/school day)  60 hours of teacher training  Any rationale/theory used to inform training? Yes - the competence motivation theory,  the achievement goal theory, and the self-determination  theory  3 BCTs identified  ✓ Teacher fidelity data reported | ✓ PA data reported  Device used: ActiGraph  Cut points: Evenson 2008  MVPA mins during school hours  7-month FU after baseline | ✓ SB data reported  Device used: ActiGraph  Cut points: Evenson 2008  Sedentary mins during  school hours  7-month FU after baseline |
| The West Midlands ActiVe lifestyle and healthy Eating in School children (WAVES)^2^  Adab et al  2018  United Kingdom | Cluster RCT  School level | 12-month intervention  Primary school setting  54 schools at baseline  Aim: To prevent excess weight in primary school children  PA/SB targeted outside of teacher period - Yes | 1,397 students at baseline  Students’ mean age: 6.3yrs (±0.3) | Teacher role: to introduce daily opportunity for additional 30 mins of moderate to vigorous physical activity in bouts of >5 mins through classroom or playground routines, supervise class participation in a healthy lifestyle programme, and hand out two signposting sheets promoting physical activity and directing children/families to activity opportunities available in their local area  7 hours of teacher training  Any rationale/theory used to inform training?  No  3 BCTs identified  ✓ Teacher fidelity data reported | ✓ PA data reported  Device used: Actiheart  Cut points: WHO 1995  MVPA mins per day (any day, including weekends)  15-month FU after baseline | ✓ SB data reported  Device used: Actiheart  Cut points: WHO 1995  Sedentary mins per day (any day, including weekends)  15-month FU after baseline |
| KIDS OUT!^3^  Aittasalo et al  2019  Finland | Cluster RCT  School level | 3-week intervention  Secondary school setting  14 schools at baseline  Aim: to promote physical activity and reduce screen time  PA/SB targeted outside of school time - Yes | 1,476 students at baseline  Students’ mean age: 13.9yrs (±0.5) | Teacher role: to integrate specific content on PA and SB into routinely scheduled three Health Education lessons with the help of educational material  1 hour of teacher training  Any rationale/theory used to inform training?  No  1 BCT identified  ✓ Teacher fidelity data reported | X eligible PA data not reported | X eligible SB data not reported |
| Active for Life Year 5 (AFLY5)^4^  Anderson et al  2016  United Kingdom | Cluster RCT  School level | 12-month intervention  Primary school setting  60 schools at baseline  Aim: to increase physical activity, reduce sedentary behaviours and improve diet  PA/SB targeted outside of school time - Yes | 2,123 students at baseline  Students’ mean age: 9.5yrs (±0.3) | Teacher role: teachers were instructed to deliver 16 lessons, 10 of which had associated homework.  8.5 hours of teacher training  Any rationale/theory used to inform training?  - Not reported  3 BCTs identified  ✓ Teacher fidelity data reported | ✓ PA data reported  Device used: ActiGraph  Cut points: Evenson 2008  MVPA mins per day (any day, including weekends)  12-month FU after baseline | ✓ SB data reported  Device used: ActiGraph  Cut points: Evenson 2008  Sedentary mins per day (any day, including weekends)  12-month FU after baseline |
| Youth-Physical Activity Towards Health (Y-PATH)^5^  Belton et al  2019  Ireland | Cluster RCT  School level | 24-month intervention  Secondary school setting  20 schools at baseline  Aim: a whole-school multi-component intervention programme, aimed at reducing the age-related decline of MVPA in adolescents  PA/SB targeted outside of school time - Yes | 490 students at baseline  Students’ mean age: 12.8yrs (±0.4) | Teacher role: teachers asked to change focus, direction and philosophy of delivery within exiting PE curriculum  4 hours of teacher training  Any rationale/theory used to inform training?  Yes - the Youth Physical Activity Promotion model, the Self-determination theory, and the socio-ecological model  3 BCTs identified  X No eligible fidelity data reported | ✓ PA data reported  Device used: ActiGraph  Cut points: Evenson 2008  MVPA mins per day (any day, including weekends)  24-month FU after baseline | X eligible SB data not reported |
| Sydney Playground Project^6^  Bundy et al  2017  Australia | Cluster RCT  School level | 13-week intervention  Primary school setting  12 schools at baseline  Aim: to promote play through an intervention and using this process, enhance physical activity, social skills, and perceived competence/social acceptance of 5- to 7-year-old children in a school playground environment  PA/SB targeted outside of school time - Yes | 221 students at baseline  Students’ mean age: 6.0yrs (±0.6) | Teacher role: to tolerate more risk during free play  2 hours of teacher training  Any rationale/theory used to inform training?  Yes - the International Classification of Functioning, Disability and Health was used as the broad basis for the study with the idea that changing the environment (social and physical) would change participating children’s experiences. The social cognitive theory was used as a basis for the adult risk reframing intervention.  5 BCTs identified  X No eligible fidelity data reported | ✓ PA data reported  Device used: ActiGraph  Cut points: Evenson 2008  MVPA total counts during teacher period  13-week FU after baseline | ✓ SB data reported  Device used: ActiGraph  Cut points: Evenson 2008  Sedentary mins during teacher period  13-week FU after baseline |
| An Assessment-based intervention on Fundamental Movement Skills (A+FMS)^7^  Chan et al  2019  Hong Kong | Cluster RCT  Class level | 13-week intervention  Primary school setting  5 schools at baseline  Aim: to examine whether the implementation of Assessment for Learning (AfL) strategies in PE classrooms can improve FMS proficiency in jumping, hopping, skipping, catching, dribbling, and overhand throwing.  PA/SB targeted outside of school time - No | 276 students at baseline  Students’ mean age: 8.4yrs (±0.6) | Teacher role: to integrate the AfL strategies in their prescribed fundamental movement skills curriculum content for 550 min of PE class time.  6 hours of teacher training  Any rationale/theory used to inform training?  Yes - the Competence Motivation Theory (Harter 1978) underpinned the design of the A+FMS intervention.  4 BCTs identified  ✓ Teacher fidelity data reported | X eligible PA data not reported | X eligible SB data not reported |
| SPACE for physical activity^8^  Christiansen et al  2017  Denmark | Cluster RCT  School level | 12-month intervention  Primary and secondary school settings  14 schools at baseline  Aim: multicomponent school-based intervention study aimed at improving PA levels among adolescents  PA/SB targeted outside of school time - Yes | 1,348 students at baseline  Students’ mean age: 12.5yrs (±0.6) | Teacher role: to facilitate and motivate PA during recess, and a mandatory outdoor recess and/ or access to gym/sports hall  22 hours of teacher training  Any rationale/theory used to inform training?  Yes - three of the intervention components involved some degree of teacher training. The rationale for the kick-starters education was initiation and organisation of recess activities. There were no underpinning theory except the rationale of offering/showing more opportunities for the students during recess. The rationale for coordinators of the Students-play-patrol, was to help older students initiating play and games for minor students. The rationale for teen-fitness instructors was initiation of fitness for the young age group primarily after school.  1 BCT identified  X No eligible fidelity data reported | ✓ PA data reported  Device used: ActiGraph  Cut points: Evenson 2008  Overall PA - counts/minute during teacher period  24-month FU after baseline | ✓ SB data reported  Device used: ActiGraph  Cut points: Evenson 2008  Sedentary mins per day (any day, including weekends)  24-month FU after baseline |
| Supporting Children’s Outcomes using Rewards, Exercise and Skills (SCORES)^9^  Cohen et al  2015  Australia | Cluster RCT  School level | 12-month intervention  Primary school setting  8 schools at baseline  Aim: to increase PA and improve fundamental movement skills competency among children attending primary schools in low-income communities  PA/SB targeted outside of school time - Yes | 460 students at baseline  Students’ mean age: 8.5yrs (±0.6) | Teacher role: to implement six PA policies to support the promotion of PA and fundamental movement skills competency within the school  7 hours of teacher training  Any rationale/theory used to inform training?  Yes - the competence motivation theory and the self-determination  theory  3 BCTs identified  ✓ Teacher fidelity data reported | ✓ PA data reported  Device used: ActiGraph  Cut points: Evenson 2008  MVPA mins in school hours  12-month FU after baseline | X eligible SB data not reported |
| No specific trial name^10^  Drummy et al  2016  United Kingdom | Cluster RCT  Class level | 12-week intervention  Primary school setting  7 schools at baseline  Aim: to improve PA levels and maintain BMI  PA/SB targeted outside of school time - No | 120 students at baseline  Students’ mean age: 9.5yrs (±not reported) | Teacher role: to lead a 5-min activity break three times per day for 12 weeks.  Hours of teacher training not reported  Theory/rationale used to inform training?  No  2 BCTs identified  X No eligible fidelity data reported | ✓ PA data reported  Device used: ActiGraph  Cut points: Trost 1998  MVPA mins per weekday  12-week FU after baseline | X eligible SB data not reported |
| The Healthy Homework Study^11^  Duncan et al  2019  New Zealand | Cluster RCT  School level | 8-week intervention  Primary school setting  16 schools at baseline  Aim: to promote physical activity and healthy eating in children  PA/SB targeted outside of school time - Yes | 675 students at baseline  Students’ mean age: 8.7yrs (±1.0) | Teacher role: to implement in-class exercises for three 1.5-h sessions delivered on different days throughout each week (including one session reviewing the previous week’s homework)  3.5 hours of teacher training  Any rationale/theory used to inform training?  Yes - the Control Theory  2 BCTs identified  X No eligible fidelity data reported | ✓ PA data reported  Device used: pedometer  Cut points: not reported  Overall PA steps/day during school hours  8-week FU after baseline | X eligible SB data not reported |
| The Active School Study^12^  Dyrstad  2018  Norway | Cluster RCT  School level | 10-month intervention  Primary school setting  9 schools at baseline  Aim: to increase children’s physical activity levels in school  PA/SB targeted outside of school time - Yes | 449 students at baseline  Students’ mean age: not reported | Teacher role: to deliver at least two 45-min physically active academic lessons per week, direct one daily 10-min physically active recess, and assign a daily 10-min physically active homework (e.g., jumping rope, running, strength training)  17.5 hours of teacher training  Any rationale/theory used to inform training?  Yes - implementation theory (Durlak & Dupre 2008)  6 BCTs identified  ✓ Teacher fidelity data reported | ✓ PA data reported  Device used: ActiGraph  Cut points: Evenson 2008  MVPA mins per day (any day, including weekends)  10-month FU after baseline | ✓ SB data reported  Device used: ActiGraph  Cut points: Evenson 2008  Sedentary mins per day (any day, including weekends)  10-month FU after baseline |
| No specific trial name^13^  Escriva-Boulley et al  2018  France | Cluster RCT  School level | 8-month intervention  Primary school setting  13 schools at baseline  Aim: to assess whether professional development would have a positive impact on teachers’ motivating style and on their students’ MVPA  PA/SB targeted outside of school time - No | 293 students at baseline  Students’ mean age: 8.3yrs (±1.1) | Teacher role: to increase their need-supportive motivating style within PE delivery  12 hours of teacher training  Any rationale/theory used to inform training?  Yes - the self-determination theory; video footages of PE teaching were used to illustrate each component of the teachers’ motivating style because Tessier et al. (2010) showed that teachers implemented more need-supportive strategies after they observed their own motivating style from video footages of their own lessons.  6 BCTs identified  ✓ Teacher fidelity data reported | X eligible PA data not reported | X eligible SB data not reported |
| Fortaleça sua Saúde^14^  Filho et al  2016  Brazil | Cluster RCT  School level | 4-month intervention  Primary school setting  6 schools at baseline  Aim: to promote active and healthy lifestyles among students from schools in low Human Development Index areas  PA/SB targeted outside of school time - Yes | 1,182 students at baseline  Students’ mean age: not reported | PE teachers  Teacher role: to conduct lessons on different health issues, to structure predominantly active PE classes, even in classes with a theoretical content, to promote opportunities for PA practice within the school and to encourage it during out-of-school time, as well as to disseminate information on the importance of an active and healthy lifestyle  18 hours of teacher training  Any rationale/theory used to inform training?  Yes - all programme content was designed based on the Health Promoting Schools framework (which included curriculum elements as one of the pillars of the intervention).  8 BCTs identified  ✓ Teacher fidelity data reported | X eligible PA data not reported | X eligible SB data not reported |
| Choice, Control and Change^15^  Gray et al  2015  United States | Cluster RCT  School level | 8-10 week intervention  Middle school setting  10 schools at baseline  Aim: to impact middle school students’ energy balance-related behaviours: eating more fruits and vegetables, drinking more water, increasing physical activity and decreasing intakes of sweetened beverages and packaged snacks, eating at fast food restaurants and leisure screen time  PA/SB targeted outside of school time - No | 1,136 students at baseline  Students’ mean age: 12.0yrs (SD not reported) | Teacher role: to deliver 24 lessons in science classes most school days over 8-10 weeks  6 hours of teacher training  Any rationale/theory used to inform training?  Yes - the rationale/theory for the teacher training was the same as it was for the curriculum so the teachers could experience the theory-based determinants as the students would. This was a combination of social cognitive theory and Self-determination theory.  4 BCTs identified  ✓ Teacher fidelity data reported | X eligible PA data not reported | X eligible SB data not reported |
| No specific trial name^16^  Ha et al  2017  Hong Kong | Cluster RCT  Class level | 4-week intervention  Secondary school setting  12 schools at baseline  Aim: to design an intervention which students, especially girls, would find interesting as well as increase activity levels  PA/SB targeted outside of school time - No | 731 students at baseline  Students’ mean age: 14.4yrs (±1.1) | Teacher role: to add a 15-min rope skipping activity to PE at the start of four consecutive PE lessons  4 hours of teacher training  Any rationale/theory used to inform training?  Yes - the Self-determination theory  6 BCTs identified  ✓ Teacher fidelity data reported | ✓ PA data reported  Device used: ActiGraph  Cut points: Evenson 2008  % of time in MVPA during teacher period  2-week FU after baseline | X eligible SB data not reported |
| Self-determined Exercise and Learning For FITness (SELF-FIT)^17^  Ha et al  2020  Hong Kong | Cluster RCT  School level | 4-month intervention  Secondary school setting  26 schools at baseline  Aim: to increase students’ MVPA during school PE  PA/SB targeted outside of school time - No | 667 students at baseline  Students’ mean age: 14.4yrs (±0.8) | Teacher role: to be more need supportive, use music to enhance enjoyment of PE lessons and to enhance the instruction methods for fitness activities that already existed in the curriculum  7 hours of teacher training  Any rationale/theory used to inform training?  Yes - based on tenets of the Self-determination theory  9 BCTs identified  ✓ Teacher fidelity data reported | ✓ PA data reported  Device used: ActiGraph  Cut points: Evenson 2008  MVPA mins during teacher period  4-month FU after baseline | ✓ SB data reported  Device used: ActiGraph  Cut points: Evenson 2008  Sedentary mins during teacher period  4-month FU after baseline |
| Girls Active^18^  Harrington et al  2018  United Kingdom | Cluster RCT  School level | 14-month intervention  Secondary school setting  20 schools at baseline  Aim: to empower adolescent girls to influence school decisions, develop themselves as role models, and promote PA to peers  PA/SB targeted outside of school time - Yes | 1,752 students at baseline  Students’ mean age: 12.8yrs (±0.8) | Teacher role: to review PA in school, sport and PE provision, culture and practice to girls  7 hours of teacher training  Any rationale/theory used to inform training?  No - (the elements of the “off-the-shelf” programme were mapped to constructs in social cognitive theory post-hoc by the academic team)  6 BCTs identified  X No eligible fidelity data reported | ✓ PA data reported  Device used: accelerometer  Cut points: Hildebrand  MVPA mins during school hours  14-month FU after baseline | ✓ SB data reported  Device used: accelerometer  Cut points: Hildebrand  Sedentary mins during school hours  14-month FU after baseline |
| No specific trial name^19^  Have et al  2018  Denmark | Cluster RCT  School level | 9-month intervention  Primary school setting  12 schools at baseline  Aim: to investigate the effect on mathematical achievement of incorporating physical activity in math teaching for 7-year-old schoolchildren  PA/SB targeted outside of school time - No | 505 students at baseline  Students’ mean age: 7.2yrs (±0.3) | Teacher role: to integrate active math into the curriculum for mathematics for one school year  28 hours of teacher training  Any rationale/theory used to inform training?  Not reported  4 BCTs identified  ✓ Teacher fidelity data reported | ✓ PA data reported  Device used: ActiGraph  Cut points: Evenson 2008  Overall PA counts/min during teacher period  24-month FU after baseline | X eligible SB data not reported |
| Academic Achievement and Physical Activity Across the Curriculum (A + PACC)^20^  Hillman et al  2017  United States | Cluster RCT  School level | 36-month intervention  Primary school setting  17 schools at baseline  Aim: to provide increased MVPA while maintaining academic instruction time  PA/SB targeted outside of school time - No | 698 students at baseline  Students’ mean age: 8.1yrs (±0.6) | Teacher role: to deliver two, 10-min lessons per day (~4-5 METs) in the subject of their choice; one in the morning and one in the afternoon, 5 days per week, 100 min/week  14 hours of teacher training  Any rationale/theory used to inform training?  Yes - the Social cognitive theory with emphasis on teacher self-efficacy  6 BCTs identified  ✓ Teacher fidelity data reported | X eligible PA data not reported | X eligible SB data not reported |
| Knowledge in Action (KIA)^21^  Hodges et al  2016  United States | RCT  Class level | 7-week intervention  Primary school setting  10 schools at baseline  Aim: to investigate the effectiveness of the Knowledge in Action (KIA) fitness lesson segments on Health Related Fitness Knowledge (i.e., a student learning outcome) among fifth grade students in physical education  PA/SB targeted outside of school time - No | 633 students at baseline  Students’ mean age: 10.1yrs (±0.5) | Teacher role: to implement the KIA fitness during the fitness segment of their 5th grade physical education classes  Hours of teacher training not reported  Any rationale/theory used to inform training?  Not reported  4 BCTs identified  ✓ Teacher fidelity data reported | X eligible PA data not reported | X eligible SB data not reported |
| ‘Physical Activity 4 Everyone’ (PA4E1)^22^  Hollis et al  2016  Australia | Cluster RCT  School level | 21.5m intervention  Secondary school setting  10 schools at baseline  Aim: to assess the effectiveness of a multi-component school-based intervention in reducing the decline in physical activity among students attending secondary schools located in disadvantaged communities  PA/SB targeted outside of school time - Yes | 1,233 students at baseline  Students’ mean age: 12.0yrs (SD not reported) | Teacher role: to implement strategies to maximise student activity levels within Physical Education classes  Hours of teacher training not reported  Any rationale/theory used to inform training?  Not reported  10 BCTs identified  ✓ Teacher fidelity data reported | ✓ PA data reported  Device used: ActiGraph  Cut points: Evenson 2008  MVPA mins per day (any day, including weekends)  24-month FU after baseline | X eligible SB data not reported |
| PLAYgrounds^23^  Janssen et al  2015  The Netherlands | Prospective controlled trial  School level | 10-month intervention  Primary school setting  8 schools at baseline  Aim: to increase the intensity of PA, focusing on the morning recess  PA/SB targeted outside of school time - Yes | 1,486 students at baseline  Students’ mean age: 8.7yrs (±1.5) | Teacher role: to supervise recess, actively encourage PA, and modify PE content  7.13 hours of teacher training  Any rationale/theory used to inform training?  Yes - the social-ecological model of behaviour change (total programme). Specific for teacher training; logic model of change  9 BCTs identified  ✓ Teacher fidelity data reported | ✓ PA data reported  Device used: ActiGraph  Cut points: Trost 1998  Overall PA - energy expenditure (kcal/kg/min) during teacher period  19 sampling moments  (every 2 weeks) | X eligible SB data not reported |
| The COPE (Creating Opportunities for Personal Empowerment) Healthy Lifestyles TEEN (Thinking, Emotions, Exercise, Nutrition) Program ^24^  Kelly et al  2015  United States | RCT  School level | 15-week intervention  Secondary school setting  11 schools at baseline  Aim: to empower teens to engage in healthy lifestyle behaviours (nutrition, physical activity, positive strategies to cope with stress, problem-solving, regulation of negative mood and goal setting)  PA/SB targeted outside of school time - Yes | 779 students at baseline  Students’ mean age: 14.7yrs (±0.7) | Teacher role: to implement COPE 1 day per week during their regular scheduled health class (50 min in length)  7 hours of teacher training  Any rationale/theory used to inform training?  No  5 BCTs identified  ✓ Teacher fidelity data reported | X eligible PA data not reported | X eligible SB data not reported |
| Resistance Training for Teens^25^  Kennedy et al  2018  Australia | Cluster RCT  School level | 10-week intervention  Secondary school setting  16 schools at baseline  Aim: to improve muscular fitness and provide adolescents with the knowledge, motivation, and skills to engage in resistance training  PA/SB targeted outside of school time - Yes | 607 students at baseline  Students’ mean age: 14.1yrs (±0.5) | Teacher role: to deliver student seminars and a structured PE programme, to facilitate a minimum of 5 lunchtime sessions over the 10-wk intervention period  5 hours of teacher training  Any rationale/theory used to inform training?  Yes - the Self-determination theory, the Social cognitive theory, and recommendations from Su and Reeve (2011) to support autonomy and task engagement, through both knowledge and skill-based training practices.  8 BCTs identified  ✓ Teacher fidelity data reported | ✓ PA data reported  Device used: GENEActiv accelerometer  Cut points: Phillips 2013  MVPA mins per weekday  3-month FU after baseline | X eligible SB data not reported |
| Classes in Motion (Bewegte Klasse)^26^  Kien et al  2018  Austria | Cluster RCT  Class level | 18-month intervention  Primary school setting  45 schools at baseline  Aim: to improve the pupils’ positive emotional and social school experience, increase their levels of physical activity, foster their motoric skills, and improve their well-being and attention  PA/SB targeted outside of school time - No | 800 students at baseline  Students’ mean age: 8.7yrs (±0.4) | Teacher role: to include active breaks in the regular curriculum and to include curriculum-focused active breaks that also comprise academic content  32 hours of teacher training  Any rationale/theory used to inform training?  No theory underpinned the teacher training. Although the rationale was that knowledge about the intervention and the capability of carrying out the intervention as well as a positive attitude towards the intervention should improve the implementation of the intervention.  2 BCTs identified  ✓ Teacher fidelity data reported | X eligible PA data not reported | X eligible SB data not reported |
| Extra Fit! (EF!)^27^  Kocken et al  2016  The Netherlands | Cluster RCT  School level | 24-month intervention  Primary school setting  45 schools at baseline  Aim: to prevent or reduce overweightness in primary school children, decrease consumption of high-energy or high-fat foods and sugar-sweetened drinks; promoting a healthy breakfast; increase consumption of fruits and vegetables; reduce television viewing and computer gaming/browsing; and increase physical activities at school and outside school hours  PA/SB targeted outside of school time - Yes | 1,112 students at baseline  Students’ mean age: 9.2yrs (±0.6 ) | Teacher role: to deliver EF! Lessons; a variety of theory and practical lessons on nutrition and physical activity  Hours of teacher training not reported  Any rationale/theory used to inform training?  Yes - the theory of planned behaviour as this was the framework on which the curriculum was based  1 BCT identified  ✓ Teacher fidelity data reported | ✓ PA data reported  Device used: ActiGraph  Cut points: Freedson 1998/Trost 2000  MVPA mins per day (any day, including weekends)  24-month FU after baseline | X eligible SB data not reported |
| Let's Move It^28^  Koykka et al  2019  Finland | Cluster RCT  School level | 2-month intervention  Secondary school setting  6 schools at baseline  Aim: to promote physical activity and reduce sedentary behaviour among older adolescents  PA/SB targeted outside of school time - Yes | 1,166 students at baseline  Students’ mean age: 18.5yrs (SD not reported) | Teacher role: to reduce sedentary behaviour in class rooms, to implement activity breaks  4.5 hours of teacher training  Any rationale/theory used to inform training?  Yes - the reasoned action approach, evidence-based strategies for habit formation  15 BCTs identified  ✓ Teacher fidelity data reported | X eligible PA data not reported | X eligible SB data not reported |
| Activity and Motivation in Physical Education (AMPED)^29^  Lonsdale et al  2019  Australia | Two-arm cluster RCT  School level | 7.5-month intervention  Secondary-level educational setting  14 schools at baseline  Aim: to maximise opportunities for students to be active during PE lessons and enhance adolescents’ motivation towards PE and PA  PA/SB targeted outside of school time - No | 1,421 students at baseline  Students’ mean age: 13.0yrs (±0.5) | Teacher role: to implement AMPED, set PA action plan, implement AMPED strategies during PE lessons, and have mentoring conversation with another teacher  23 hours of teacher training  Any rationale/theory used to inform training?  Yes - Based on previous large scale trials and tenets of the Self-determination theory  12 BCTs identified  ✓ Teacher fidelity data reported | ✓ PA data reported  Device used: ActiGraph  Cut points: Evenson 2008  % of time in MVPA during teacher period  7.5-month FU after baseline | ✓ SB data reported  Device used: ActiGraph  Cut points: Evenson 2008  Sedentary mins during teacher period  7.5-month FU after baseline |
| ‘Active Teen Leaders Avoiding Screen-time’ (ATLAS)^30^  Lubans et al  2016  Australia | Group RCT  School level | 20-week intervention  Secondary-level educational setting  14 schools at baseline  Aim: to improve boys’ self-efficacy for resistance-based exercise, by explicitly targeting resistance training movement skill competency  PA/SB targeted outside of school time - Yes | 361 students at baseline  Students’ mean age: 12.7yrs (±0.5) | Teacher role: to deliver face-to- face physical activity sessions during the school sport period (20 × ~90 min, in addition to regular PE lessons)  13 hours of teacher training  Any rationale/theory used to inform training?  Yes - the Self-determination theory and the Social cognitive theory  6 BCTs identified  ✓ Teacher fidelity data reported | ✓ PA data reported  Device used: ActiGraph  Cut points: Evenson 2008  % of time in MVPA per weekday  8-month FU after baseline | X eligible SB data not reported |
| Active Classrooms^31^  Martin et al  2017  Ireland | Cluster RCT  Class level | 8-week intervention  Primary school setting  10 schools at baseline  Aim: to educate, train and enable primary teachers to change their teaching methods toward engaging children in physical activity while learning the academic content of English and mathematics lessons  PA/SB targeted outside of school time - No | 197 students at baseline  Students’ mean age: 8.9yrs (±1.0) | Teacher role: to incorporate at least 1 active English lesson idea and 1 active mathematics lesson idea into their teaching each day  1 hour of teacher training  Any rationale/theory used to inform training?  Yes - the Capability, Opportunity, Motivation, Behaviour (COM-B) model  7 BCTs identified  X No eligible fidelity data reported | ✓ PA data reported  Device used: ActiGraph  Cut points: Evenson 2008  MVPA mins during teacher period  8-week FU after baseline | ✓ SB data reported  Device used: ActiGraph  Cut points: Evenson 2008  Sedentary mins during teacher period  8-week FU after baseline |
| Action Schools! BC (AC! BC)^32^  McKay et al  2015  Canada | Cluster RCT  School level | 20-month intervention  Primary school setting  30 schools at baseline  Aim: to provide a school environment where students had more opportunities to be more active more often, and facilitate supportive community and provincial-level environments  PA/SB targeted outside of school time - Yes | 1,529 students at baseline  Students’ mean age: not reported | Teacher role: to identify school priorities and create action plans about how to promote PA, two prescriptive, experiential components within the Classroom Action Zone: a minimum of 15 additional mins of PA (over and above physical education) per day (15×5) and jumping exercises 3×/ day (Bounce at the Bell)  4 hours of teacher training  Any rationale/theory used to inform training?  Yes - the team designed the training using their expertise in the provincial curriculum and physical education. The training followed the principles of professional development (experiential learning) and reflected common knowledge of self-efficacy theory. Through the training, the Support Team aimed to provide teachers with a positive experience learning about and engaging in physical activities with their peers.  5 BCTs identified  ✓ Teacher fidelity data reported | X eligible PA data not reported | X eligible SB data not reported |
| The Professional Learning for Understanding Games Education (PLUNGE) program^33^  Miller et al  2015  Australia | Cluster RCT  School level | 7-week intervention  Primary school setting  7 schools at baseline  Aim: to evaluate the efficacy of a game-centered learning programme for the improvement of fundamental movement skills, to evaluate the improvement of in-class PA and the potential of this approach to improve perceived sporting competence in elementary school students.  PA/SB targeted outside of school time - No | 168 students at baseline  Students’ mean age: 11.2yrs (±1.0) | Teacher role: to deliver a 7-week PE curriculum designed to promote PA  10 hours of teacher training  Any rationale/theory used to inform training?  Yes - the mentoring model and the situated learning theory  6 BCTs identified  ✓ Teacher fidelity data reported | ✓ PA data reported  Device used: Pedometer  Cut points: Scruggs 2013  Overall PA - steps/min during teacher period  8-week FU after baseline | X eligible SB data not reported |
| No specific trial name^34^  Morris et al  2019  United Kingdom | Stratified RCT  School level | 6-week intervention  Primary school setting  6 schools at baseline  Aim: to evaluate the effectiveness of a physically active learning intervention on children’s PA levels using subgroup analysis to reveal stratified intervention effects highlighted by pre-intervention PA levels  PA/SB targeted outside of school time - Yes | 154 students at baseline  Students’ mean age: 9.9yrs (±0.3) | Teacher role: to integrate movement within their lessons both within and outside of the classroom environment  4 hours of teacher training  Any rationale/theory used to inform training?  No  3 BCTs identified  X No eligible fidelity data reported | ✓ PA data reported  Device used: ActiGraph  Cut points: Evenson 2008  % of time in MVPA per day (any day, including weekends)  6-week FU after baseline | ✓ SB data reported  Device used: ActiGraph  Cut points: Evenson 2008  Sedentary mins per day (any day, including weekends)  6-week FU after baseline |
| Virtual Traveller^35^  Norris et al  2018  United Kingdom | Cluster RCT  School level | 6-week intervention  Primary school setting  10 schools at baseline  Aim: to test the effect of the “Virtual Traveller” intervention on children’s physical activity and SB, on-task behavior, and student engagement  PA/SB targeted outside of school time - No | 219 students at baseline  Students’ mean age: 8.6yrs (±0.5) | Teacher role: to deliver a programme of pre-prepared physically active lesson sessions on whiteboards  30 mins of teacher training  Any rationale/theory used to inform training?  Yes - the Capability, Opportunity, Motivation, Behaviour model  7 BCTs identified  ✓ Teacher fidelity data reported | ✓ PA data reported  Device used: Actigraph  Cut points: Evenson 2008  MVPA mins during teacher period  7-week FU after baseline | ✓ SB data reported  Device used: Actigraph  Cut points: Evenson 2008  Sedentary mins during teacher period  7-week FU after baseline |
| Generating Rural Options for Weight Healthy Kids and Communities (GROW HKC)^36^  Nader et al  2018  United States | Cluster RCT  School level | 8 -month intervention  Primary school setting  6 schools at baseline  Aim: to investigate the collective relationship of teacher-level factors with the implementation of the Balanced Energy, Physical Activity Toolkit  PA/SB targeted outside of school time - No | 1,103 students at baseline  Students’ mean age: 9.0yrs (SD not reported) | Teacher role: to implement PA breaks across the day  1.25 hours of teacher training  Any rationale/theory used to inform training?  Not reported  1 BCT identified  X No eligible fidelity data reported | X eligible PA data not reported | X eligible SB data not reported |
| Project Spraoi^37^  O Leary et al  2019  Ireland | Cluster RCT  School level | 24 -month intervention  Primary school setting  4 schools at baseline  Aim: evaluate an intervention targeting increased PA, reduced sedentary time and improved nutritional habits among primary school children  PA/SB targeted outside of school time - Yes | 231 students at baseline  Students’ mean age: not reported | Teacher role: to promote of 20 min ‘huff and puff’ (MVPA) each day, improve students’ nutritional habits and knowledge through targeted class-based activities, and promote increased habitual PA and reduced sedentary time.  88.58 hours of teacher training  (a maximum of 1.5 days a week was spent in schools over 2 year period)  Any rationale/theory used to inform training?  Yes - the social ecological model of health behaviour  9 BCTs identified  ✓ Teacher fidelity data reported | ✓ PA data reported  Device used: ActiGraph  Cut points: Evenson 2008  MVPA mins per day (any day, including weekends)  24-month FU after baseline | X eligible SB data not reported |
| Michigan Model for Health^38^  O Neill et al  2015  United States | Cluster RCT  School level | 12-week intervention  Primary school setting  52 schools at baseline  Aim: to assess a comprehensive health education curriculum focused on nutrition, physical fitness, and safety  PA/SB targeted outside of school time - No | 1,983 students at baseline  Students’ mean age: 9.6yrs (±0.7) | Teacher role: to deliver 40 min Michigan Model for Health lessons focusing on nutrition, physical fitness, and safety attitudes and skills.  14 hours of teacher training  Any rationale/theory used to inform training?  Yes - the training and support were based upon the model used by the organisation that publishes and distributes the Michigan Model for Health materials  2 BCTs identified  ✓ Teacher fidelity data reported | X eligible PA data not reported | X eligible SB data not reported |
| Girls in Sport^39^  Okely et al  2017  Australia | Cluster RCT  School level | 18 -month intervention  Secondary school setting  24 schools at baseline  Aim: to prevent a decline in participation in MVPA levels among girls over the course of the intervention  PA/SB targeted outside of school time - Yes | 1,518 students at baseline  Students’ mean age: 13.6yrs (±>0.0) | Teacher role: to try to engage girls for at least 50% of the class time in MVPA while reducing time spent in management tasks, and to promote physical activity in and out of class  48 hours of teacher training  Any rationale/theory used to inform training?  Yes - the Action Learning Framework and Quality Teaching and Learning Model  8 BCTs identified  ✓ Teacher fidelity data reported | ✓ PA data reported  Device used: ActiGraph  Cut points: Trost 2002  MVPA mins per day (any day, including weekends)  18-month FU after baseline | ✓ SB data reported  Device used: ActiGraph  Cut points: Trost 2002  Sedentary mins per day (any day, including weekends)  18 month FU after baseline |
| (Encouraging Activity to Stimulate Young Minds) EASY Minds^40^  Riley et al  2016  Australia | Cluster RCT  Class level | 6-week intervention  Primary school setting  8 schools at baseline  Aim: to evaluate the impact of a primary school-based physical activity integration programme delivered by teachers on objectively measured physical activity and key educational outcomes  PA/SB targeted outside of school time - No | 240 students at baseline  Students’ mean age: 11.1yrs (±0.7) | Teacher role: to embed movement-based learning in their students’ daily mathematics program in three lessons per week for 6-weeks  7 hours 15 mins of teacher training  Any rationale/theory used to inform training?  Yes - a diffusion of innovations model  10 BCTs identified  ✓ Teacher fidelity data reported | ✓ PA data reported  Device used: ActiGraph  Cut points: Evenson 2008  MVPA mins during teacher period  6-week FU after baseline | ✓ SB data reported  Device used: ActiGraph  Cut points: Evenson 2008  Sedentary mins during teacher period  6-week FU after baseline |
| FitQuest^41^  Robertson et al  2018  United Kingdom | Cluster RCT  School level | 5-week intervention  Primary school setting  10 schools at baseline  Aim: to evaluate whether a theory based location-based exergame (FitQuest) could increase self-efficacy and PA at school compared to standard provision in physical education (PE) classes  PA/SB targeted outside of school time - No | 10,215 students at baseline  Students’ mean age: not reported | Teacher role: to use FitQuest, a smartphone game, during at least one hour of mandated PE lessons per week  45 mins of teacher training  Any rationale/theory used to inform training?  No  2 BCTs identified  ✓ Teacher fidelity data reported | ✓ PA data reported  Device used: NL 1000 piezoelectric accelerometer  Cut points: accelerometers were set to record MVPA using the manufacturer default of 3.6 METs or above (level 4) (New Lifestyles Inc, Lee’s Summit, Missouri, USA)  MVPA mins during school hours  6-week FU after baseline | X eligible SB data not reported |
| No specific trial name^42^  Seibert et al  2019  United States | Stratified RCT  School level | 9 -month intervention  Middle school  49 schools at baseline  Aim: to assess if large-scale implementation of CDC-recommended strategies increase fitness levels of students in low SES schools compared to routine physical activity programming  PA/SB targeted outside of school time - Yes | 4,894 students at baseline  Students’ mean age: 11.1yrs (±0.1) | Teacher role: to implement 4 CDC recommended evidenced-based strategies to promote increased physical activity in schools including: (1) increasing the amount of time spent in moderate to vigorous physical activity in physical education class, (2) encouraging active classroom breaks, (3) providing organized physical activity opportunities during recess, and (4) providing organized physical activity opportunities before and after school  Hours of teacher training not reported  Any rationale/theory used to inform training?  Not reported  3 BCTs identified  ✓ Teacher fidelity data reported | X eligible PA data not reported | X eligible SB data not reported |
| Move for Wellbeing in School^43^  Smedegaard et al  2017  Denmark | Stratified RCT  School level | 12-month intervention  Primary school setting  24 schools at baseline  Aim: to improve psychosocial well-being among school-aged children and youths from 4th to 6th grade (10-13 yrs) through the development, implementation, and evaluation of a multicomponent, school-based, physical activity intervention.  PA/SB targeted outside of school time - No | 2,916 students at baseline  Students’ mean age: not reported | Teacher role: to conduct two daily brain breaks lasting five mins per class, to facilitate new activities during recess three times per week lasting 30 min; to complete three theme days focusing on well-being and PA; and to spend half of physical education classes teaching the Move for Wellbeing in School lesson plans designed for the intervention  28 hours of teacher training  Any rationale/theory used to inform training?  Yes - the self-determination theory  3 BCTs identified  ✓ Teacher fidelity data reported | X eligible PA data not reported | X eligible SB data not reported |
| No specific trial name^44^  Sutherland et al  2017  Australia | Cluster RCT  School level | 6-month intervention  Primary school setting  46 schools at baseline  Aim: to assess the implementation of school-based practices known to increase students’ moderate-to-vigorous physical activity  PA/SB targeted outside of school time - Yes | 1,139 students at baseline  Students’ mean age: not reported | Teacher role: to improve the quality of PE lessons including fundamental movement skills and increasing MVPA and supervise recess and lunch breaks at least 2 days per week  1.5 hours of teacher training  Any rationale/theory used to inform training?  Not reported  11 BCTs identified  ✓ Teacher fidelity data reported | X eligible PA data not reported | X eligible SB data not reported |
| Learning, Cognition and Motion (LCoMotion)^45^  Tarp et al  2016  Denmark | Cluster RCT  School level | 20-week intervention  Secondary school setting  14 schools at baseline  Aim: to increase physical activity levels overall as well as in school by targeting classroom, recess and leisure-time activity and through active transportation  PA/SB targeted outside of school time - Yes | 632 students at baseline  Students’ mean age: 12.9yrs (±0.6) | Teacher role: to deliver activities involving PA during academic subjects; to conduct moderate to vigorous physical activities in recess with their students  7 hours of teacher training  Any rationale/theory used to inform training?  No  5 BCTs identified  ✓ Teacher fidelity data reported | ✓ PA data reported  Device used: ActiGraph  Cut points: Evenson 2008  % of time in MVPA during teacher period  10-week FU after baseline | X eligible SB data not reported |
| The Move Project^46^  Tymms et al  2016  United Kingdom | Cluster RCT  School level | 6-week intervention  Secondary school setting  60 schools at baseline  Aim: to increase physical activity and well-being of secondary school students through education interventions  PA/SB targeted outside of school time - Yes | 1,235 students at baseline  Students’ mean age: 11.8yrs (±0.4) | **Peer mentoring:**  Teacher role: supervise peer mentoring sessions  2 hours of teacher training  Any rationale/theory used to inform training?  Not reported  1 BCT identified  ✓ Teacher fidelity data reported | **Peer mentoring:**  X eligible PA data not reported | **Peer mentoring:**  X eligible SB data not reported |
|  |  |  |  | **Participative learning:**  Teacher role: deliver lessons to help students develop an understanding of how their environment may influence their health and well-being using mapping techniques in GIS software  2 hours of teacher training  Any rationale/theory used to inform training?  Not reported  1 BCT identified  ✓ Teacher fidelity data reported | **Participative learning:**  ✓ PA data reported  Device used: ActiGraph  Cut points: Evenson 2008  MVPA mins per day (any day, including weekends)  12-week FU after baseline | **Participative learning:**  X eligible SB data not reported |
| No specific trial name^47^  van den Berg et al  2019  The Netherlands | Cluster RCT  Class level | 5-week intervention  Primary school setting  9 schools at baseline  Aim: to assess the effects of integrating juggling with math practice in primary school children, on multiplication memorisation performance and enjoyment during the math lessons  PA/SB targeted outside of school time - No | 323 students at baseline  Students’ mean age: 11.0yrs (±0.5) | Teacher role: to implement juggling maths lessons  Hours of teacher training not reported  Any rationale/theory used to inform training?  Not reported  3 BCTs identified  ✓ Teacher fidelity data reported | X eligible PA data not reported | X eligible SB data not reported |
| UP4FUN - The ENERGY Project^48^  Verloigne et al  2018  Belgium | Cluster RCT  School level | 6-month intervention  Primary and secondary school settings  19 schools at baseline  Aim: to evaluate the effect of implementing standing desks in classrooms in primary and secondary schools on pupils’ sitting-related behaviour and determinants  PA/SB targeted outside of school time - No | 322 students at baseline  Students’ mean age: 12.9yrs (±2.5) | Teacher role: to use a rotation system to make sure that all pupils had equal access to the desks, rotating pupils from the traditional desks to standing desks approximately every 25 mins  10 mins of teacher training  Any rationale/theory used to inform training?  Yes - teachers were provided with a manual and presentation based on the rationale that teachers can be considered as “agents” of pupils’ sedentary behaviour, suggesting that we focused on changing their knowledge. The aim of the materials was to provide an evidence-based rationale to implement standing desks in a classroom. Researchers wanted to make sure that the teachers saw the intervention from a health perspective (i.e. reducing sedentary behaviour).  5 BCTs identified  ✓ Teacher fidelity data reported | X eligible PA data not reported | X eligible SB data not reported |
| UP4FUN^49^  Vik et al  2015  Belgium, Germany, Greece, Hungary and Norway | Cluster RCT  School level | 6-week intervention  Primary and secondary school settings  62 schools at baseline  Aim: to reduce sitting time in school and at home, with special emphasis on television and personal computer/electronic games  PA/SB targeted outside of school time - Yes | 3,325 students at baseline  Students’ mean age: not reported  (students aged 10-12yrs) | Teacher role: to spend one school hour (45 mins) to teach a pre-planned lesson each week for 5 weeks  1.5 hours of teacher training  Any rationale/theory used to inform training?  Yes - the five steps of the Model of Planned Promotion for Population Health and framed in a socioecological  model with strategies targeting the child, family and school  5 BCTs identified  X No eligible fidelity data reported | X eligible PA data not reported | ✓ SB data reported  Device used: ActiGraph  Cut points: Evenson 2008  Sedentary mins per day (any day, including weekends)  6 week FU after baseline |
| Fueling Learning Through Exercise (FLEX)^50^  Wright et al  2019  United States | Cluster RCT  School level | 16-month intervention  Primary school setting  18 schools at baseline  Aim: to evaluate the relative impact of two programmes on children’s school-time and total daily MVPA.  PA/SB targeted outside of school time  100 Mile Club: Yes  CHALK/Just Move: No | 979 students at baseline  Students’ mean age: 8.7yrs (±0.7) | **100 Mile Club**  Teacher role: to deliver a programme that encourages children to either walk, jog, or run 100 miles over the course of the school year (approximately 3 miles per week) and to log their miles  35 mins of teacher training  Any rationale/theory used to inform training?  No  3 BCTs identified  ✓ Teacher fidelity data reported | **100 Mile Club**  X eligible PA data not reported | **100 Mile Club**  X eligible SB data not reported |
|  |  |  |  | **CHALK/Just Move**  Teacher role: to deliver a programme of structured classroom-based PA breaks that combines high- and low-intensity movements (e.g. jumping jacks, squats, yoga poses) to provide PA for children while learning.  30 mins of teacher training  Any rationale/theory used to inform training?  No  2 BCTs identified  ✓ Teacher fidelity data reported | **CHALK/Just Move**  X eligible PA data not reported | **CHALK/Just Move**  X eligible SB data not reported |
| The Childhood Health, Activity and Motor Performance Study (Chinese CHAMPS)^51^  Zhou et al  2019  China | Cluster RCT  School level | 8-month intervention  Primary and secondary school settings  12 schools at baseline  Aim: to increase the amount of time in MVPA and vigorous physical activity during school hours. In addition, nutrition education was introduced to provide students with knowledge of healthy eating.  PA/SB targeted outside of school time - No | 680 students at baseline  Students’ mean age: 12.7yrs (±0.6) | **School Physical Education (SPE) intervention** (afterschool programme intervention (ASP) not assessed as after school teacher behaviour targeted)  Teacher role: to implement a modified PE programme  14 hours of teacher training  Any rationale/theory used to inform training?  Yes - the Social Cognitive Theory/ the Theory of Planned Behaviour  9 BCTs identified  ✓ Teacher fidelity data reported | ✓ PA data reported  Device used: ActiGraph  Cut points: Evenson 2008  % of time in MVPA during school hours  8-month FU after baseline | ✓ SB data reported  Device used: ActiGraph  Cut points: Evenson 2008  Sedentary mins per weekday  8-month FU after baseline |

m=months; FU=follow-up; PA= physical activity; SB=sedentary behaviour; BCTs=behaviour change techniques; n=number; SD=standard deviation; mins=minutes; MVPA=moderate to vigorous intensity physical activity; BMI= body mass index; PE=physical education; not reported=indicates information could not be identified from study outputs or information was not provided by study authors in the case of teacher training details

**Study outputs and sources identified within data extraction period**

**^1^ Aadland et al., ASK**

Aadland, K. N., Ommundsen, Y., Anderssen, S. A., Brønnick, K. S., Moe, V. F., Resaland, G. K., ... & Aadland, E. (2019). Effects of the Active Smarter Kids (ASK) physical activity school-based intervention on executive functions: a cluster-randomized controlled trial. *Scandinavian Journal of Educational Research*, *63*(2), 214-228.

Resaland, G. K., Aadland, E., Moe, V. F., Aadland, K. N., Skrede, T., Stavnsbo, M., ... & Kvalheim, O. M. (2016). Effects of physical activity on schoolchildren's academic performance: The Active Smarter Kids (ASK) cluster-randomized controlled trial. *Preventive Medicine*, *91*, 322-328.

Stavnsbo, M., Aadland, E., Anderssen, S. A., Chinapaw, M., Steene-Johannessen, J., Andersen, L. B., & Resaland, G. K. (2020). Effects of the Active Smarter Kids (ASK) physical activity intervention on cardiometabolic risk factors in children: A cluster-randomized controlled trial. *Preventive Medicine*, *130*, 105868.

Resaland, G. K., Moe, V. F., Aadland, E., Steene-Johannessen, J., Glosvik, Ø., Andersen, J. R., ... & Anderssen, S. A. (2015). Active Smarter Kids (ASK): Rationale and design of a cluster-randomized controlled trial investigating the effects of daily physical activity on children’s academic performance and risk factors for non-communicable diseases. *BMC public health*, *15*(1), 1-10.

<https://www.clinicaltrials.gov/ct2/show/NCT02132494?term=NCT02132494&draw=2&rank=1>

**^2^ Adab et al., WAVES**

**References:**

Adab, P., Pallan, M. J., Lancashire, E. R., Hemming, K., Frew, E., Barrett, T., ... & Daley, A. (2018). Effectiveness of a childhood obesity prevention programme delivered through schools, targeting 6 and 7 year olds: cluster randomised controlled trial (WAVES study). *bmj*, *360*.

Adab, P., Pallan, M. J., Lancashire, E. R., Hemming, K., Frew, E., Griffin, T., ... & Deeks, J. (2015). A cluster-randomised controlled trial to assess the effectiveness and cost-effectiveness of a childhood obesity prevention programme delivered through schools, targeting 6-7 year old children: the WAVES study protocol. *BMC Public Health*, *15*(1), 488.

Griffin, T. L., Clarke, J. L., Lancashire, E. R., Pallan, M. J., Passmore, S., & Adab, P. (2015). Teacher experiences of delivering an obesity prevention programme (The WAVES study intervention) in a primary school setting. *Health Education Journal*, *74*(6), 655-667.

Adab, P., Barrett, T., Bhopal, R., Cade, J. E., Canaway, A., Cheng, K. K., ... & Ekelund, U. (2018). The West Midlands ActiVe lifestyle and healthy Eating in School children (WAVES) study: a cluster randomised controlled trial testing the clinical effectiveness and cost-effectiveness of a multifaceted obesity prevention intervention programme targeted at children aged 6-7 years. *Health Technology Assessment (Winchester, England)*, *22*(8), 1.

Griffin, T. L., Clarke, J. L., Lancashire, E. R., Pallan, M. J., Adab, P., & WAVES study trial investigators. (2017). Process evaluation results of a cluster randomised controlled childhood obesity prevention trial: the WAVES study. *BMC Public Health*, *17*(1), 681.

<http://www.isrctn.com/ISRCTN97000586>

**^3^ Aittasalo et al., KIDS OUT!**

**References:**

Aittasalo, M., Jussila, A. M., Tokola, K., Sievänen, H., Vähä-Ypyä, H., & Vasankari, T. (2019). Kids Out; evaluation of a brief multimodal cluster randomized intervention integrated in health education lessons to increase physical activity and reduce sedentary behavior among eighth graders. *BMC public health*, *19*(1), 415.

Jussila, A. M., Vasankari, T., Paronen, O., Sievänen, H., Tokola, K., Vähä-Ypyä, H., ... & Aittasalo, M. (2015). KIDS OUT! Protocol of a brief school-based intervention to promote physical activity and to reduce screen time in a sub-cohort of Finnish eighth graders. *BMC public health*, *15*(1), 634.

<https://clinicaltrials.gov/ct2/show/NCT01633918?term=NCT01633918&rank=1>

**^4^ Anderson et al., AFLY5**

**References:**

Anderson, E. L., Howe, L. D., Kipping, R. R., Campbell, R., Jago, R., Noble, S. M., ... & Lawlor, D. A. (2016). Long-term effects of the Active for Life Year 5 (AFLY5) school-based cluster-randomised controlled trial. *BMJ open*, *6*(11).

Lawlor, D. A., Jago, R., Noble, S. M., Chittleborough, C. R., Campbell, R., Mytton, J., ... & Kipping, R. R. (2011). The Active for Life Year 5 (AFLY5) school based cluster randomised controlled trial: study protocol for a randomized controlled trial. *Trials*, *12*(1), 181.

Kipping, R. R., Howe, L. D., Jago, R., Campbell, R., Wells, S., Chittleborough, C. R., ... & Lawlor, D. A. (2014). Effect of intervention aimed at increasing physical activity, reducing sedentary behaviour, and increasing fruit and vegetable consumption in children: active for Life Year 5 (AFLY5) school based cluster randomised controlled trial. *Bmj*, *348*, g3256.

Lawlor, D. A., Kipping, R. R., Anderson, E. L., Howe, L. D., Chittleborough, C. R., Moure-Fernandez, A., ... & Jago, R. (2016). Active for Life Year 5: a cluster randomised controlled trial of a primary school-based intervention to increase levels of physical activity, decrease sedentary behaviour and improve diet.

Campbell, R., Rawlins, E., Wells, S., Kipping, R. R., Chittleborough, C. R., Peters, T. J., ... & Jago, R. (2015). Intervention fidelity in a school-based diet and physical activity intervention in the UNITED KINGDOM: Active for Life Year 5. *International Journal of Behavioral Nutrition and Physical Activity*, *12*(1), 141.

[**http://www.isrctn.com/ISRCTN50133740**](http://www.isrctn.com/ISRCTN50133740)

**^5^ Belton et al., Y-PATH**

**References:**

Belton, S., McCarren, A., McGrane, B., Powell, D., & Issartel, J. (2019). The Youth-Physical Activity Towards Health (Y-PATH) intervention: Results of a 24 month cluster randomised controlled trial. *PloS one*, *14*(9), e0221684.

Belton, S., O’Brien, W., Meegan, S., Woods, C., & Issartel, J. (2014). Youth-physical activity towards health: Evidence and background to the development of the Y-PATH physical activity intervention for adolescents. *BMC Public Health*, *14*(1), 122.

McGrane, B., Belton, S., Fairclough, S. J., Powell, D., & Issartel, J. (2018). Outcomes of the Y-PATH Randomized controlled trial: can a school-based intervention improve fundamental movement skill proficiency in adolescent youth?. *Journal of Physical Activity and Health*, *15*(2), 89-98.

Belton, S., O’Brien, W., McGann, J., & Issartel, J. (2019). Bright spots physical activity investments that work: Youth-Physical Activity Towards Health (Y-PATH). *British journal of sports medicine*, *53*(4), 208-212.

<http://www.isrctn.com/ISRCTN20495704>

**^6^ Bundy et al., Sydney Playground Project**

**References:**

Bundy, A., Engelen, L., Wyver, S., Tranter, P., Ragen, J., Bauman, A., ... & Perry, G. (2017). Sydney playground project: a cluster‐randomized trial to increase physical activity, play, and social skills. *Journal of school health*, *87*(10), 751-759.

Bundy, A. C., Naughton, G., Tranter, P., Wyver, S., Baur, L., Schiller, W., ... & Niehues, A. (2011). The Sydney playground project: popping the bubblewrap-unleashing the power of play: a cluster randomized controlled trial of a primary school playground-based intervention aiming to increase children's physical activity and social skills. *BMC public health*, *11*(1), 680.

Niehues, A. N., Bundy, A., Broom, A., Tranter, P., Ragen, J., & Engelen, L. (2013). Everyday uncertainties: reframing perceptions of risk in outdoor free play. *Journal of Adventure Education & Outdoor Learning*, *13*(3), 223-237.

Bundy, A. C., Naughton, G., Tranter, P., Wyver, S., Baur, L., Schiller, W., . . . Brentnall, J. (2011). The Sydney playground project: popping the bubblewrap - unleashing the power of play: a cluster randomized controlled trial of a primary school playground-based intervention aiming to increase children's physical activity and social skills. *BMC Open Access, 11*, 1-9.

Bundy, A. C., Wyver, S., Beetham, K. S., Ragen, J., Naughton, G., Tranter, P., . . . Sterman, J. (2015). The Sydney playground project - levelling the playing field: A cluster trial of a primary school-based intervention aiming to promote manageable risk-taking in children with disability. *BMC Public Health*, 1-6.

Engelen, L., Bundy, A. C., Naughton, G., Simpson, J. M., Bauman, A., Ragen, J., . . . van der Ploeg, H. P. (2013). Increasing physical activity in young primary school children - it's child's play: A cluster randomised controlled trial. *Preventive Medicine, 56*(5), 319-325.

Grady-Dominguez, P., Ihrig, K., Lane, S., Aberle, J., Beetham, K., Ragen, J., . . . Bundy, A. (2020). Reframing risk: Working with caregivers of children with disabilities to promote risk-taking in play. International Review of Research in Developmental Disabilities, 59, 1-45. doi:https://doi.org/10.1016/bs.irrdd.2020.09.001

Niehues, A. N., Bundy, A., Broom, A., & Tranter, P. (2015). Parents' perception of risk and the influence on children's everday activities. *Journal of Child and Family Studies, 24*, 809-820.

Niehues, A., Bundy, A., Broom, A., & Tranter, P. (2016). Reframing healthy risk taking: Parents’ dilemmas and strategies to promote children’s well-being. *Journal of Occupational Science, 23*(4), 449-463.

Sterman, J., Villenueve, M., Spencer, G., Wyver, S., Beetham, K., Naughton, G., . . . Bundy, A. (2020). Creating play opportunities on the school playground: Educator experiences of the Sydney Playground Project. *Occupational Therapy Journal of Australia, 67*(1), 62-73.

<http://www.anzctr.org.au/Trial/Registration/TrialReview.aspx?ACTRN=12611000089932>

**^7^ Chan et al., A+FMS**

**References:**

Chan, C. H., Ha, A. S., Ng, J. Y., & Lubans, D. R. (2019). The A+ FMS cluster randomized controlled trial: An assessment-based intervention on fundamental movement skills and psychosocial outcomes in primary schoolchildren. *Journal of Science and Medicine in Sport*, *22*(8), 935-940.

Chan, C., Ha, A., & Ng, J. Y. (2016). Improving fundamental movement skills in Hong Kong students through an assessment for learning intervention that emphasizes fun, mastery, and support: the A+ FMS randomized controlled trial study protocol. *SpringerPlus*, *5*(1), 724.

<https://www2.ccrb.cuhk.edu.hk/registry/public/318>

**^8^ Christiansen et al., SPACE**

**References:**

Christiansen, L. B., Toftager, M., Pawlowski, C. S., Andersen, H. B., Ersbøll, A. K., & Troelsen, J. (2017). Schoolyard upgrade in a randomized controlled study design—how are school interventions associated with adolescents’ perception of opportunities and recess physical activity. *Health Education Research*, *32*(1), 58-68.

Toftager, M., Christiansen, L. B., Kristensen, P. L., & Troelsen, J. (2011). SPACE for physical activity-a multicomponent intervention study: study design and baseline findings from a cluster randomized controlled trial. *BMC Public Health*, *11*(1), 777.

Toftager, M., Christiansen, L. B., Ersbøll, A. K., Kristensen, P. L., Due, P., & Troelsen, J. (2014). Intervention effects on adolescent physical activity in the multicomponent SPACE study: a cluster randomized controlled trial. *PLoS One*, *9*(6), e99369.

*Space - rum til fysisk aktivitet. Samlet evaluering af en helhedsorienteret, forebyggende indsats for børn og unge, januar 2014. Af Jens Troelsen (red.), Lars Breum Christiansen, Mette Toftager, Else Olesen, Betina Højgaard, Anne Brøcker, Stinne Aaløkke Ballegaard, Lone Grøn, Louise Ladegaard, Thomas Tjørnelund Nielsen og Brian Linke* <https://www.sdu.dk/sif/-/media/images/sif/udgivelser/2014/space_rum_til_fysisk_aktivitet.pdf>

<http://www.isrctn.com/ISRCTN79122411?q=ISRCTN79122411&filters=&sort=&offset=1&totalResults=1&page=1&pageSize=10&searchType=basic-search>

<http://www.cirhp.dk/> - not accessible

[http://www.forebyggelsescenter.dk/side.asp?side=8&id=12&ver=United Kingdom](http://www.forebyggelsescenter.dk/side.asp?side=8&id=12&ver=uk) - not accessible

**^9^ Cohen et al., SCORES**

**References:**

Cohen, K. E., Morgan, P. J., Plotnikoff, R. C., Callister, R., & Lubans, D. R. (2015). Physical activity and skills intervention: SCORES cluster randomized controlled trial. *Medicine and science in sports and exercise*, *47*(4), 765-774.

Lubans, D. R., Morgan, P. J., Weaver, K., Callister, R., Dewar, D. L., Costigan, S. A., ... & Plotnikoff, R. C. (2012). Rationale and study protocol for the supporting children’s outcomes using rewards, exercise and skills (SCORES) group randomized controlled trial: A physical activity and fundamental movement skills intervention for primary schools in low-income communities. *BMC public health*, *12*(1), 427.

Cohen, K. E., Morgan, P. J., Plotnikoff, R. C., Hulteen, R. M., & Lubans, D. R. (2017). Psychological, social and physical environmental mediators of the SCORES intervention on physical activity among children living in low-income communities. *Psychology of Sport and Exercise*, *32*, 1-11.

<https://www.anzctr.org.au/Trial/Registration/TrialReview.aspx?id=343424>

**^10^ Donnelly et al., A + PACC**

**References:**

Donnelly, J. E., Hillman, C. H., Greene, J. L., Hansen, D. M., Gibson, C. A., Sullivan, D. K., ... & Herrmann, S. D. (2017). Physical activity and academic achievement across the curriculum: results from a 3-year cluster-randomized trial. *Preventive medicine*, *99*, 140-145.

Donnelly, J. E., Greene, J. L., Gibson, C. A., Sullivan, D. K., Hansen, D. M., Hillman, C. H., ... & Herrmann, S. D. (2013). Physical activity and academic achievement across the curriculum (A+ PAAC): rationale and design of a 3-year, cluster-randomized trial. *BMC public health*, *13*(1), 1-8.

Szabo-Reed, A. N., Willis, E. A., Lee, J., Hillman, C. H., Washburn, R. A., & Donnelly, J. E. (2019). The influence of classroom physical activity participation and time on task on academic achievement. *Translational Journal of the American College of Sports Medicine*, *4*(12), 84-95.

Szabo-Reed, A. N., Willis, E. A., Lee, J., Hillman, C. H., Washburn, R. A., & Donnelly, J. E. (2017). Impact of 3 years of classroom physical activity bouts on time-on-task behavior. *Medicine and science in sports and exercise*, *49*(11), 2343.

<https://clinicaltrials.gov/ct2/show/NCT01699295>

**^11^ Drummy et al., Unnamed**

**References:**

Drummy, C., Murtagh, E. M., McKee, D. P., Breslin, G., Davison, G. W., & Murphy, M. H. (2016). The effect of a classroom activity break on physical activity levels and adiposity in primary school children. *Journal of paediatrics and child health*, *52*(7), 745-749.

**^12^ Duncan et al., Healthy Homework study**

**References:**

Duncan, S., Stewart, T., McPhee, J., Borotkanics, R., Prendergast, K., Zinn, C., ... & Schofield, G. (2019). Efficacy of a compulsory homework programme for increasing physical activity and improving nutrition in children: a cluster randomised controlled trial. *International Journal of Behavioral Nutrition and Physical Activity*, *16*(1), 80.

[*http://www.anzctr.org.au/Trial/Registration/TrialReview.aspx?ACTRN=12618000590268*](http://www.anzctr.org.au/Trial/Registration/TrialReview.aspx?ACTRN=12618000590268)

Duncan, S., McPhee, J. C., Schluter, P. J., Zinn, C., Smith, R., & Schofield, G. (2011). Efficacy of a compulsory homework programme for increasing physical activity and healthy eating in children: the healthy homework pilot study. *International Journal of Behavioral Nutrition and Physical Activity*, *8*(1), 127.

**^13^ Dyrstad et al., the Active School Study**

**References:**

Dyrstad, S. M., Kvalø, S. E., Alstveit, M., & Skage, I. (2018). Physically active academic lessons: acceptance, barriers and facilitators for implementation. *BMC public health*, *18*(1), 1-11.

Kvalø, S. E., Bru, E., Brønnick, K., & Dyrstad, S. M. (2017). Does increased physical activity in school affect children's executive function and aerobic fitness?. *Scandinavian journal of medicine & science in sports*, *27*(12), 1833-1841.

Seljebotn, P. H., Skage, I., Riskedal, A., Olsen, M., Kvalø, S. E., & Dyrstad, S. M. (2019). Physically active academic lessons and effect on physical activity and aerobic fitness. The Active School study: A cluster randomized controlled trial. *Preventive Medicine Reports*, *13*, 183-188.

<https://clinicaltrials.gov/ct2/show/NCT03436355?term=NCT03436355&rank=1>

<https://www.activesmarterkids.com/>

Skage, I., Ertesvåg, S.K., Roland, P. & Dyrstad, S.M. (2020). Implementation of physically active lessons: A 2-year follow-up. Evaluation and Program Planning, 83, 101874.

Skage, I. & Dyrstad, S.M. (2019). “It`s not because we don`t believe in it …”: Headteachers` perceptions of implementing physically active lessons in school. *BMC Public Health*, 19, 1674.

**^14^ Escriva-Boulley et al., Unnamed**

**References:**

Escriva-Boulley, G., Tessier, D., Ntoumanis, N., & Sarrazin, P. (2018). Need-supportive professional development in elementary school physical education: Effects of a cluster-randomized control trial on teachers’ motivating style and student physical activity. *Sport, Exercise, and Performance Psychology*, *7*(2), 218.

**^15^ Filho et al., Fortaleça sua Saúde**

**References:**

Barbosa Filho, V. C., da Silva, K. S., Mota, J., Beck, C., & da Silva Lopes, A. (2016). A physical activity intervention for brazilian students from low human development index areas: a cluster-randomized controlled trial. *Journal of Physical Activity and Health*, *13*(11), 1174-1182.

Barbosa Filho, V. C., da Silva Lopes, A., Lima, A. B., de Souza, E. A., do Amaral Gubert, F., Silva, K. S., ... & Mota, J. (2015). Rationale and methods of a cluster-randomized controlled trial to promote active and healthy lifestyles among Brazilian students: the “Fortaleça sua Saúde” program. *BMC Public Health*, *15*(1), 1212.

Araújo, Thábyta & Carvalho, Queliane & Barbosa Filho, Valter & Costa, Ana & Gubert, Fabiane & Vieira, Neiva. (2016). Educação em saúde no ambiente escolar - estudo de intervenção com professores da rede pública. Revista Tendências da Enfermagem Profissional - ReTEP. 8. 2024.

Souza, Evanice & Castro, Ângela & Sousa, Antônia & Alves, Felipe. (2016). Physical Education classes in the Empower your Health Project: an analysis from the standpoint of Teachers. Journal of Physical Activity and Health. 21. 198-206.

Lopes, Iraneide & Linard, Jair & Silva, Magna & Barbosa Filho, Valter. (2020). IMPLEMENTAÇÃO DO PROGRAMA DE PROMOÇÃO DO ESTILO DE VIDA ATIVO EM ESTUDANTES: O "FORTALEÇA SUA SAÚDE. 31. 14. 10.4025/jphyseduc.v31i1.3125.

<https://clinicaltrials.gov/ct2/show/NCT02439827>

**^16^ Gray et al., Choice Control and Change**

**References:**

Gray, H. L., Contento, I. R., & Koch, P. A. (2015). Linking implementation process to intervention outcomes in a middle school obesity prevention curriculum,‘Choice, Control and Change’. *Health education research*, *30*(2), 248-261.

Contento, I. R., Koch, P. A., Lee, H., & Calabrese-Barton, A. (2010). Adolescents demonstrate improvement in obesity risk behaviors after completion of choice, control & change, a curriculum addressing personal agency and autonomous motivation. *Journal of the American Dietetic Association*, *110*(12), 1830-1839.

Lee, H., Contento, I. R., & Koch, P. (2013). Using a systematic conceptual model for a process evaluation of a middle school obesity risk-reduction nutrition curriculum intervention: choice, control & change. *Journal of Nutrition Education and Behavior*, *45*(2), 126-136.

**^17^ Ha et al., SELF-FIT**

**References:**

Ha, A. S., Lonsdale, C., Lubans, D. R., & Ng, J. Y. (2020). Increasing Students' Activity in Physical Education: Results of the Self-determined Exercise and Learning For FITness Trial. *Medicine and Science in Sports and Exercise*, *52*(3), 696-704.

Ha, A. S., Lonsdale, C., Lubans, D. R., & Ng, J. Y. (2018). Increasing students’ physical activity during school physical education: rationale and protocol for the SELF-FIT cluster randomized controlled trial. *BMC Public Health*, *18*(1), 1-12.

<https://www.anzctr.org.au/Trial/Registration/TrialReview.aspx?id=368560>

**^18^ Ha et al., Unnamed**

Ha, A. S., Lonsdale, C., Ng, J. Y., & Lubans, D. R. (2017). A school-based rope skipping program for adolescents: Results of a randomized trial. *Preventive medicine*, *101*, 188-194.

Ha, A. S., Lonsdale, C., Ng, J. Y., & Lubans, D. R. (2014). A school-based rope skipping intervention for adolescents in Hong Kong: protocol of a matched-pair cluster randomized controlled trial. *BMC Public Health*, *14*(1), 1-8.

<https://www.anzctr.org.au/Trial/Registration/TrialReview.aspx?id=364862>

**^19^ Harrington et al., Girls Active**

**References:**

Harrington, D. M., Davies, M. J., Bodicoat, D. H., Charles, J. M., Chudasama, Y. V., Gorely, T., ... & Edwards, R. T. (2018). Effectiveness of the ‘Girls Active’school-based physical activity programme: A cluster randomised controlled trial. *International journal of behavioral nutrition and physical activity*, *15*(1), 40.

Edwardson, C. L., Harrington, D. M., Yates, T., Bodicoat, D. H., Khunti, K., Gorely, T., ... & Davies, M. J. (2015). A cluster randomised controlled trial to investigate the effectiveness and cost effectiveness of the ‘Girls Active’intervention: a study protocol. *BMC Public Health*, *15*(1), 526.

Gorely, T., Harrington, D. M., Bodicoat, D. H., Davies, M. J., Khunti, K., Sherar, L. B., ... & Edwardson, C. L. (2019). Process evaluation of the school-based Girls Active programme. *BMC public health*, *19*(1), 1187.

<http://www.isrctn.com/ISRCTN10688342>

**^20^ Have et al., Unnamed**

**References:**

Have, M., Nielsen, J. H., Ernst, M. T., Gejl, A. K., Fredens, K., Grøntved, A., & Kristensen, P. L. (2018). Classroom-based physical activity improves children’s math achievement-A randomized controlled trial. *PloS one*, *13*(12), e0208787.

Have, M., Nielsen, J. H., Gejl, A. K., Ernst, M. T., Fredens, K., Støckel, J. T., ... & Kristensen, P. L. (2016). Rationale and design of a randomized controlled trial examining the effect of classroom-based physical activity on math achievement. *BMC Public Health*, *16*(1), 304.

<https://clinicaltrials.gov/ct2/show/NCT02488460>

**^21^ Hillman et al., A + PACC**

**References:**

Donnelly, J. E., Hillman, C. H., Greene, J. L., Hansen, D. M., Gibson, C. A., Sullivan, D. K., ... & Herrmann, S. D. (2017). Physical activity and academic achievement across the curriculum: results from a 3-year cluster-randomized trial. *Preventive medicine*, *99*, 140-145.

Donnelly, J. E., Greene, J. L., Gibson, C. A., Sullivan, D. K., Hansen, D. M., Hillman, C. H., ... & Herrmann, S. D. (2013). Physical activity and academic achievement across the curriculum (A+ PAAC): rationale and design of a 3-year, cluster-randomized trial. *BMC public health*, *13*(1), 1-8.

Szabo-Reed, A. N., Willis, E. A., Lee, J., Hillman, C. H., Washburn, R. A., & Donnelly, J. E. (2019). The influence of classroom physical activity participation and time on task on academic achievement. *Translational Journal of the American College of Sports Medicine*, *4*(12), 84-95.

Szabo-Reed, A. N., Willis, E. A., Lee, J., Hillman, C. H., Washburn, R. A., & Donnelly, J. E. (2017). Impact of 3 years of classroom physical activity bouts on time-on-task behavior. *Medicine and science in sports and exercise*, *49*(11), 2343.

<https://clinicaltrials.gov/ct2/show/NCT01699295>

**^22^ Hodges et al., KIA**

**References**

Hodges, M. G., Kulinna, P. H., Van Der Mars, H., & Lee, C. (2016). Knowledge in action: Fitness lesson segments that teach health-related fitness in elementary physical education. *Journal of teaching in physical education*, *35*(1), 16-26.

**^23^ Hollis et al., PA4E1**

**References:**

Hollis, J. L., Sutherland, R., Campbell, L., Morgan, P. J., Lubans, D. R., Nathan, N., ... & Cohen, K. E. (2016). Effects of a ‘school-based’physical activity intervention on adiposity in adolescents from economically disadvantaged communities: Secondary outcomes of the ‘Physical Activity 4 Everyone’RCT. *International Journal of Obesity*, *40*(10), 1486-1493.

Sutherland, R., Campbell, E., Lubans, D. R., Morgan, P. J., Okely, A. D., Nathan, N., ... & Wiggers, J. (2013). A cluster randomised trial of a school-based intervention to prevent decline in adolescent physical activity levels: study protocol for the ‘Physical Activity 4 Everyone’trial. *BMC Public Health*, *13*(1), 1-10.

Sutherland, R., Campbell, E., Lubans, D. R., Morgan, P. J., Okely, A. D., Nathan, N., ... & Wiggers, J. (2016). ‘Physical Activity 4 Everyone’school-based intervention to prevent decline in adolescent physical activity levels: 12 month (mid-intervention) report on a cluster randomised trial. *British journal of sports medicine*, *50*(8), 488-495.

Sutherland, R. L., Campbell, E. M., Lubans, D. R., Morgan, P. J., Nathan, N. K., Wolfenden, L., ... & Williams, A. J. (2016). The physical activity 4 everyone cluster randomized trial: 2-year outcomes of a school physical activity intervention among adolescents. *American journal of preventive medicine*, *51*(2), 195-205.

<https://www.anzctr.org.au/Trial/Registration/TrialReview.aspx?id=362315>

**^24^ Janssen et al., PLAYgrounds**

**References:**

Janssen, M., Twisk, J. W., Toussaint, H. M., van Mechelen, W., & Verhagen, E. A. (2015). Effectiveness of the PLAYgrounds programme on PA levels during recess in 6-year-old to 12-year-old children. *British journal of sports medicine*, *49*(4), 259-264.

Janssen, M., Toussaint, H. M., Van Willem, M., & Verhagen, E. A. (2011). PLAYgrounds: Effect of a PE playground program in primary schools on PA levels during recess in 6 to 12 year old children. Design of a prospective controlled trial. *BMC public health*, *11*(1), 1-6.

<https://www.trialregister.nl/trial/2260>

<https://www.nji.nl/nl/Databank/Databank-Effectieve-Jeugdinterventies/Erkende-interventies/PLAYgrounds>

[www.playgrounds.nu](http://www.playgrounds.nu)

**^25^ Kelly et al., COPE TEEN**

**References:**

Kelly, S. A., Oswalt, K., Melnyk, B. M., & Jacobson, D. (2015). Comparison of intervention fidelity between COPE TEEN and an attention-control program in a randomized controlled trial. *Health Education Research*, *30*(2), 233-247.

Melnyk, B. M., Kelly, S., Jacobson, D., Belyea, M., Shaibi, G., Small, L., ... & Marsiglia, F. F. (2013). The COPE healthy lifestyles TEEN randomized controlled trial with culturally diverse high school adolescents: baseline characteristics and methods. *Contemporary clinical trials*, *36*(1), 41-53.

**^26^ Kennedy et al., Resistance Training for Teens**

**References**

Kennedy, S. G., Smith, J. J., Morgan, P. J., Peralta, L. R., Hilland, T. A., Eather, N., ... & Dewar, D. L. (2018). Implementing resistance training in secondary schools: a cluster randomized controlled trial. *Medicine & Science in Sports & Exercise*, *50*(1), 62-72.

Kennedy, S. G., Peralta, L. R., Lubans, D. R., Foweather, L., & Smith, J. J. (2019). Implementing a school-based physical activity program: process evaluation and impact on teachers’ confidence, perceived barriers and self-perceptions. *Physical Education and Sport Pedagogy*, *24*(3), 233-248.

Lubans, D. R., Smith, J. J., Peralta, L. R., Plotnikoff, R. C., Okely, A. D., Salmon, J., ... & Hilland, T. A. (2016). A school-based intervention incorporating smartphone technology to improve health-related fitness among adolescents: rationale and study protocol for the NEAT and ATLAS 2.0 cluster randomised controlled trial and dissemination study. *BMJ open*, *6*(6).

<https://www.anzctr.org.au/Trial/Registration/TrialReview.aspx?id=368026>

**^27^ Köykkä et al., Let’s Move It**

**References:**

Köykkä, K., Absetz, P., Araújo-Soares, V., Knittle, K., Sniehotta, F. F., & Hankonen, N. (2019). Combining the reasoned action approach and habit formation to reduce sitting time in classrooms: Outcome and process evaluation of the Let's Move It teacher intervention. *Journal of Experimental Social Psychology*, *81*, 27-38.

Hankonen, N., Heino, M. T., Araujo-Soares, V., Sniehotta, F. F., Sund, R., Vasankari, T., ... & HaUnited Kingdomkala, A. (2016). ‘Let’s Move It’-a school-based multilevel intervention to increase physical activity and reduce sedentary behaviour among older adolescents in vocational secondary schools: a study protocol for a cluster-randomised trial. *BMC Public Health*, *16*(1), 451.

<https://osf.io/v94fw>

<http://www.isrctn.com/ISRCTN10979479>

**^28^ Kien et al., Bewegte Klasse**

**References:**

Kien, C., Grillich, L., Nussbaumer-Streit, B., & Schoberberger, R. (2018). Pathways leading to success and non-success: a process evaluation of a cluster randomized physical activity health promotion program applying fuzzy-set qualitative comparative analysis. *BMC public health*, *18*(1), 1386.

Grillich, L., Kien, C., Takuya, Y., Weber, M., & Gartlehner, G. (2016). Effectiveness evaluation of a health promotion programme in primary schools: a cluster randomised controlled trial. *BMC public health*, *16*(1), 679.

<https://www.drks.de/drks_web/navigate.do?navigationId=trial.HTML&TRIAL_ID=DRKS00000622>

**^29^ Kocken et al., EF!**

**References:**

Kocken, P. L., Scholten, A. M., Westhoff, E., De Kok, B. P., Taal, E. M., & Goldbohm, R. A. (2016). Effects of a theory-based education program to prevent overweightness in primary school children. *Nutrients*, *8*(1), 12.

**^30^ Lonsdale et al., AMPED**

**References:**

Lonsdale, C., Lester, A., Owen, K. B., White, R. L., Peralta, L., Kirwan, M., ... & Kolt, G. S. (2019). An internet-supported school physical activity intervention in low socioeconomic status communities: results from the Activity and Motivation in Physical Education (AMPED) cluster randomised controlled trial. *British Journal of Sports Medicine*, *53*(6), 341-347.

Lonsdale, C., Lester, A., Owen, K. B., White, R. L., Moyes, I., Peralta, L., ... & Kolt, G. S. (2015). An internet-supported physical activity intervention delivered in secondary schools located in low socio-economic status communities: study protocol for the activity and motivation in physical education (AMPED) cluster randomized controlled trial. *BMC Public Health*, *16*(1), 17.

Lubans, D. R., Beauchamp, M. R., Diallo, T. M., Peralta, L. R., Bennie, A., White, R. L., ... & Lonsdale, C. (2018). School physical activity intervention effect on adolescents’ performance in mathematics. *Med Sci Sports Exerc*, *50*(12), 2442-50.

Peralta, L. R., Bennie, A., Gore, J., & Lonsdale, C. (2020). An Investigation of the Influence of Video Types and External Facilitation on PE Inservice Teachers’ Reflections and Their Perceptions of Learning: Findings From the AMPED Cluster Controlled Trial. *Journal of Teacher Education*, 0022487120964079.

<https://www.anzctr.org.au/Trial/Registration/TrialReview.aspx?id=365660>

**^31^ Lubans et al., ATLAS**

**References:**

Lubans, D. R., Smith, J. J., Plotnikoff, R. C., Dally, K. A., Okely, A. D., Salmon, J., & Morgan, P. J. (2016). Assessing the sustained impact of a school-based obesity prevention program for adolescent boys: the ATLAS cluster randomized controlled trial. *International Journal of Behavioral Nutrition and Physical Activity*, *13*(1), 92.

Smith, J. J., Morgan, P. J., Plotnikoff, R. C., Dally, K. A., Salmon, J., Okely, A. D., ... & Lubans, D. R. (2014). Rationale and study protocol for the ‘Active Teen Leaders Avoiding Screen-time’(ATLAS) group randomized controlled trial: an obesity prevention intervention for adolescent boys from schools in low-income communities. *Contemporary Clinical Trials*, *37*(1), 106-119.

Smith, J. J., Morgan, P. J., Plotnikoff, R. C., Dally, K. A., Salmon, J., Okely, A. D., ... & Lubans, D. R. (2014). Smart-phone obesity prevention trial for adolescent boys in low-income communities: the ATLAS RCT. *Pediatrics*, *134*(3), e723-e731.

Smith, J. J., Morgan, P. J., Plotnikoff, R. C., Stodden, D. F., & Lubans, D. R. (2016). Mediating effects of resistance training skill competency on health-related fitness and physical activity: The ATLAS cluster randomised controlled trial. *Journal of sports sciences*, *34*(8), 772-779.

<https://www.anzctr.org.au/Trial/Registration/TrialReview.aspx?id=363015&isReview=true>

**32 Martin et al., Active Classrooms**

**References:**

Martin, R., & Murtagh, E. (2017). Active classrooms: a cluster randomized controlled trial evaluating the effects of a movement integration intervention on the physical activity levels of primary school children. *Journal of physical activity and health*, *14*(4), 290-300.

Martin, R., & Murtagh, E. M. (2015). An intervention to improve the physical activity levels of children: design and rationale of the ‘Active Classrooms’ cluster randomised controlled trial. *Contemporary clinical trials*, *41*, 180-191.

<http://www.isrctn.com/ISRCTN14265493>

**^33^ McKay et al., AS! BC**

**References:**

McKay, H. A., Macdonald, H. M., Nettlefold, L., Masse, L. C., Day, M., & Naylor, P. J. (2015). Action Schools! BC implementation: from efficacy to effectiveness to scale-up. *British journal of sports medicine*, *49*(4), 210-218.

Naylor, P. J., Macdonald, H. M., Reed, K. E., & McKay, H. A. (2006). Action Schools! BC: a socioecological approach to modifying chronic disease risk factors in elementary school children.

Naylor, P. J., Macdonald, H. M., Zebedee, J. Reed, K. E., & McKay, H. A. (2006). Lessons learned from Action Schools! BC - an ‘active school’ model to promote physical activity in elementary schools. Journal of Science and Medicine in Sport 9:413-423

**^34^ Miller et al., PLUNGE**

**References:**

Miller, A., Christensen, E. M., Eather, N., Sproule, J., Annis-Brown, L., & Lubans, D. R. (2015). The PLUNGE randomized controlled trial: Evaluation of a games-based physical activity professional learning program in primary school physical education. *Preventive medicine*, *74*, 1-8.

Miller, A., Eather, N., Gray, S., Sproule, J., Williams, C., Gore, J., & Lubans, D. (2017). Can continuing professional development utilizing a game-centred approach improve the quality of physical education teaching delivered by generalist primary school teachers?. *European Physical Education Review*, *23*(2), 171-195.

<https://www.anzctr.org.au/Trial/Registration/TrialReview.aspx?id=364311>

**^35^ Morris et al., Unnamed**

**References:**

Morris, J. L., Daly-Smith, A., Defeyter, M. A., McKenna, J., Zwolinsky, S., Lloyd, S., ... & Graham, P. L. (2019). A Pedometer-Based Physically Active Learning Intervention: The Importance of Using Preintervention Physical Activity Categories to Assess Effectiveness. *Pediatric exercise science*, *31*(3), 356-362.

**^36^ Nader et al., BEPA**

**References:**

Abi Nader, P., Hilberg, E., Schuna Jr, J. M., John, D. H., & Gunter, K. B. (2019). Association of Teacher‐Level Factors With Implementation of Classroom‐Based Physical Activity Breaks. *Journal of School Health*, *89*(6), 435-443.

**^37^ Norris et al., Virtual Traveller**

**References:**

Norris, E., Dunsmuir, S., DUnited Kingdome-Williams, O., Stamatakis, E., & Shelton, N. (2018). Physically active lessons improve lesson activity and on-task behavior: A cluster-randomized controlled trial of the “Virtual Traveller” Intervention. *Health Education & Behavior*, *45*(6), 945-956.

Norris, E., Dunsmuir, S., DUnited Kingdome-Williams, O., Stamatakis, E., & Shelton, N. (2016). Protocol for the ‘Virtual Traveller’cluster-randomised controlled trial: a behaviour change intervention to increase physical activity in primary-school Maths and English lessons. *BMJ open*, *6*(6), e011982.

Norris, E., Dunsmuir, S., DUnited Kingdome-Williams, O., Stamatakis, E., & Shelton, N. (2018). Mixed method evaluation of the Virtual Traveller physically active lesson intervention: An analysis using the RE-AIM framework. *Evaluation and program planning*, *70*, 107-114.

**^38^ O’Leary et al., Project Spraoi**

O’Leary, M., Rush, E., Lacey, S., Burns, C., & Coppinger, T. (2019). Project Spraoi: two year outcomes of a whole school physical activity and nutrition intervention using the RE-AIM framework. *Irish Educational Studies*, *38*(2), 219-243.

Coppinger, T., Lacey, S., O'Neill, C., & Burns, C. (2016). ‘Project Spraoi’: A randomized control trial to improve nutrition and physical activity in school children. *Contemporary Clinical Trials Communications*, *3*, 94-101.

<http://www.isrctn.com/ISRCTN92611015>

**^39^ O'Neill et al., Michigan Model for Health**

**References:**

O'Neill, J. M., Clark, J. K., & Jones, J. A. (2016). Promoting fitness and safety in elementary students: a randomized control study of the Michigan model for health. *Journal of school health*, *86*(7), 516-525.

**^40^ Okely et al., Girls in Sport**

**References:**

Okely, A. D., Lubans, D. R., Morgan, P. J., Cotton, W., Peralta, L., Miller, J., ... & Janssen, X. (2017). Promoting physical activity among adolescent girls: the girls in sport group randomized trial. *International Journal of behavioral nutrition and physical activity*, *14*(1), 81.

Okely, A. D., Cotton, W. G., Lubans, D. R., Morgan, P. J., Puglisi, L., Miller, J., ... & Perry, J. (2011). A school-based intervention to promote physical activity among adolescent girls: rationale, design, and baseline data from the girls in sport group randomised controlled trial. *BMC public health*, *11*(1), 1-11.

Okely, A. D., Wright, J., Puglisi, L., Batterham., M., Janssen., X., Lubans, D. R., Morgan, P. J., Cotton, W. G., Peralta, L., Perry., J. (2012). Girls in Sport Intervention and Research Project. *Summary Report for the NSW Department of Education and Communities prepared by the Research Consortium.*

<http://www.anzctr.org.au/Trial/Registration/TrialReview.aspx?id=336305&isReview=true>

**^41^ Riley et al., E.A.S.Y. Minds**

**References:**

Riley, N., Lubans, D. R., Holmes, K., & Morgan, P. J. (2016). Findings from the EASY minds cluster randomized controlled trial: evaluation of a physical activity integration program for mathematics in primary schools. *Journal of Physical Activity and Health*, *13*(2), 198-206.

Riley, N., Lubans, D. R., Holmes, K., & Morgan, P. J. (2014). Rationale and study protocol of the EASY Minds (Encouraging Activity to Stimulate Young Minds) program: cluster randomized controlled trial of a primary school-based physical activity integration program for mathematics. *BMC Public Health*, *14*(1), 816.

Mavilidi, M. F., Lubans, D. R., Miller, A., Eather, N., Morgan, P. J., Lonsdale, C., . . . Riley, N. (2020). Impact of the “Thinking while Moving in English” intervention on primary school children's academic outcomes and physical activity: A cluster randomised controlled trial. International Journal of Educational Research, 102. doi:[10.1016/j.ijer.2020.101592](http://doi.org/10.1016/j.ijer.2020.101592)

<https://www.anzctr.org.au/Trial/Registration/TrialReview.aspx?id=364344>

**^42^ Robertson et al., FitQuest**

**References:**

Robertson, J., Macvean, A., Fawkner, S., Baker, G., & Jepson, R. G. (2018). Savouring our mistakes: Learning from the FitQuest project. *International journal of child-computer interaction*, *16*, 55-67.

<http://www.isrctn.com/ISRCTN11693550>

<https://judyrobertson.typepad.com/judy_robertson/data-for-exergame-for-childrens-activity-rct-and-qualitative-study-paper.html>

**^43^ Seibert et al., Unnamed**

**References:**

Seibert, T., Allen, D. B., Eickhoff, J. C., & Carrel, A. L. (2019). US Centers for Disease Control and Prevention‐Based Physical Activity Recommendations Do Not Improve Fitness in Real‐World Settings. *Journal of school health*, *89*(3), 159-164.

<https://clinicaltrials.gov/ct2/show/NCT02411552?id=NCT02411552&draw=2&rank=1>

**^44^ Smedegaard et al., Move for Wellbeing in School**

**References:**

Smedegaard, S., Brondeel, R., Christiansen, L. B., & Skovgaard, T. (2017). What happened in the ‘Move for Well-being in School’: a process evaluation of a cluster randomized physical activity intervention using the RE-AIM framework. *International Journal of Behavioral Nutrition and Physical Activity*, *14*(1), 159.

Smedegaard, S., Christiansen, L. B., Lund-Cramer, P., Bredahl, T., & Skovgaard, T. (2016). Improving the well-being of children and youths: a randomized multicomponent, school-based, physical activity intervention. *BMC Public Health*, *16*(1), 1127.

<http://www.isrctn.com/ISRCTN12496336>

**^45^ Sutherland et al., Unnamed**

**References:**

Sutherland, R. L., Nathan, N. K., Lubans, D. R., Cohen, K., Davies, L. J., Desmet, C., ... & Wolfenden, L. (2017). An RCT to facilitate implementation of school practices known to increase physical activity. *American Journal of Preventive Medicine*, *53*(6), 818-828.

<https://www.anzctr.org.au/Trial/Registration/TrialReview.aspx?id=368420>

**^46^ Tarp et al., LCoMotion**

**References:**

Tarp, J., Domazet, S. L., Froberg, K., Hillman, C. H., Andersen, L. B., & Bugge, A. (2016). Effectiveness of a school-based physical activity intervention on cognitive performance in Danish adolescents: lcomotion—learning, cognition and motion-a cluster randomized controlled trial. *PloS one*, *11*(6), e0158087.

Bugge, A., Tarp, J., Østergaard, L., Domazet, S. L., Andersen, L. B., & Froberg, K. (2014). LCoMotion-Learning, Cognition and Motion; a multicomponent cluster randomized school-based intervention aimed at increasing learning and cognition-rationale, design and methods. *BMC public health*, *14*(1), 1-8.

<https://clinicaltrials.gov/ct2/show/NCT02012881?term=NCT02012881&draw=2&rank=1>

**^47^ Tymms et al., the MOVE Project**

**References:**

Tymms, P. B., Curtis, S. E., Routen, A. C., Thomson, K. H., Bolden, D. S., Bock, S., ... & Summerbell, C. D. (2016). Clustered randomised controlled trial of two education interventions designed to increase physical activity and well-being of secondary school students: the MOVE Project. *BMJ open*, *6*(1).

<http://www.isrctn.com/ISRCTN82956355>

[http://www.move-project.org.United Kingdom/](http://www.move-project.org.uk/)

**^48^ van den Berg et al., Unnamed**

**References:**

van den Berg, V., Singh, A. S., Komen, A., Hazelebach, C., van Hilvoorde, I., & Chinapaw, M. J. (2019). Integrating juggling with math lessons: A randomized controlled trial assessing effects of physically active learning on maths performance and enjoyment in primary school children. *International journal of environmental research and public health*, *16*(14), 2452.

**^49^ Verloigne et al., Unnamed**

**References:**

Verloigne, M., Ridgers, N. D., De Bourdeaudhuij, I., & Cardon, G. (2018). Effect and process evaluation of implementing standing desks in primary and secondary schools in Belgium: a cluster-randomised controlled trial. *International Journal of Behavioral Nutrition and Physical Activity*, *15*(1), 94.

<https://clinicaltrials.gov/ct2/show/NCT03163004?term=standing+desks&cntry=BE&rank=1>

**^50^ Vik et al., UP4FUN**

**References:**

Vik, F. N., Lien, N., Berntsen, S., De Bourdeaudhuij, I., Grillenberger, M., Manios, Y., ... & Bere, E. (2015). Evaluation of the UP4FUN intervention: a cluster randomized trial to reduce and break up sitting time in European 10-12-year-old children. *PloS one*, *10*(3), e0122612.

Nanna Lien, Frøydis N. Vik, Sveinung Berntsen, Johannes Brug, Ilse De Bourdeaudhuij, Mai JM Chinapaw, Monika Grillenberger, Éva Kovács, Lea Maes, yannis manios, George Moschonis, Maartje M. van Stralen, Corinna Willhöft, Elling Bere: UP4FUN A school-based and family-involved intervention to reduce and break up sitting time among European 10-12 year olds - systematic development and formative evaluation. 04/2014; Universitetet i Agder. (Working Paper 165). http://hdl.handle.net/11250/194424., ISBN: 1504-9280

<http://www.isrctn.com/ISRCTN34562078>

**^51^ Wright et al., FLEX**

**References:**

Wright, C. M., Chomitz, V. R., Duquesnay, P. J., Amin, S. A., Economos, C. D., & Sacheck, J. M. (2019). The FLEX study school-based physical activity programs-measurement and evaluation of implementation. *BMC public health*, *19*(1), 73.

Wright, C. M., Duquesnay, P. J., Anzman-Frasca, S., Chomitz, V. R., Chui, K., Economos, C. D., ... & Sacheck, J. M. (2016). Study protocol: the Fueling Learning through Exercise (FLEX) study-a randomized controlled trial of the impact of school-based physical activity programs on children’s physical activity, cognitive function, and academic achievement. *BMC Public Health*, *16*(1), 1-12.

<https://clinicaltrials.gov/ct2/show/NCT02810834>

**^52^ Zhou et al., Chinese CHAMPS**

**References:**

Zhou, Z., Li, S., Yin, J., Fu, Q., Ren, H., Jin, T., ... & Yin, Z. (2019). Impact on Physical Fitness of the Chinese CHAMPS: A Clustered Randomized Controlled Trial. *International journal of environmental research and public health*, *16*(22), 4412.

Zhou, Z., Dong, S., Yin, J., Fu, Q., Ren, H., & Yin, Z. (2018). Improving physical fitness and cognitive functions in middle school students: Study protocol for the Chinese childhood health, activity and motor performance study (Chinese CHAMPS). *International journal of environmental research and public health*, *15*(5), 976.
