# Supplementary material for: Features of effective staff training programmes within school-based interventions targeting student activity behaviour: a systematic review and meta-analysis
Source: Int J Behav Nutr Phys Act. 2022 Sep 24;19:125. doi: 10.1186/s12966-022-01361-6 (PMC9509574; doi:10.1186/s12966-022-01361-6)
Supplement: Supplementary file 7 — Additional file 7. Quality assessment ratings and classification results for fidelity outcomes. [file 12966_2022_1361_MOESM7_ESM.docx]

Additional File 7. Quality assessment ratings and classification results for fidelity outcomes

| **Lead author** | **Study name** | **Selection**  **bias** | **Study**  **design** | **Confounders** | **Blinding** | **Data collection** | **Withdrawals & drop-outs** | **Global rating** | **Fidelity classification**  **result^a^** |
| --- | --- | --- | --- | --- | --- | --- | --- | --- | --- |
| Aadland et al | ASK | Moderate | Strong | Weak | Weak | Weak | Strong | Weak | High |
| Adab et al | WAVES | Weak | Strong | Strong | Weak | Strong | Strong | Weak | Low |
| Aittasalo et al | KIDS OUT! | Strong | Strong | Strong | Weak | Weak | Strong | Weak | High |
| Anderson et al | AFLY5 | Weak | Strong | Strong | Weak | Weak | Weak | Weak | Medium |
| Chan et al | A+FMS | Weak | Strong | Weak | Moderate | Weak | Strong | Weak | High |
| Cohen et al | SCORES | Weak | Strong | Strong | Weak | Weak | Strong | Weak | Medium |
| Dyrstad et al | the Active School Study | Weak | Strong | Strong | Weak | Weak | Strong | Weak | Medium |
| Escriva-Boulley et al | No specific study name | Weak | Strong | Strong | Moderate | Strong | Weak | Weak | Excluded |
| Filho et al | Fortaleça sua Saúde | Strong | Strong | Strong | Weak | Weak | Strong | Weak | Excluded |
| Gray et al | Choice, Control and Change | Weak | Strong | Strong | Weak | Weak | Strong | Weak | Medium |
| Ha et al | No specific study name | Moderate | Strong | Strong | Moderate | Weak | Strong | Moderate | High |
| Ha et al | SELF-FIT | Weak | Strong | Strong | Weak | Weak | Strong | Weak | Medium |
| Have et al | No specific study name | Weak | Strong | Strong | Weak | Weak | Strong | Weak | Excluded |
| Hillman et al | A+PACC | Moderate | Strong | Strong | Weak | Strong | Strong | Moderate | Medium |
| Hodges et al | KIA | Weak | Strong | Weak | Weak | Weak | Strong | Weak | Medium |
| Hollis et al | PA4E1 | Moderate | Strong | Strong | Weak | Weak | Strong | Weak | Medium |
| Janssen et al | PLAYgrounds | Weak | Strong | Strong | Weak | Weak | Strong | Weak | High |
| Kelly et al | COPE TEEN | Weak | Strong | Strong | Moderate | Strong | Weak | Weak | Medium |
| Kennedy et al | Resistance Training for Teens | Weak | Strong | Strong | Weak | Weak | Strong | Weak | High |
| Kien et al | Bewegte Klasse | Moderate | Strong | Weak | Weak | Weak | Strong | Weak | Medium |
| Kocken et al | EF! | Weak | Strong | Strong | Weak | Weak | Strong | Weak | Low |
| Koykka et al | Let's Move It | Moderate | Strong | Strong | Weak | Weak | Strong | Weak | Medium |
| Lonsdale et al | AMPED | Moderate | Strong | Strong | Moderate | Weak | Strong | Moderate | Medium |
| Lubans et al | ATLAS | Moderate | Strong | Strong | Weak | Weak | Strong | Weak | Medium |
| McKay et al | AC! BC! | Weak | Strong | Weak | Weak | Weak | Weak | Weak | Excluded |
| Miller et al | PLUNGE | Moderate | Strong | Strong | Weak | Weak | Strong | Weak | Excluded |
| Norris et al | Virtual Traveller | Weak | Strong | Weak | Weak | Weak | Strong | Weak | Medium |
| Nader et al | GROW HKC | Weak | Strong | Weak | Weak | Weak | Strong | Weak | High |
| O Leary et al | Project Spraoi | Weak | Strong | Strong | Weak | Weak | Strong | Weak | Excluded |
| O Neill et al | Michigan Model for Health | Weak | Strong | Strong | Weak | Weak | Strong | Weak | High |
| Okely et al | Girls in Sport | Weak | Strong | Strong | Strong | Weak | Strong | Weak | Low |
| Riley et al | EASY Minds | Weak | Strong | Strong | Weak | Weak | Strong | Weak | High |
| Robertson et al | FitQuest | Weak | Strong | Weak | Weak | Weak | Strong | Weak | Low |
| Seibert et al | No specific study name | Moderate | Strong | Strong | Weak | Weak | Weak | Weak | Medium |
| Smedegaard et al | Move for Wellbeing in School | Weak | Strong | Strong | Weak | Weak | Strong | Weak | Medium |
| Sutherland et al | No specific study name | Moderate | Strong | Strong | Weak | Weak | Strong | Weak | Excluded |
| Tarp et al | LCoMotion | Moderate | Strong | Weak | Weak | Weak | Strong | Weak | Low |
| Tymms et al | Peer mentoring | Weak | Strong | Strong | Weak | Weak | Strong | Weak | Medium |
| Tymms et al | Participative learning | Weak | Strong | Strong | Weak | Weak | Strong | Weak | Medium |
| van den Berg et al | No specific study name | Weak | Strong | Strong | Weak | Weak | Strong | Weak | High |
| Verloigne et al | UP4FUN - The ENERGY Project | Weak | Strong | Strong | Weak | Weak | Weak | Weak | Excluded |
| Wright et al | FLEX - 100 Mile Club | Weak | Strong | Strong | Weak | Weak | Strong | Weak | Low |
| Wright et al | FLEX – CHALK/Just Move | Weak | Strong | Strong | Weak | Weak | Strong | Weak | Low |
| Zhou et al | Chinese CHAMPS | Moderate | Strong | Weak | Weak | Strong | Strong | Weak | Excluded |

^a^ We employed a structured process to classify fidelity outcomes (see Additional File 2 for further details). Studies that solely reported fidelity outcomes in the absence of any quantitative goal/aim to compare outcomes against were excluded (e.g. fidelity scores were solely compared against the control group only or fidelity outcomes reported in other studies).
