# Supplementary material for: Features of effective staff training programmes within school-based interventions targeting student activity behaviour: a systematic review and meta-analysis
Source: Int J Behav Nutr Phys Act. 2022 Sep 24;19:125. doi: 10.1186/s12966-022-01361-6 (PMC9509574; doi:10.1186/s12966-022-01361-6)
Supplement: Supplementary file 8 — Additional file 8. Quality assessment ratings for activity behaviour outcomes (physical activity and sedentary behaviour combined). [file 12966_2022_1361_MOESM8_ESM.docx]

Additional File 8. Quality assessment ratings for activity behaviour outcomes (physical activity and sedentary behaviour combined)

| **Lead author** | **Trial name** | **Selection**  **bias** | **Study**  **design** | **Confounders** | **Blinding** | **Data collection** | **Withdrawals & drop-outs** | **Global rating** |
| --- | --- | --- | --- | --- | --- | --- | --- | --- |
| Aadland et al | ASK | Moderate | Strong | Strong | Weak | Strong | Strong | Moderate |
| Adab et al | WAVES | Weak | Strong | Strong | Moderate | Strong | Strong | Moderate |
| Anderson et al | AFLY5 | Strong | Strong | Strong | Moderate | Strong | Weak | Moderate |
| Belton et al | Y-PATH | Strong | Strong | Strong | Weak | Strong | Weak | Weak |
| Bundy et al | Sydney Playground Project | Moderate | Strong | Strong | Weak | Strong | Strong | Moderate |
| Christiansen et al | SPACE | Moderate | Strong | Strong | Weak | Strong | Strong | Moderate |
| Cohen et al | SCORES | Moderate | Strong | Strong | Weak | Strong | Weak | Weak |
| Drummy et al | No specific trial name | Weak | Strong | Strong | Weak | Strong | Weak | Weak |
| Dyrstad et al | the Active School Study | Moderate | Strong | Strong | Weak | Strong | Strong | Moderate |
| Ha et al | No specific trial name | Moderate | Strong | Strong | Strong | Strong | Strong | Strong |
| Ha et al | SELF-FIT | Weak | Strong | Strong | Moderate | Strong | Strong | Moderate |
| Harrington et al | Girls Active | Moderate | Strong | Strong | Moderate | Strong | Moderate | Strong |
| Hollis et al | PA4E1 | Strong | Strong | Strong | Moderate | Strong | Strong | Strong |
| Janssen et al | PLAYgrounds | Weak | Strong | Strong | Weak | Strong | Weak | Weak |
| Kennedy et al | Resistance Training for Teens | Weak | Strong | Strong | Weak | Strong | Moderate | Moderate |
| Kocken et al | EF! | Weak | Strong | Strong | Weak | Weak | Moderate | Weak |
| Lonsdale et al | AMPED | Moderate | Strong | Weak | Strong | Strong | Strong | Moderate |
| Lubans et al | ATLAS | Moderate | Strong | Strong | Moderate | Strong | Moderate | Strong |
| Martin et al | Active Classrooms | Moderate | Strong | Strong | Weak | Strong | Strong | Moderate |
| Miller et al | PLUNGE | Moderate | Strong | Strong | Moderate | Strong | Strong | Strong |
| Morris et al | No specific trial name | Weak | Strong | Strong | Weak | Strong | Strong | Weak |
| Norris et al | Virtual Traveller | Weak | Strong | Strong | Weak | Strong | Moderate | Weak |
| Okely et al | Girls in Sport | Weak | Strong | Strong | Strong | Strong | Strong | Moderate |
| Riley et al | EASY Minds | Weak | Strong | Strong | Weak | Strong | Strong | Weak |
| Robertson et al | FitQuest | Weak | Strong | Strong | Weak | Strong | Strong | Weak |
| Tarp et al | LCoMotion | Moderate | Strong | Strong | Weak | Strong | Strong | Moderate |
| Zhou et al | Chinese CHAMPS | Moderate | Strong | Strong | Moderate | Strong | Moderate | Strong |
