# Supplementary material for: Features of effective staff training programmes within school-based interventions targeting student activity behaviour: a systematic review and meta-analysis
Source: Int J Behav Nutr Phys Act. 2022 Sep 24;19:125. doi: 10.1186/s12966-022-01361-6 (PMC9509574; doi:10.1186/s12966-022-01361-6)
Supplement: Supplementary file 9 — Additional file 9. Forest and funnel plots for physical activity outcomes. [file 12966_2022_1361_MOESM9_ESM.docx]

Additional File 9. Forest and funnel plots for physical activity outcomes

Figure 1. Forest plot of standardised mean difference of change in physical activity between intervention and control groups of school-based physical activity interventions

Figure 2. Funnel plot for physical activity outcomes

Figure 3. Forest plot of standardised mean difference of change in physical activity between intervention and control groups of school-based physical activity interventions, with outcomes from PLAYgrounds [1] and Chinese CHAMPS [2] removed

Figure 4. Funnel plot for physical activity outcomes with studies removed

References

1. Janssen M, Twisk JW, Toussaint HM, van Mechelen W, Verhagen EA. Effectiveness of the PLAYgrounds programme on PA levels during recess in 6-year-old to 12-year-old children. *Br J Sports Med* 2015, 49(4):259-264.

2. Zhou Z, Li S, Yin J, Fu Q, Ren H, Jin T, Zhu J, Howard J, Lan T, Yin Z. Impact on physical fitness of the chinese champs: A clustered randomized controlled trial. *Int J Environ Res Public Health* 2019, 16 (22) (no pagination)(4412).
