# Supplementary material for: Features of effective staff training programmes within school-based interventions targeting student activity behaviour: a systematic review and meta-analysis
Source: Int J Behav Nutr Phys Act. 2022 Sep 24;19:125. doi: 10.1186/s12966-022-01361-6 (PMC9509574; doi:10.1186/s12966-022-01361-6)
Supplement: Supplementary file 10 — Additional file 10. Forest plots and funnel plots for sedentary behaviour outcomes. [file 12966_2022_1361_MOESM10_ESM.docx]

Additional File 10. Forest plots and funnel plots for sedentary behaviour outcomes


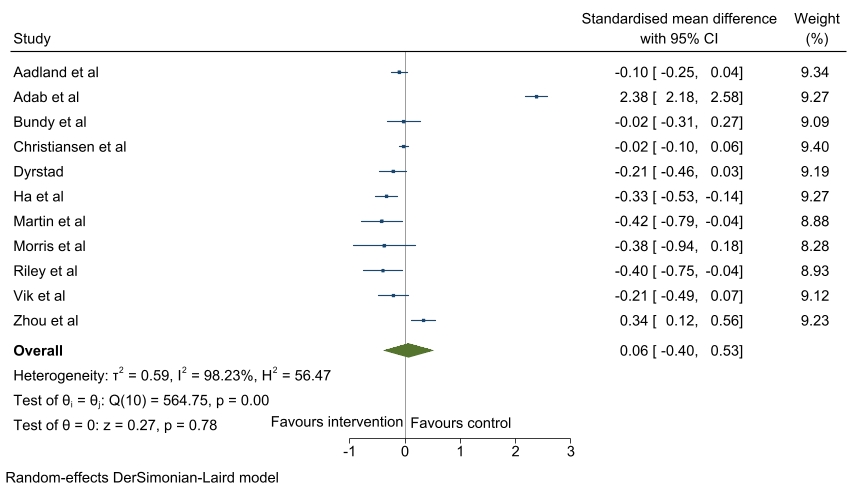


Figure 1. Forest plot of standardised mean difference of change in sedentary behaviour between intervention and control groups of school-based physical activity interventions

Figure 2. Funnel plot for sedentary behaviour outcomes
